# Mitochondrial DNA Backgrounds Might Modulate Diabetes Complications Rather than T2DM as a Whole

**DOI:** 10.1371/journal.pone.0021029

**Published:** 2011-06-09

**Authors:** Alessandro Achilli, Anna Olivieri, Maria Pala, Baharak Hooshiar Kashani, Valeria Carossa, Ugo A. Perego, Francesca Gandini, Aurelia Santoro, Vincenza Battaglia, Viola Grugni, Hovirag Lancioni, Cristina Sirolla, Anna Rita Bonfigli, Antonella Cormio, Massimo Boemi, Ivano Testa, Ornella Semino, Antonio Ceriello, Liana Spazzafumo, Maria Nicola Gadaleta, Maurizio Marra, Roberto Testa, Claudio Franceschi, Antonio Torroni

**Affiliations:** 1 Dipartimento di Biologia Cellulare e Ambientale, Università di Perugia, Perugia, Italy; 2 Dipartimento di Genetica e Microbiologia, Università di Pavia, Pavia, Italy; 3 Sorenson Molecular Genealogy Foundation, Salt Lake City, Utah, United States of America; 4 Dipartimento di Patologia Sperimentale, Università di Bologna, Bologna, Italy; 5 CIG-Interdepartmental Center for Biophysics and Biocomplexity Studies, Università di Bologna, Bologna, Italy; 6 Department of Gerontology Research, Statistic and Biometry Center, Italian National Research Center on Aging (INRCA), Ancona, Italy; 7 Metabolic and Nutrition Research Center on Diabetes, Italian National Research Center on Aging, INRCA-IRCCS, Ancona, Italy; 8 Dipartimento di Biochimica e Biologia Molecolare “E. Quagliariello”, Università di Bari, Bari, Italy; 9 Centro Interdipartimentale “Studi di Genere”, Università di Pavia, Pavia, Italy; 10 Institut d'Investigacions Biomèdiques August Pi Sunyer (IDIBAPS) and Centro de Investigacion Biomedica en Red de Diabetes y Enfermedades Metabolicas Asociadis (CIBERDEM), Barcelona, Spain; Rambam Health Care Campus, Israel

## Abstract

Mitochondrial dysfunction has been implicated in rare and common forms of type 2 diabetes (T2DM). Additionally, rare mitochondrial DNA (mtDNA) mutations have been shown to be causal for T2DM pathogenesis. So far, many studies have investigated the possibility that mtDNA variation might affect the risk of T2DM, however, when found, haplogroup association has been rarely replicated, even in related populations, possibly due to an inadequate level of haplogroup resolution. Effects of mtDNA variation on diabetes complications have also been proposed. However, additional studies evaluating the mitochondrial role on both T2DM and related complications are badly needed. To test the hypothesis of a mitochondrial genome effect on diabetes and its complications, we genotyped the mtDNAs of 466 T2DM patients and 438 controls from a regional population of central Italy (Marche). Based on the most updated mtDNA phylogeny, all 904 samples were classified into 57 different mitochondrial sub-haplogroups, thus reaching an unprecedented level of resolution. We then evaluated whether the susceptibility of developing T2DM or its complications differed among the identified haplogroups, considering also the potential effects of phenotypical and clinical variables. MtDNA backgrounds, even when based on a refined haplogroup classification, do not appear to play a role in developing T2DM despite a possible protective effect for the common European haplogroup H1, which harbors the G3010A transition in the MTRNR2 gene. In contrast, our data indicate that different mitochondrial haplogroups are significantly associated with an increased risk of specific diabetes complications: H (the most frequent European haplogroup) with retinopathy, H3 with neuropathy, U3 with nephropathy, and V with renal failure.

## Introduction

The etiology of type 2, or non-insulin-dependent, diabetes mellitus (T2DM), the most common metabolic disease in the Western hemisphere, is the result of an interaction of environmental factors with a combination of genetic variants, most of which are still unknown. Case-control studies can be used to predict and quantify the association of genetic factors and lifestyle with some common diseases, thus contributing to the body of knowledge of primary prevention for these conditions. Different genome-wide association studies have led to recent discoveries of novel diabetes-related nuclear loci [1]–[3], which often fail to be replicated and confirmed in different populations, possibly because of population-specific susceptibility patterns [4]. This may reflect differences in the genetic structure of human populations, each with its peculiar evolutionary history, for instance turning minor alleles in a certain population to prevalent ones in another population, and thus deeply affecting the frequencies of allele combinations.

Obviously, the possibility of obtaining false positive/negative results is greatly decreased when patients and controls come from the same population and/or geographic area where the genetic background should be more homogenous. This is particularly true when association studies involve a genetic system such as the maternally transmitted mitochondrial DNA (mtDNA), whose worldwide “natural” sequence variation is geographically and ethnically differentiated. MtDNA haplotypes and haplogroups (groups of mtDNA haplotypes sharing the same mutational motifs by descent from a common female ancestor) are extremely common in one continent or even a single geographic area/population group, but completely absent in all others [5], [6].

Mitochondrial involvement in the pathogenesis of major common metabolic disorders, including T2DM, stems from the observation of dysfunctions in the mitochondrial energy production machinery (OXPHOS) of many patients [7]–[10]. Single mtDNA mutations, including both major rearrangements and point mutations, and/or mitochondrial haplogroups have been associated with conditions of type 2 diabetes. For instance a protective role has been attributed to the Asian haplogroup N9a [11], [12] and to the Western Eurasian haplogroup J1 [13], while the super-haplogroup J/T and the T haplogroup alone have been associated with an increased risk of diabetes in Europeans [14]. Yet, most studies either failed to report definitive associations between T2DM and variation in the mitochondrial genome, or even conflicting results have been observed [15]–[17]. Thus, all associations reported so far between mtDNA mutations (or haplogroups) and diabetes, with the exception of the rare mutations associated with Maternally Inherited Diabetes and Deafness (MIDD, [MIM #520000]), remain provisional (see Mitomap for a review http://www.mitomap.org/MITOMAP). This is not a feature restricted to T2DM. Indeed, with the exception of Leber Hereditary Optic Neuropathy (LHON), the association between disorders and mtDNA haplogroups has rarely been replicated by studies in other populations [18], [19]. Association studies can be confounded if patients and controls are poorly characterized or not well matched. However, mtDNA association studies are probably affected also by another major specific problem: the level of resolution employed in the classification of mtDNA haplogroups has been generally very low. Recent studies confirm that mtDNA association analyses performed so far have often been too simplistic. A striking example is represented by haplogroup H, by far the most common haplogroup in Europe with a uniformly high frequency (30%–50%), which is formed by many sub-haplogroups whose frequencies vary considerably across Europe [20], [21]. Such a degree of difference in frequency distribution of mtDNA sub-haplogroups alone could easily explain some of the inconsistent results obtained by association studies carried out in different European populations.

A second general area of investigation concerns the role of mtDNA variants or haplogroups in modulating susceptibility to develop diabetes complications, usually classified into macrovascular (cardiovascular disease, cerebrovascular accidents, and peripheral vascular disease) and microvascular complications (diabetic nephropathy, neuropathy, and retinopathy) [22], [23], but the number of mtDNA studies that, in addition to the "whole" T2DM phenotype, have also evaluated diabetes complications is still limited [24]–[26].

In this study, to evaluate the role of mtDNA backgrounds, not only in T2DM as a whole but also in its associated complications, we have genotyped, at an extremely high level of phylogenetic resolution, the mitochondrial genome of a large number of subjects (466 T2DM patients and 438 controls) from the Marche region of central Italy.

## Results

Before determining the extent and nature of mtDNA variation in control and diabetic subjects from the Marche region, we investigated the effects of a large number of phenotypical and clinical variables on the risk of T2DM. Some of the traits evaluated in the 904 subjects are shown in Table 1. No difference in the risk of T2DM was found only for fibrinogen and C-reactive protein. As expected, among the strongest predictors of diabetes were age, BMI (and consequently obesity), metabolic syndrome, insulin resistance evaluated by homeostasis model assessment (HOMA), glycated hemoglobin (HbA1c), high-density lipoprotein (HDL) cholesterol, triglycerides, and hypertension. All these parameters, except HDL cholesterol, were much higher in patients than in controls. However, there was also one unexpected finding, also in light of the randomly selected gender of participants: a significantly increased risk for males and smokers was noted (*p-values* of <0.0001 and 0.001, respectively). Assuming that BMI might be considered as a general indicator of health, we found that the BMI is just slightly lower among smokers (27.3±4.2 *vs* 28.2±4.7).

**Table 1:** Clinical characteristics of diabetic patients and controls.

		All samples^a^			Males^a^			Females^a^			
	Variables	Patients (N = 466)	Controls (N = 438)	*p-value*	Patients (N = 257)	Controls (N = 176)	*p-value*	Patients (N = 209)	Controls (N = 262)	*p-value*	N analyzed (Males;Females)
**Traits:**	Age [years]	65.84±8.19	59.96±9.97	<0.0001	65.09±8.55	59.34±9.40	<0.0001	66.76±7.64	60.38±10.32	<0.0001	904 (433;471)
	Sex [M/F (%)]	257/209 (55.15%/44.85%)	176/262 (40.18%/59.82%)	<0.0001							
	BMI [Kg/m2]	28.76±4.62	27.14±4.46	<0.0001	28.24±4.15	27.29±3.51	0.0107	29.40±5.07	27.04±5.00	<0.0001	902 (432;470)
	Obesity^b^ (%)	152 (32.69%)	96 (21.97%)	0.0003	71 (27.73%)	35 (19.89%)	0.0696	81 (38.76%)	61 (23.37%)	0.0004	902 (432;470)
	Smokers (%)	124 (28.64%)	76 (16.14%)	0.0010	92 (35.80%)	32 (18.18%)	<0.0001	36 (17.22%)	40 (15.27%)	0.6147	904 (433;471)
	Metabolic Syndrome^c^ (%)	267 (57.42%)	56 (12.90%)	<0.0001	114 (44.53%)	16 (9.25%)	<0.0001	153 (73.21%)	40 (15.33%)	<0.0001	899 (429;470)
	Insulin Resistance^d^ (%)	190 (40.77%)	54 (12.47%)	<0.0001	97 (37.74%)	26 (15.12%)	<0.0001	93 (44.50%)	28 (10.73%)	<0.0001	899 (429;470)
	Fibrinogen [mg/dL]	303.50±79.49	295.29±71.76	0.1590	294.04±77.85	281.95±78.17	0.2012	315.36±80.11	302.89±66.90	0.1064	706 (342;364)
	C-reactive protein [mg/L]	4.55±7.01	3.43±7.37	0.0200	4.20±6.74	3.62±10.45	0.5179	4.97±7.31	3.30±4.17	0.0035	901 (431;470)
	HbA1c [%]	7.43±1.25	5.67±0.40	<0.0001	7.37±1.28	5.59±0.36	<0.0001	7.51±1.20	5.72±0.42	<0.0001	904 (433;471)
	HDL [mg/dL]	52.75±14.85	58.32±15.17	<0.0001	49.44±12.10	51.77±13.50	0.0682	56.82±16.80	62.64±14.68	<0.0001	901 (430;471)
	Triglycerides	138.38±93.16	103.28±68.45	<0.0001	136.12±101.92	119.09±88.70	0.0674	141.16±81.26	92.85±48.34	<0.0001	899 (429;470)
	Hypertension (%)	293 (62.88%)	137 (31.35%)	<0.0001	155 (60.31%)	40 (22.86%)	<0.0001	138 (66.03%)	97 (37.02%)	<0.0001	903 (432;471)
**Complications:**	Retinopathy (%)	132 (28.33%)	···	···	69 (26.85%)	···	···	63 (30.14%)	···	···	904 (433;471)
	Somatic Neuropathy (%)	94 (20.17%)	···	···	64 (24.90%)	···	···	30 (14.35%)	···	···	904 (433;471)
	Cardiac Ischemia (%)	81 (8.96%)	···	···	50 (19.46%)	···	···	31 (14.83%)	···	···	904 (433;471)
	Nephropathy (%)	64 (13.73%)	···	···	45 (17.51%)	···	–	19 (9.09%)	···	···	904 (433;471)
	Peripheral Artery Occlusive Disease (PAOD)(%)	30 (3.2%)	···	···	17 (6.61%)	···	···	13 (6.22%)	···	···	904 (433;471)
	Renal Failure (%)	20 (2.21%)	···	···	15 (5.84%)	···	···	5 (2.39%)	···	···	904 (433;471)
**Index:**	HOMA-IR	2.99±3.52	1.59±1.55	<0.0001	3.14±4.22	1.74±1.78	<0.0001	2.81±2.39	1.49±1.36	<0.0001	904 (433;471)

a Values are means ± standard deviation (or absolute number and percentages).

b BMI ≥30.

c Diagnosed according to the criteria proposed by the National Cholesterol Education Program (NCEP) Adult Treatment Panel III (ATP III).

d HOMA-IR >2.5.

### T2DM and mtDNA haplogroups

All 904 mtDNAs were genotyped and assigned to 57 different mtDNA haplogroups and sub-haplogroups (Table S1). This classification was based on the most updated phylogeny [27], thus reaching an unprecedented level of resolution. As summarized in Table 2, more than 97% of the mtDNAs belonged to typical Western Eurasian lineages, i.e. H*, H1, H3, H5, H6, H8, H9, HV*, HV0, V, R0a, J1, J2, T1, T2, U1, U2, U3, U4, U5, U6, U7, U8, U9, K1, K2, N1, I, W, X2. The only notable exceptions were represented by 24 mtDNAs: 8 attributed to the Eastern Asian clade D4 and 16 classified into three typical African haplogroups (M1, L1b and L3).

**Table 2:** Frequencies of the major mitochondrial DNA haplogroups and sub-haplogroups in diabetic patients and controls.

		All samples		Males		Females		
Haplogroup^a^		Patients (%)	Controls (%)	Patients (%)	Controls (%)	Patients (%)	Controls (%)	Total (%)
		**N = 466**	**N = 438**	**N = 257**	**N = 176**	**N = 209**	**N = 262**	**N = 904**
**H:**		161 (34.55%)	181 (41.34%)	90 (35.01%)	66 (37.50%)	71 (33.96%)	115 (43.89%)	342 (37.83%)
	H*	77 (16.52%)	72 (16.44%)	43 (16.73%)	29 (16.48%)	34 (16.27%)	43 (16.41%)	149 (16.48%)
	H1	44 (9.44%)	69 (15.75%)	23 (8.95%)	23 (13.07%)	21 (10.05%)	46 (17.56%)	113 (12.50%)
	H3	10 (2.15%)	13 (2.97%)	6 (2.33%)	8 (4.55%)	4 (1.91%)	5 (1.91%)	23 (2.54%)
	H5	16 (3.43%)	17 (3.88%)	11 (4.28%)	3 (1.70%)	5 (2.39%)	14 (5.34%)	33 (3.65%)
	H6	10 (2.15%)	7 (1.60%)	5 (1.95%)	2 (1.14%)	5 (2.39%)	5 (1.91%)	17 (1.88%)
	H8	…	2 (0.46%)	…	1 (0.57%)	…	1 (0.38%)	2 (0.22%)
	H9	4 (0.86%)	1 (0.23%)	2 (0.78%)	…	2 (0.96%)	1 (0.38%)	5 (0.55%)
**HV** b **:**		37 (7.94%)	29 (6.62%)	25 (9.73%)	13 (7.39%)	12 (5.74%)	16 (6.11%)	66 (7.30%)
	HV*	15 (3.22%)	17 (3.88%)	8 (3.11%)	9 (5.11%)	7 (3.35%)	8 (3.05%)	32 (3.54%)
	HV0	4 (0.86%)	1 (0.23%)	3 (1.17%)	…	1 (0.48%)	1 (0.38%)	5 (0.55%)
	V	18 (3.86%)	11 (2.51%)	14 (5.45%)	4 (2.27%)	4 (1.91%)	7 (2.67%)	29 (3.21%)
**R0:**		6 (1.29%)	…	3 (1.17%)	…	3 (1.44%)	…	6 (0.66%)
	R0a	6 (1.29%)	…	3 (1.17%)	…	3 (1.44%)	…	6 (0.66%)
**J:**		33 (7.08%)	37 (8.45%)	21 (8.17%)	19 (10.80%)	12 (5.74%)	18 (6.87%)	70 (7.75%)
	J1	27 (5.79%)	31 (7.08%)	18 (7.00%)	16 (9.09%)	9 (4.31%)	15 (5.73%)	58 (6.42%)
	J2	6 (1.29%)	6 (1.37%)	3 (1.17%)	3 (1.70%)	3 (1.44%)	3 (1.15%)	12 (1.33%)
**T:**		71 (15.24%)	57 (13.01%)	37 (14.39%)	23 (13.07%)	34 (16.27%)	34 (12.98%)	128 (14.16%)
	T1	12 (2.58%)	11 (2.51%)	7 (2.72%)	3 (1.70%)	5 (2.39%)	8 (3.05%)	23 (2.54%)
	T2	59 (12.66%)	46 (10.50%)	30 (11.67%)	20 (11.36%)	29 (13.88%)	26 (9.92%)	105 (11.62%)
**UK:**								
	U	80 (17.17%)	65 (14.84%)	48 (18.68%)	25 (14.20%)	32 (15.31%)	40 (15.26%)	145 (16.04%)
	U1	3 (0.64%)	4 (0.91%)	3 (1.17%)	2 (1.14%)	…	2 (0.76%)	7 (0.77%)
	U2	1 (0.21%)	1 (0.23%)	1 (0.39%)	…	…	1 (0.38%)	2 (0.22%)
	U3	13 (2.79%)	11 (2.51%)	10 (3.89%)	4 (2.27%)	3 (1.44%)	7 (2.67%)	24 (2.65%)
	U4	12 (2.58%)	8 (1.83%)	6 (2.33%)	5 (2.84%)	6 (2.87%)	3 (1.15%)	20 (2.21%)
	U5	39 (8.37%)	34 (7.76%)	21 (8.17%)	10 (5.68%)	18 (8.61%)	24 (9.16%)	73 (8.08%)
	U6	2 (0.43%)	1 (0.23%)	…	1 (0.57%)	2 (0.96%)	…	3 (0.33%)
	U7	4 (0.86%)	2 (0.46%)	2 (0.78%)	1 (0.57%)	2 (0.96%)	1 (0.38%)	6 (0.66%)
	U8	5 (1.07%)	4 (0.91%)	4 (1.56%)	2 (1.14%)	1 (0.48%)	2 (0.76%)	9 (1.00%)
	U9	1 (0.21%)	…	1 (0.39%)	…	…	…	1 (0.11%)
	K	31 (6.65%)	24 (5.48%)	12 (4.67%)	15 (8.52%)	19 (9.09%)	9 (3.44%)	55 (6.08%)
	K1	30 (6.44%)	22 (5.02%)	12 (4.67%)	13 (7.39%)	18 (8.61%)	9 (3.44%)	52 (5.75%)
	K2	1 (0.21%)	2 (0.46%)	…	2 (1.14%)	1 (0.48%)	…	3 (0.33%)
**N1:**		17 (3.65%)	9 (2.05%)	9 (3.50%)	1 (0.57%)	8 (3.83%)	8 (3.05%)	26 (2.88%)
	N1	9 (1.93%)	6 (1.37%)	6 (2.33%)	1 (0.57%)	3 (1.44%)	5 (1.91%)	15 (1.66%)
	I	8 (1.72%)	3 (0.68%)	3 (1.17%)	…	5 (2.39%)	3 (1.15%)	11 (1.22%)
**N2:**		6 (1.29%)	8 (1.83%)	3 (1.17%)	4 (2.27%)	3 (1.44%)	4 (1.53%)	14 (1.55%)
	W	6 (1.29%)	8 (1.83%)	3 (1.17%)	4 (2.27%)	3 (1.44%)	4 (1.53%)	14 (1.55%)
**X:**		13 (2.79%)	15 (3.42%)	4 (1.56%)	5 (2.84%)	9 (4.31%)	10 (3.82%)	28 (3.10%)
	X2	13 (2.79%)	15 (3.42%)	4 (1.56%)	5 (2.84%)	9 (4.31%)	10 (3.82%)	28 (3.10%)
**M:**		10 (2.15%)	8 (1.82%)	5 (1.95%)	3 (1.70%)	5 (2.39%)	5 (1.91%)	18 (1.99%)
	D4	5 (1.07%)	3 (0.68%)	4 (1.56%)	2 (1.14%)	1 (0.48%)	1 (0.38%)	8 (0.88%)
	M1	5 (1.07%)	5 (1.14%)	1 (0.39%)	1 (0.57%)	4 (1.91%)	4 (1.53%)	10 (1.11%)
**L:**		1 (0.21%)	5 (1.14%)	…	2 (1.14%)	1 (0.48%)	3 (1.15%)	6 (0.66%)
	L1b	…	1 (0.23%)	…	…	…	1 (0.38%)	1 (0.11%)
	L3	1 (0.21%)	4 (0.91%)	…	2 (1.14%)	1 (0.48%)	2 (0.76%)	5 (0.55%)

a H* is a paragroup that encompasses all H mtDNAs that did not belong to any of the tested subclades of H.

b Without H.

When we compared haplogroup distributions of T2DM cases and controls (Table 2), multiple logistic-regression analysis showed that subjects harboring haplogroup H1 (9.4% and 15.8% in patients and controls, respectively) might be characterized by a reduced risk of T2DM (OR = 0.5576, 95% CI: 0.3726–0.8344, *p-value* = 0.0045), and that H1 was indeed the only haplogroup included in the final step-wise model (*p-value* = 0.004). However, such a *p-value* does not reach the statistical significance established at α≤0.003, after the Bonferroni correction. We then examined whether the possible protective effect of haplogroup H1 towards T2DM was related to age, gender, obesity, smoking, and/or to some clinical traits such as BMI, metabolic syndrome, insulin resistance, fibrinogen, C-reactive protein, HbA1c, HDL, triglycerides and hypertension. None of the parameters showed significant differences between the subjects with haplogroup H1 and those without it (data not shown).

**Figure 1:**
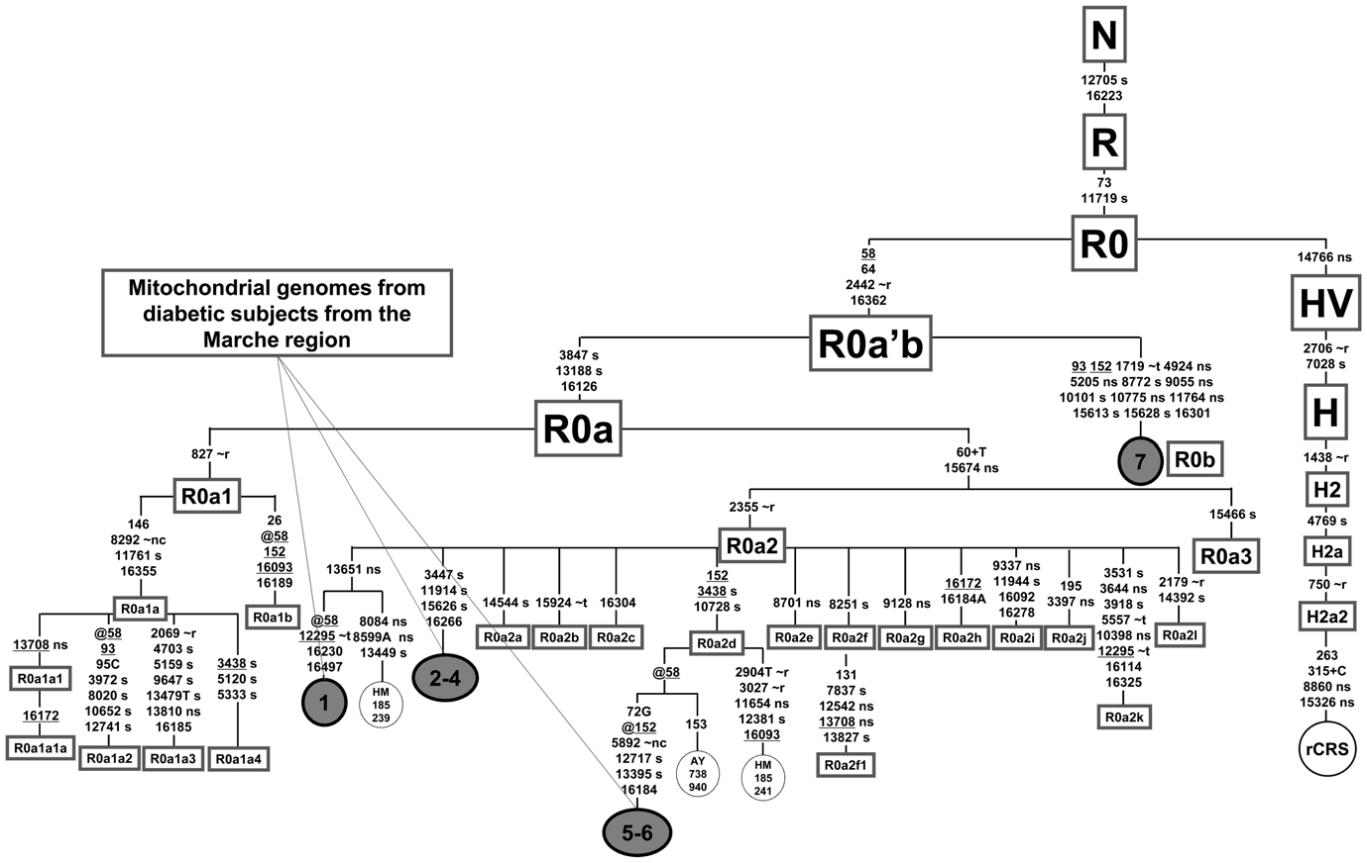
Schematic phylogeny of haplogroup R0a encompassing seven complete mtDNA sequences from diabetic patients. The schematic classification of the R0a sub-clades is based on Černý et al. [42], while the R0a'b node is newly defined on the basis of the complete genome #7. Sequences #1–7 were obtained in the course of this study and are from Italian diabetic patients: sequences #1–6 (GenBank accession numbers JF717355-JF717360) are from subjects of the Marche region sample, while sequence #7 (GenBank accession number JF717361) is from a diabetic patient not included in the current study because the maternal ancestry was from a different region (Campania, Southern Italy). Three control mtDNA sequences from the literature (GenBank accession numbers HM185239, HM185241 [42] and AY738940 [20]), which clustered in the same sub-branches of the sequences obtained from the diabetic subjects, were also included in the tree. The position of the revised Cambridge Reference Sequence (rCRS) [43] – a member of haplogroup H2a2 – is indicated for reading off sequence motifs. Mutations are shown on the branches; they are transitions unless a base is explicitly indicated. The prefix @ designates reversions, while suffixes indicate transversions (to A, G, C, or T), insertions (+), gene locus (∼t, tRNA; ∼r, rRNA; nc, non coding region outside of the control region) and synonymous or non synonymous changes (s or ns). Recurrent mutations within the phylogeny are underlined.

Table 2 illustrates a second interesting finding. Haplogroup R0a, which was not included in the logistic-regression analysis because of its low frequency (0.7%), was detected only in diabetic patients (six out of six R0a mtDNAs), thus raising the possibility of a potential effect by this rare haplogroup. The presence of three different R0a control-region haplotypes among the six subjects (Table S1) excludes the possibility of a founder event. Two possible scenarios can be envisioned to explain the detection of this haplogroup only in diabetic patients: (i) R0a might actually increase the risk of T2DM, consequently decreasing its detection rate in healthy subjects older than 40 years; (ii) the mtDNA background which may be modulating the appearance of diabetes is not the entire haplogroup R0a, but only one of its internal branches. To discriminate between the two alternative scenarios, we completely sequenced and included all six R0a mtDNAs in an updated R0a phylogeny (Figure 1). Data from the complete sequencing show that all six genomes from Marchigian diabetic patients belong to the same sub-clade named R0a2. This clade differs from the root of haplogroup R0a by three mutations. A T insertion in the control region at nucleotide position (np) 60 and the non-synonymous transition MTCYB-T15674C/S310P are shared with the sister branch R0a3, whereas the third mutation, the transition A2355G in the 16S rRNA gene, is distinctive of R0a2 (Figure 1).

### T2DM complications and mitochondrial haplogroups

Many studies have evaluated mtDNA variation in T2DM patients. However, only a few studies have tested mtDNA haplogroups for association with diabetes complications. In an attempt to investigate a potential role of mtDNA backgrounds in complications rather than in T2DM as a whole, we evaluated this issue in our population sample. Table 3 reports the haplogroup distributions observed in patients with only the diabetic phenotype and no complications and in T2DM patients characterized by the development of at least one (or at least two) of six common T2DM complications. These complications include the following: retinopathy, somatic neuropathy, nephropathy, renal failure, cardiac ischemia, and peripheral artery occlusive disease (see Tables S2, S3, S4, S5, S6, and S7 for details concerning each complication). Even if T2DM complications were determined by different molecular mechanisms, the concomitant analysis of grouped complications provides some initial clues concerning the role of mitochondrial haplogroups in modulating the pathology course. Then, this is further investigated by analyzing each candidate haplogroup in relation to patients’ traits and complications. Evidence of association was observed only for haplogroup H3 that seemed to increase the probability of developing at least one complication by almost 8.5 fold (Table 4). In particular, when we inverted the analysis, only neuropathy turned out to be related to H3 (*p-value* = 0.0007) (Table 5). The significance of the association with H3 was confirmed in the logistic regression with two or more complications, but also other groupings – the paragroup H* and the haplogroups U3 and V – turned out to be risk factors (Table 4). Considering the combined effect of H* and H3, it is conceivable that the entire haplogroup H (and its basal mutational motif) might play an important role in the development of T2DM-related complications. This scenario is supported by the finding that the incidence of retinopathy (Table S2) was significantly increased (*p-value* = 0.0007) in subjects harboring H mtDNAs (OR = 2.0075, 95% CI: 1.3080-3.0812, *p-value* 0.0014), who also showed a slight decrease in HDL cholesterol (50.73±12.22 mg/dL *vs.* 53.82±15.98 mg/dL, t-test *p-value* = 0.0202).

**Table 3:** Frequencies of mtDNA haplogroups and sub-haplogroups in patients with only the diabetic phenotype and diabetic patients also affected by at least one or two of six common complications [retinopathy, somatic neuropathy, cardiac ischemia, nephropathy, peripheral artery occlusive disease (PAOD), and/or renal failure].

		All Samples^a^			Males			Females	
	No complications	At least one complication	At least two complications	No complications	At least one complication	At least two complications	No complications	At least one complication	At least two complications
	**N = 223**	**N = 243**	**N = 109**	**N = 111**	**N = 146**	**N = 69**	**N = 112**	**N = 97**	**N = 40**
**H:**	68 (30.49%)	93 (38.27%)	49 (44.95%)	34 (30.64%)	56 (38.38%)	29 (42.03%)	34 (30.36%)	37 (38.16%)	20 (50.00%)
H*	34 (15.25%)	43 (17.70%)	26 (23.85%)	18 (16.22%)	25 (17.12%)	14 (20.29%)	16 (14.29%)	18 (18.56%)	12 (30.00%)
**H1**	19 (8.52%)	25 (10.29%)	12 (11.01%)	9 (8.11%)	14 (9.59%)	8 (11.59%)	10 (8.93%)	11 (11.34%)	4 (10.00%)
**H3**	1 (0.45%)	9 (3.70%)	6 (5.50%)	···	6 (4.11%)	3 (4.35%)	1 (0.89%)	3 (3.09%)	3 (7.50%)
**H5**	8 (3.59%)	8 (3.29%)	3 (2.75%)	6 (5.41%)	5 (3.42%)	3 (4.35%)	2 (1.79%)	3 (3.09%)	···
**H6**	4 (1.79%)	6 (2.47%)	2 (1.83%)	1 (0.90%)	4 (2.74%)	1 (1.45%)	3 (2.68%)	2 (2.06%)	1 (2.50%)
**H9**	2 (0.90%)	2 (0.82%)	···	···	2 (1.37%)	···	2 (1.79%)	···	···
**HV:**	20 (8.97%)	17 (7.00%)	8 (7.34%)	11 (9.91%)	14 (9.59%)	8 (11.59%)	9 (8.04%)	3 (3.09%)	···
HV*	10 (4.48%)	5 (2.06%)	···	5 (4.50%)	3 (2.05%)	···	5 (4.46%)	2 (2.06%)	···
**HV0**	2 (0.90%)	2 (0.82%)	1 (0.92%)	2 (1.80%)	1 (0.68%)	1 (1.45%)	···	1 (1.03%)	···
**V**	8 (3.59%)	10 (4.12%)	7 (6.42%)	4 (3.60%)	10 (6.85%)	7 (10.14%)	4 (3.57%)	···	···
**R0:**	2 (0.90%)	4 (1.65%)	1 (0.92%)	···	3 (2.05%)	1 (1.45%)	2 (1.79%)	1 (1.03%)	···
**R0a**	2 (0.90%)	4 (1.65%)	1 (0.92%)	···	3 (2.05%)	1 (1.45%)	2 (1.79%)	1 (1.03%)	···
**J:**	17 (7.62%)	16 (6.58%)	5 (4.59%)	10 (9.01%)	11 (7.53%)	4 (5.80%)	7 (6.25%)	5 (5.15%)	1 (2.50%)
**J1**	13 (5.83%)	14 (5.76%)	5 (4.59%)	7 (6.31%)	11 (7.53%)	4 (5.80%)	6 (5.36%)	3 (3.09%)	1 (2.50%)
**J2**	4 (1.79%)	2 (0.82%)	···	3 (2.70%)	···	···	1 (0.89%)	2 (2.06%)	···
**T:**	41 (18.39%)	30 (12.35%)	13 (11.93%)	22 (19.82%)	15 (10.27%)	7 (10.14%)	19 (16.96%)	15 (15.46%)	6 (15.00%)
**T1**	6 (2.69%)	6 (2.47%)	3 (2.75%)	2 (1.80%)	5 (3.42%)	2 (2.90%)	4 (3.57%)	1 (1.03%)	1 (2.50%)
**T2**	35 (15.70%)	24 (9.88%)	10 (9.17%)	20 (18.02%)	10 (6.85%)	5 (7.25%)	15 (13.39%)	14 (14.43%)	5 (12.50%)
**UK:**									
**U**	36 (16.14%)	44 (18.10%)	19 (17.43%)	18 (16.22%)	30 (20.55%)	12 (17.39%)	18 (16.06%)	14 (14.43%)	7 (17.50%)
**U1**	1 (0.45%)	2 (0.82%)	1 (0.92%)	1 (0.90%)	2 (1.37%)	1 (1.45%)	···	···	···
**U2**	···	1 (0.41%)	···	···	1 (0.68%)	···	···	···	···
**U3**	6 (2.69%)	7 (2.88%)	6 (5.50%)	5 (4.50%)	5 (3.42%)	4 (5.80%)	1 (0.89%)	2 (2.06%)	2 (5.00%)
**U4**	7 (3.14%)	5 (2.06%)	1 (0.92%)	2 (1.80%)	4 (2.74%)	···	5 (4.46%)	1 (1.03%)	1 (2.50%)
**U5**	19 (8.52%)	20 (8.23%)	8 (7.34%)	8 (7.21%)	13 (8.90%)	5 (7.25%)	11 (9.82%)	7 (7.22%)	3 (7.50%)
**U6**	···	2 (0.82%)	···	···	···	···	···	2 (2.06%)	···
**U7**	2 (0.90%)	2 (0.82%)	···	1 (0.90%)	1 (0.68%)	···	1 (0.89%)	1 (1.03%)	···
**U8**	1 (0.45%)	4 (1.65%)	2 (1.83%)	1 (0.90%)	3 (2.05%)	1 (1.45%)	···	1 (1.03%)	1 (2.50%)
**U9**	···	1 (0.41%)	1 (0.92%)	···	1 (0.68%)	1 (1.45%)	···	···	···
**K**	12 (5.38%)	19 (7.82%)	4 (3.67%)	2 (1.80%)	10 (6.85%)	4 (5.80%)	10 (8.93%)	9 (9.28%)	···
**K1**	12 (5.38%)	18 (7.41%)	4 (3.67%)	2 (1.80%)	10 (6.85%)	4 (5.80%)	10 (8.93%)	8 (8.25%)	···
**K2**		1 (0.41%)	···	···	···	···	···	1 (1.03%)	···
**N1:**	7 (3.14%)	10 (4.12%)	5 (4.59%)	5 (4.50%)	4 (2.74%)	1 (1.45%)	2 (1.79%)	6 (6.19%)	4 (10.00%)
**I**	4 (1.79%)	5 (2.06%)	2 (1.83%)	4 (3.60%)	2 (1.37%)	···	···	3 (3.09%)	2 (5.00%)
**N1**	3 (1.35%)	5 (2.06%)	3 (2.75%)	1 (0.90%)	2 (1.37%)	1 (1.45%)	2 (1.79%)	3 (3.09%)	2 (5.00%)
**N2:**	4 (1.79%)	2 (0.82%)	1 (0.92%)	2 (1.80%)	1 (0.68%)	1 (1.45%)	2 (1.79%)	1 (1.03%)	···
**W**	4 (1.79%)	2 (0.82%)	1 (0.92%)	2 (1.80%)	1 (0.68%)	1 (1.45%)	2 (1.79%)	1 (1.03%)	···
**X:**	8 (3.59%)	5 (2.06%)	2 (1.83%)	3 (2.70%)	1 (0.68%)	1 (1.45%)	5 (4.46%)	4 (4.12%)	1 (2.50%)
**X2**	8 (3.59%)	5 (2.06%)	2 (1.83%)	3 (2.70%)	1 (0.68%)	1 (1.45%)	5 (4.46%)	4 (4.12%)	1 (2.50%)
**M:**	7 (3.14%)	3 (1.23%)	2 (1.83%)	4 (3.60%)	1 (0.68%)	1 (1.45%)	3 (2.68%)	2 (2.06%)	1 (2.50%)
**D4**	4 (1.79%)	1 (0.41%)	1 (0.92%)	3 (2.70%)	1 (0.68%)	1 (1.45%)	1 (0.89%)	…	···
**M1**	3 (1.35%)	2 (0.82%)	1 (0.92%)	1 (0.90%)	···	···	2 (1.79%)	2 (2.06%)	1 (2.50%)
**L:**	1 (0.45%)	···	···	···	···	···	1 (0.89%)	···	···
**L1b**	···	···	···	···	···	···	···	···	···
**L3**	1 (0.45%)	···	···	···	···	···	1 (0.89%)	···	···

a Haplogroup frequencies in males and females are reported for congruency with previous tables. However, gender was not considered in statistical analyses dealing with complications.

**Table 4:** Logistic-regression analyses of haplogroups associated with T2DM complications.

Modeled Outcome	Haplogroup	*p-value*	O.R.^b^	95% C.I.^c^
One complication (***Sig.*** a = 0.0090)				
	H3	0.0427	8.5385	1.0730–67.9460
Two complications (***Sig.*** = 0.0007)^§^				
	H*	0.0083	2.0682	1.2055–3.5481
	H3	0.0044	6.5625	1.7993–23.9349
	U3	0.0211	3.7500	1.2190–11.5361
	V	0.0418	2.7841	1.0389–7.4610

a *Significance (p-value)* relative to the final model considering all slopes  =  zero (no effect of the included I.V., taken together, on the outcome), obtained by the likelihood ratio test. The symbol § highlights significant values (α≤0.003).

b Odd ratio.

c Confidence interval.

**Table 5:** “Inverted” logistic-regression analyses employed to evaluate characteristics associated with candidate haplogroups.

Modeled Outcome a	Characteristics	*p-value*	O.R.	95% C.I.
H3 subjects (***Sig.*** b = 0.0007)^§^				
	Neuropathy	0.0012	9.6389	2.4411–38.0594
H c subjects (***Sig.*** = 0.0007)^§^				
	Retinopathy	0.0014	2.0075	1.3080–3.0812
	HDL	0.0259	0.9836	0.9694–0.9980
U3 subjects (***Sig.*** = 0.0247)				
	Nephropathy	0.0154	4.1518	1.3118–13.1401
V subjects (***Sig.*** = 0.0276)				
	Renal Failure	0.0103	5.8429	1.5159–22.5206

a The candidate haplogroup was analyzed in relation to the patients' traits and complications reported in Table 1.

b *Significance (p-value)* relative to the final model considering all slopes  =  zero (no effect of the included I.V., taken together, on the outcome), obtained by the likelihood ratio test. The symbol § highlights significant values (α≤0.003).

c H here represents the entire clade, thus including H*, H1, H3, H5, H6 and H9.

Finally, U3 and V subjects showed an increased occurrence of nephropathy (OR = 4.1518, 95% CI: 1.3118–13.1401, *p-value* 0.0154) and renal failure (OR = 5.8429, 95% CI: 1.5159–22.5206, *p-value* 0.0103), respectively (Table 5).

In order to compute confidence bounds around the predictions, we tested the significant haplogroups trough a decision tree analysis. Actually, all the reported associations were supported (Figure S1): H3 entered in the final decision tree when analyzing one T2DM complication, more than one complication and neuropathy (*p-values* 0.027, 0.034 and <0.001, respectively); while H, U3 and V were significant predictors for retinopathy (*p-value* 0.036), nephropathy (*p-value* 0.011) and renal failure (*p-value* 0.019), respectively.

## Discussion

**Figure 2:**
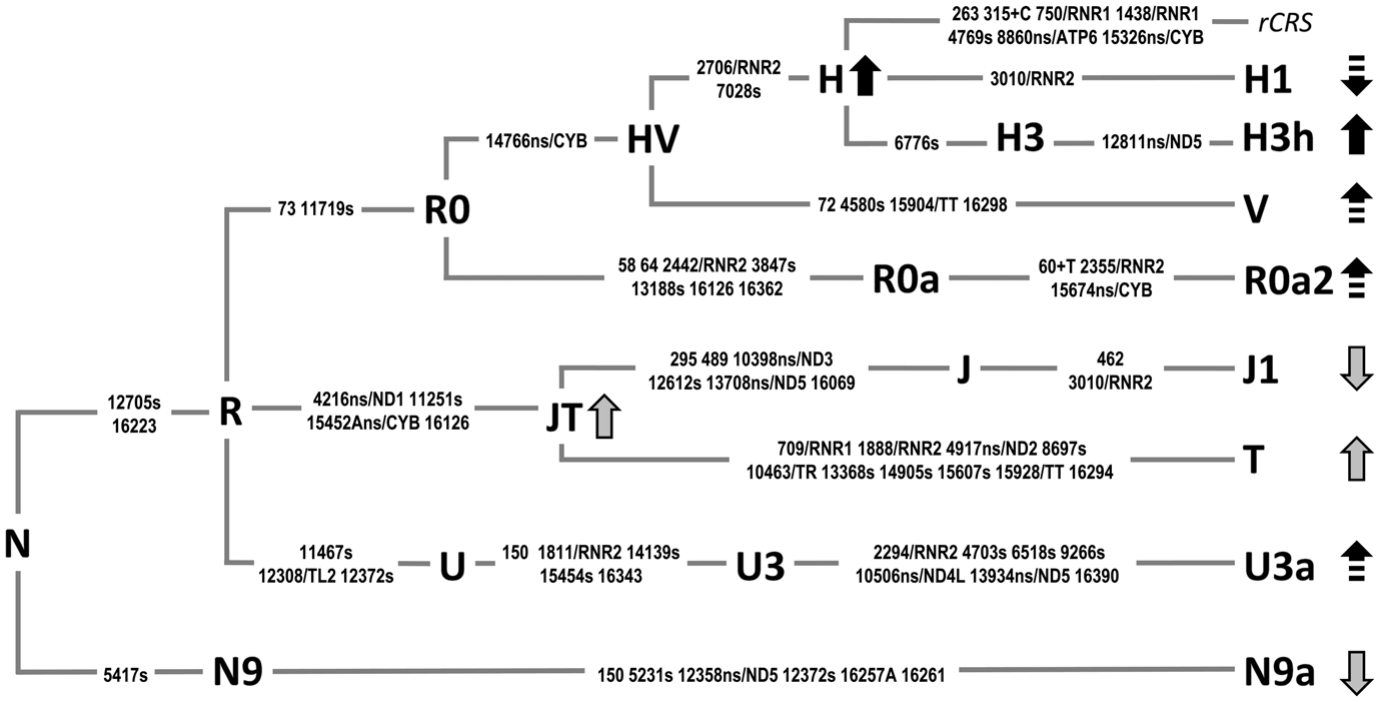
MtDNA tree encompassing the roots of haplogroups associated with T2DM and/or its complications. The distinguishing mutational motifs for the haplogroups shown in the tree are reported on the branches and they are transitions unless a base is explicitly indicated. The position of the rCRS [43] is indicated for reading off sequence motifs. Suffixes indicate transversions (to A), insertions (+), synonymous or non-synonymous changes (s or ns), gene locus (for tRNA, rRNA and non-synonymous mutations – following the nomenclature proposed by MITOMAP). A role for haplogroups R0a/R0a2, H, H1, H3/H3 h, V, and U3/U3a has been proposed in this study. The protective or pejorative haplogroup effect is indicated by down or up arrows. Continuous arrow lines mean highly significant values. Previous analyses found associations (gray arrows) with J1 [13], JT and T [14], and N9a [11], [12].

As a first step in this study, we examined the relationships between T2DM and a wide range of mtDNA haplogroups and sub-haplogroups in a large-scale association study carried out on an Italian regional population. A reduced susceptibility to diabetes was possibly detected only for the H1 mtDNA background (9.4% and 15.8% in patients and controls, respectively). This H sub-branch is common in Western Europe (∼22% in the Iberian Peninsula, ∼13.7% in France and ∼15.3% in Scandinavia) and North Africa (average frequency of ∼16%) [28], and probably marks the expansions from the Franco-Cantabrian refuge zone when climatic conditions improved after the Last Glacial Maximum [29]. As shown in the schematic tree of figure 2, which illustrates the basal mutational motifs of all haplogroups associated with T2DM and/or its complications until now, H1 differs from the root of H only for the G3010A transition in the MTRNR2 gene. It is important to note that the same nucleotide change, due to an independent mutational event, characterizes also haplogroup J1 (Figure 2), whose protective role in diabetes has been previously postulated [13]. It is also worth mentioning that polymorphic variations in the mtDNA rRNA genes have been proposed as modulators affecting the penetrance of some specific pathogenic mutations causing non-syndromic deafness and LHON [30], [31]. Taking into account that the entire haplogroup H is characterized by another base substitution in MTRNR2 (G2706A), it is conceivable that such a combination of polymorphisms in the same gene might modulate susceptibility to diabetes.

Similar to haplogroup H1, the rare R0a2 branch (Figure 2), which was found only among diabetic patients in our sample (Table 2 and Figure 1), is also characterized by a mutation (A2355G) in the MTRNR2 gene (Figures 2). This subclade of the rare R0a haplogroup – 0.9% in Italy [32] – harbors also the non-synonymous transition MTCYB-T15674C/S310P affecting an amino acid position with a very high conservation index (C.I. = 92.31, calculated using the mtPhyl program http://eltsov.org/mtphyl.aspx). Thus, such a molecular change might affect the biochemical efficiency of the respiratory chain complex III. Moreover, it should be also considered that all R02a mtDNAs harbor a T insertion at np 60 in the H-strand replication origin, a sequence stretch whose variation has been recently associated to an increase of mtDNA content in T2DM patients [33].

Unfortunately, neither the association with haplogroup H1 nor that with haplogroup R0a was statistically supported. In fact, the R0a mtDNAs were too few to be included in the logistic regression, and H1 did not reach the established level of significance after the Bonferroni correction (*p-value*>0.003). Thus, European mtDNA haplogroups, even when analyzed at a very high level of molecular and phylogenetic resolution, do not appear to play a major role in T2DM as a whole, at least in the context of a well defined population of central Italy, as that of the Marche region.

In contrast, when we evaluated the potential role of mtDNA backgrounds in complications rather than in T2DM as a whole, we were able to build a very significant logistic model (*p-value*<0.001). We observed that four mtDNA haplogroups (H, H3, U3 and V) were associated with an increased risk of complications, in particular with the risk of developing at least two common T2DM complications (Table 4). Intriguingly, we found that each haplogroup was related to a different pathology: U3 to nephropathy, V to renal failure, H to retinopathy, and H3 to neuropathy (Table 5).

The excess of U3 mtDNAs among nephropathic subjects (7.8% *vs.* 2.0% in T2DM controls, Table S5) is difficult to explain since this branch is characterized only by control-region mutations and synonymous coding-region transitions (Figure 2). A plausible explanation might dwell in the incidence of its subclade U3a that is almost 4-fold more represented in nephropathic cases (4.7% *vs.* 1.2%). The U3a mtDNAs share at least two non-synonymous mutations in two different subunits of the NADH dehydrogenase complex (MTND4L-A10506G/T13A, C.I. = 46.15; MTND5-T13934C/T533M, C.I. = 15.38).

As for haplogroup V, it was found to be associated with renal failure (15.0% in cases *vs.* 3.4% in T2DM controls, Table S7), which is a pejorative condition of nephropathy. Actually, the nephrological problems of the three identified V patients (Table S5) always ended with renal failure (Table S7). Their mtDNAs harbor the transition C15904T in the tRNA threonine gene that might accentuate the possible effect of the amino-acid change isoleucine to threonine (MTCYB-C14766T/I7L, C.I. = 48.72) distinctive of the entire superhaplogroup HV. The latter mutation, together with the MTRNR2-G2706A transition, could be also involved in the 2-fold increased risk of retinopathy for diabetic patients (44.7% in patients *vs.* 30.5% in T2DM controls, Table S2) belonging to H, the most common European haplogroup (Table 5). Within haplogroup H, it is difficult to provide an explanation for the pejorative role of H3 with regard to neuropathy. Indeed haplogroup H3 is defined only by the synonymous mutation MTCOI-T6776C (Figure 2). However, through a complete sequence analysis of H3 mtDNAs (data not shown), we were able to identify a new internal branch of H3 (named H3h in Figure 2) defined by the amino acid change MTND5-T12811C/Y53H (C.I. = 53.85). The incidence of this subgroup within H3 is much higher among the neuropathic patients (57%) than in controls (37%).

Overall, we observed that most of the candidate branches in the mtDNA tree are characterized by mutations in the MTRNR2 gene and amino acid changes affecting cytochrome b and subunits of the respiratory enzyme complex I (Figure 2). Actually, recent evidence of a stable mitochondrial supercomplex (I–III) [34]–[36] raises the possibility that amino acid changes in the two complexes might directly interact with each other, eventually increasing ROS production and in this way influencing the onset of diabetic complications involving neuronal tissues (neuropathy and retinopathy) [37] and nephronal structures (nephropathy and renal failure) [38], tissues that are highly susceptible to oxidative damage.

In conclusion, our data appear to indicate that mitochondrial backgrounds do not play a significant role in causing the onset of type 2 diabetes, despite indications of a protective effect for haplogroup H1 – possibly due to the G3010A transition in the MTRNR2 gene. As H1 is common in Western Europe, such a possibility might be further evaluated by assaying diabetic cohorts (and matched controls) of other European populations (see below). In contrast, we found significant associations between some European mtDNA haplogroups and typical diabetes complications. We cannot exclude that these associations might be influenced by nuclear genomic backgrounds and genetic substructure of the analyzed population, or biased by the reduced statistical power due to the decreased sample size of subgroups (patients with T2DM complications ascribed to different haplogroups) [39]. When we evaluated the latter scenario by calculating the power values for each haplogroup (Table 6) according to a previously described method [40], we observed, as would be expected due to the high haplogroup resolution of this study, rather low power values ranging from 4.4% to 42.8%. Taking into account the size of our samples (about 450 cases and 450 controls), even for haplogroup H - the most common in our study - we would be able to reach a 90% power value only if there was a frequency difference ≥40% in the T2DM group relative to the control group (41%). On the other hand, the finding that the highest power values were generally observed for the same haplogroups for which we found an association with T2DM (H1 and possibly R0a) and T2DM complications (H, H3, U3 and V) tends to support the scenario that these haplogroups do indeed play a role in T2DM complications. It should also be pointed out that our power analysis results raise the possibility that additional associations between mtDNA haplogroups and diabetes complications might exist and that they were not detected in our study simply because the analyzed cohorts were not large enough to have the power to identify small effects.

**Table 6:** Power values calculated for each haplogroup in different comparisons.

Haplogroup^a^	Power (%)^b^		
	T2DM Patients/Controls	At least one complication/No complications	At least two complications/One or no complications
**H1**	42.81	6.12	5.28
**H3**	6.60	31.46	18.30
**H5**	4.99	4.49	4.79
**H6**	6.07	5.43	4.53
**H** c	4.42	5.07	14.59
**HV** d	4.37	11.98	17.81
**V**	10.15	4.67	9.51
**R0a**	31.50	n.d.	n.d.
**J**	6.78	5.06	8.08
**T1**	4.40	4.44	4.44
**T2**	8.62	19.82	8.60
**U3**	4.67	20.66	11.77
**U5**	4.79	4.42	4.91
**U4/U9**	8.37	4.97	5.82
**U8b/K**	6.65	13.91	7.08
**N1**	12.86	5.69	5.13
**X2**	5.42	8.18	5.82

a These haplogroups (excluding R0a) correspond to those tested in the logistic regression models.

b Power percentages were calculated as reported in [40]. The number of cases (N_c_) was assumed different in each comparison, while the number of haplogroups (N_H_) was always set at 16. The underlined power values refer to haplogroups H1, R0a and the other haplogroups that were statistically significant in the logistic models (see Table 4).

c H here includes H*, H8 and H9 of Tables 2 and 3.

d HV here includes HV* and HV0 of Tables 2 and 3.

In brief, our study provides important clues indicating that certain mtDNA haplogroups might modulate diabetes complications. Obviously to definitively link mtDNA backgrounds with T2DM complications additional studies at the same level of phylogenetic resolution in other populations with similar haplogroup/subhaplogroup profiles are required. It is also likely that for many uncommon subhaplogroups only meta-analyses encompassing data from multiple studies will be able to reach power values that are adequate to provide definitive answers on the issue.

## Materials and Methods

### Ethics Statement

All experimental procedures and written informed consent, obtained from all donors, were reviewed and approved by the Ethics Committee of the National Institute on Health and Science on Aging (INRCA), Ancona, Italy, in accordance with the European Union Directive 86/609.

### Samples

A sample of 904 unrelated subjects (433 males and 471 females) age 40 years and older was collected by the Diabetology Unit, INRCA (National Institute on Health and Science on Aging) in Ancona (Italy). This included 466 patients affected with T2DM – whose diagnosis was made according to the American Diabetes Association Criteria (http://www.diabetes.org/) – and 438 control cases. The mean age for each group was 65.84±8.19 and 59.96±9.97, respectively. To avoid population stratification effects, only patients and controls with at least two generations of maternal ancestry from the Marche region (Central Italy) were included in this study. All five provinces of the region are represented: 794 from Ancona; 42 from Macerata; 15 from Ascoli Piceno; 3 from Pesaro and Urbino; 1 from Fermo; 49 of unspecified Marchigian descent.

The basic phenotypical and clinical characteristics (including data on vital signs, anthropometric factors, medical history, behavior and lifestyle, etc.) of the sample are summarized in Table 1. A predominantly Mediterranean diet was reported by all subjects. Controls did not show signs of illness and did not take any prescription drugs. The presence/absence of microvascular and macrovascular diabetic complications was evidenced as follows: microvascular: (1) diabetic retinopathy by fundoscopy through dilated pupils and/or fluorescence angiography, (2) incipient nephropathy, defined by an excessive urinary albumin excretion (>30 mg/24 h) and a normal creatinine clearance, (3) renal failure, detected as an estimated glomerular filtration rate >60 mL/min per 1.73 m^2^, and (4) neuropathy established by electromyography; macrovascular: (1) ischemic heart disease diagnosed by clinical history, and/or ischemic electrocardiographic alterations, and (2) peripheral artery occlusive diseases (PAOD) including atherosclerosis obliterans and cerebrovascular disease, established on the basis of history, physical examinations and Doppler velocimetry.

Hypertension was defined as a systolic blood pressure >140 mmHg and/or a diastolic blood pressure >90 mmHg. The values were measured while the subjects were sitting and confirmed at least three times. Overnight fasting venous blood samples from all subjects were collected from 8:00 to 9:00 a.m. The biological samples were either analyzed immediately or stored at −80°C for no more than ten days. Blood concentrations for HDL cholesterol, triglycerides, HbA1c, fasting insulin, fibrinogen, high-sensitivity C reactive protein (hsCRP), creatinine, urea nitrogen, and white blood cells count were measured by standard procedures.

### MtDNA analysis

Total DNA was extracted from peripheral blood using standard commercial kits (Qiamp DNA Blood Maxi Kit, Qiagen) and stored at −20°C. The mtDNA from the 904 subjects was first analyzed by sequencing ∼800 bp from the control region for each subject (at least from nucleotide position [np] 16024 to np 220), thus including the entire hypervariable segment [HVS]-I [nps 16024–16383] and part of the HVS-II [nps 57–372]. The GenBank accession numbers for the 904 mtDNA control-region sequences are JF716451-JF717354. This analysis was followed by a hierarchical survey of haplogroup and sub-haplogroup diagnostic markers in the coding region, which allowed the classification of mtDNAs into different haplogroups and sub-haplogroups [32]. Also some paragroups were evaluated, for example paragroups H* and HV* (Tables 2–3). A paragroup is a term used to indicate lineages within a haplogroup that are not defined by additional marker mutations either because the marker mutation(s) are absent, or as yet undiscovered, or simply because they were not evaluated in the molecular screening. They are generally represented by an asterisk placed after the name of the haplogroup. For instance, our paragroup H* contains all (rather numerous) H mtDNAs that did not cluster within any of the subclades of H defined in the course of this study (H1, H3, H5, H6, H8 and H9). Therefore, there is no specific marker(s) for H* in addition to those that define the entire haplogroup H but, when statistically evaluating H*, the potential role of the unknown markers within H* can be assayed, eliminating the possible confounding effects of H1, H3, H5, H6, H8 and H9.

Sequencing of entire mtDNA genomes (belonging to haplogroups R0a and H3) and phylogenetic analysis were performed as previously described [5].

### Statistics

Statistical analyses were performed using the SPSS statistical package. Quantitative clinical data were compared between patients with diabetes and control individuals by the unpaired Student's *t* test. Qualitative data were compared using the Fisher's exact test. Because multiple comparisons were made, the established statistical significance (α≤0.050) was Bonferroni corrected to α≤0.004.

Binary logistic regressions were used to determine, simultaneously across the whole sample, whether the susceptibility to develop T2DM or T2DM complications – represented by binary dependent variables (or outcomes) taking on values 0 and 1) – differed among haplogroups. This approach reduces the chance of type I error (false-positive result) and controls for differences in the frequency of key variables among the different groups. MtDNA haplogroups are phylogenetically related, but they are also defined by different clusters of haplotype-specific polymorphisms. Thus, the categorical variable "haplogroup" is converted into different dummy variables (or predictors, one for each haplogroup) and introduced separately into the regression equation. To avoid small sample sizes, some of the haplogroups were grouped following phylogenetic considerations, whenever possible. The threshold was established at >10 subjects across the whole patients’ group, in keeping with the “rule of thumb” whereby logistic regression should be performed only when the number of studied subjects is one order of magnitude greater than each parameter. Thus, the uncommon haplogroups H8 and H9 went into H (together with H*); HV0 was grouped with the sister paragroup HV*; U4 and U9 were clustered together; U8b, K1 and K2 were considered as U8b/K; R0a, W, the remaining U subclades (namely U1, U2, U6 and U7), and the African/Asian haplogroups were not included in the logistic computation. After this correction, 16 (haplogroup) classes were obtained. To find out how these combined predictors affect the outcome variable (T2DM or T2DM complications) we used a stepwise forward method with the likelihood ratio (LR) test employed for entering the terms (probability thresholds: entry 0.05, since we have modeled two outcomes i.e. T2DM and T2DM complications; removal 0.100): the initial model contained only the constant (ß0); then the program searched for the predictor which has the highest simple correlation with the outcome variable; if this significantly improved the model, it was retained; then the program searched for the predictor which has the second highest semi-partial correlation with the outcome; if this significantly improved the model, it was retained, and so on. The chi-squared significance of the obtained model was computed by calculating the difference between log-likelihood statistic (-2LL) of the final block and that of the first step. Since we have modeled 16 haplogroups only the model *p-values* less than 0.003 were considered statistically significant.

In order to verify the relationship between mitochondrial haplogroups and T2DM complications we applied a decision tree analysis. In particular, the significant groupings in the logistic analyses (i.e. H3, H, U3 and V) were tested as predictors by Chi-squared Automatic Interaction Detection (CHAID) [41]. The CHAID tree was built by splitting subsets of the space into two or more child nodes repeatedly. To determine the best split at any node the CHAID algorithm merges each pair of categories of the predictor variable until a non-significant pair is found with regard to target variables. The process is repeated recursively until one of the stopping rules is triggered. The CHAID algorithm incorporates a sequential merge and split procedure based on Chi-square test statistics. In growing the tree the convergence criteria for CHAID were: epsilon = 0.001 and 100 maximum iterations. For each node chi square tests are computed.

Power values for each haplogroup were calculated by following the procedure previously described by Samuels et al. [40].

## Supporting Information

Figure S1 **CHAID diagrams assessing the association between T2DM complications and candidate haplogroups.** Chi-squared Automatic Interaction Detector (CHAID) was used to develop decision-tree analyses for the evaluation of T2DM complications, using those haplogroups that were significant in logistic analyses (H3, H, U3 and V) as predictors. As shown on panels “a-c”, only H3 haplogroup entered in the decision tree when predicting the presence of one or more complications and specifically neuropathy. Panel “d” confirms that H haplogroup was the predictor of retinopathy, while panels “e–f” confirm U3 and V as predictors of nephropathy and renal failure, respectively.
(TIF)

Table S1 **Control-region haplotypes and haplogroup/sub-haplogroup classification of the 904 mtDNAs from the Marche region.**
(DOC)

Table S2 **Frequencies of mtDNA haplogroups and sub-haplogroups in diabetic patients also affected by retinopathy.**
(DOC)

Table S3 **Frequencies of mtDNA haplogroups and sub-haplogroups in diabetic patients also affected by somatic neuropathy.**
(DOC)

Table S4 **Frequencies of mtDNA haplogroups and sub-haplogroups in diabetic patients also affected by cardiac ischemia.**
(DOC)

Table S5 **Frequencies of mtDNA haplogroups and sub-haplogroups in diabetic patients also affected by nephropathy.**
(DOC)

Table S6 **Frequencies of mtDNA haplogroups and sub-haplogroups in diabetic patients also affected by Peripheral Artery Occlusive Disease (PAOD).**
(DOC)

Table S7 **Frequencies of mtDNA haplogroups and sub-haplogroups in diabetic patients also affected by renal failure.**
(DOC)
