# Supplementary figures and images for: Mitochondrial DNA Backgrounds Might Modulate Diabetes Complications Rather than T2DM as a Whole
Source: PLoS One. 2011 Jun 9;6(6):e21029. doi: 10.1371/journal.pone.0021029 (PMC3111471; doi:10.1371/journal.pone.0021029)

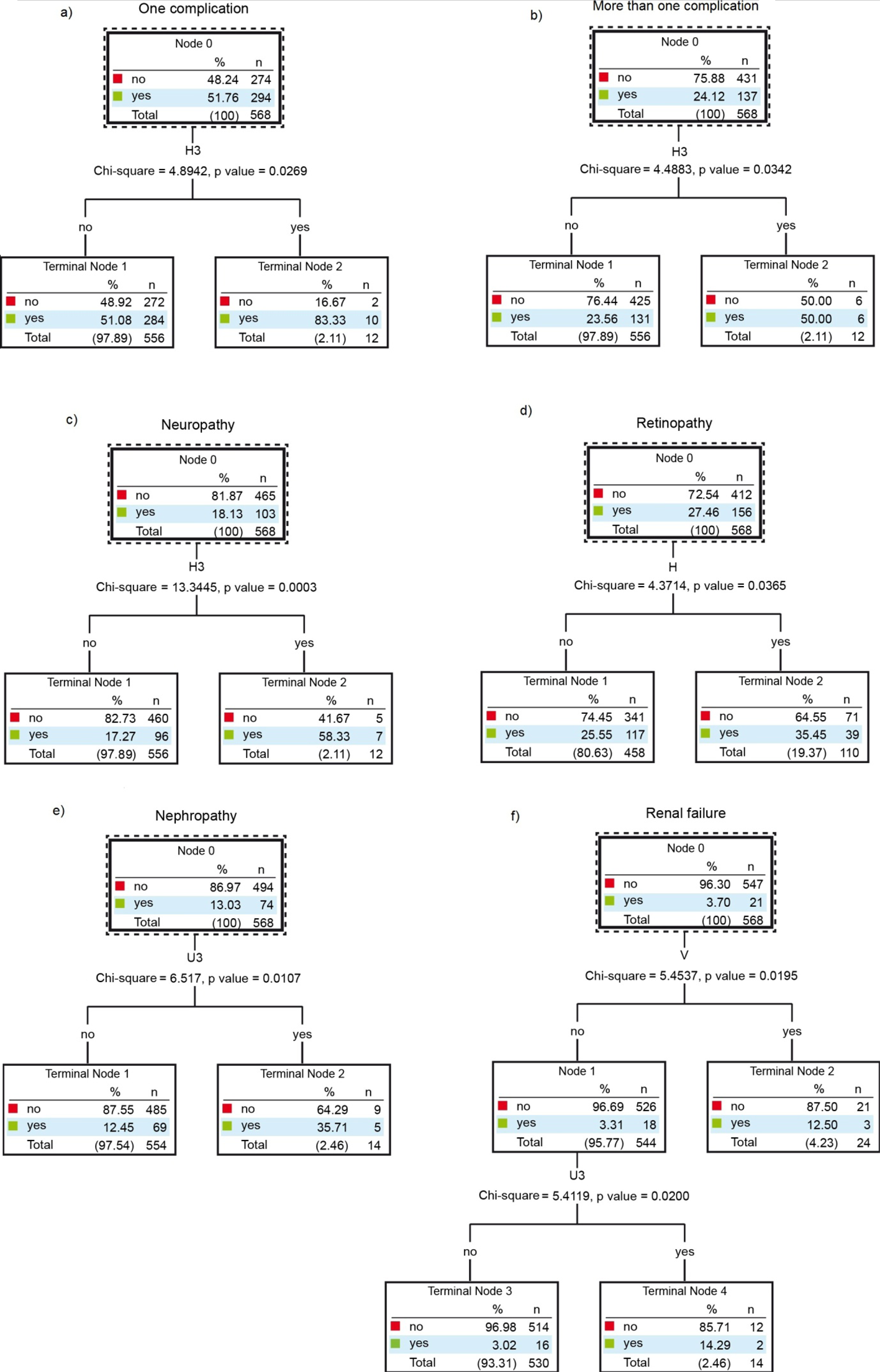

Supplement: Figure S1 — CHAID diagrams assessing the association between T2DM complications and candidate haplogroups. Chi-squared Automatic Interaction Detector (CHAID) was used to develop decision-tree analyses for the evaluation of T2DM complications, using those haplogroups that were significant in logistic analyses (H3, H, U3 and V) as predictors. As shown on panels “a-c”, only H3 haplogroup entered in the decision tree when predicting the presence of one or more complications and specifically neuropathy. Panel “d” confirms that H haplogroup was the predictor of retinopathy, while panels “e–f” confirm U3 and V as predictors of nephropathy and renal failure, respectively. (TIF) [file pone.0021029.s001.tif]
