# Supplementary material for: Mitochondrial DNA Backgrounds Might Modulate Diabetes Complications Rather than T2DM as a Whole
Source: PLoS One. 2011 Jun 9;6(6):e21029. doi: 10.1371/journal.pone.0021029 (PMC3111471; doi:10.1371/journal.pone.0021029)
Supplement: Table S2 — Frequencies of mtDNA haplogroups and sub-haplogroups in diabetic patients also affected by retinopathy. (DOC) [file pone.0021029.s003.doc]

**Table S2.** Frequencies of mtDNA haplogroups and sub-haplogroups in diabetic patients also affected by retinopathy.

| **Retinopathy** | **All samples** | | **Males** | | **Females** | |
| --- | --- | --- | --- | --- | --- | --- |
| **Haplogroup** | **Affected by Retinopathy (%)** | **Not Affected (%)** | **Affected by Retinopathy (%)** | **Not Affected (%)** | **Affected by Retinopathy (%)** | **Not Affected (%)** |
|  | **N=132** | **N=334** | **N=69** | **N=188** | **N=63** | **N=146** |
| **H:** | 59 (44.70%) | 102 (30.54%) | 33 (47.83%) | 57 (30.32%) | 26 (41.26%) | 45 (30.81%) |
| **H*** | 30 (22.73%) | 47 (14.07%) | 16 (23.19%) | 27 (14.36%) | 14 (22.22%) | 20 (13.70%) |
| **H1** | 16 (12.12%) | 28 (8.38%) | 10 (14.49%) | 13 (6.91%) | 6 (9.52%) | 15 (10.27%) |
| **H3** | 6 (4.55%) | 4 (1.20%) | 3 (4.35%) | 3 (1.60%) | 3 (4.76%) | 1 (0.68%) |
| **H5** | 3 (2.27%) | 13 (3.89%) | 1 (1.45%) | 10 (5.32%) | 2 (3.17%) | 3 (2.05%) |
| **H6** | 3 (2.27%) | 7 (2.10%) | 2 (2.90%) | 3 (1.60%) | 1 (1.59%) | 4 (2.74%) |
| **H8** | ... | ... | ... | ... | ... | ... |
| **H9** | 1 (0.76%) | 3 (0.90%) | 1 (1.45%) | 1 (0.53%) | ... | 2 (1.37%) |
| **HV:** | 7 (5.31%) | 30 (8.98%) | 5 (7.25%) | 20 (10.65%) | 2 (3.17%) | 10 (6.85%) |
| **HV*** | 1 (0.76%) | 14 (4.19%) | ... | 8 (4.26%) | 1 (1.59%) | 6 (4.11%) |
| **HV0** | 1 (0.76%) | 3 (0.90%) | ... | 3 (1.60%) | 1 (1.59%) | ... |
| **V** | 5 (3.79%) | 13 (3.89%) | 5 (7.25%) | 9 (4.79%) | ... | 4 (2.74%) |
| **R0:** | 1 (0.76%) | 5 (1.50%) | 1 (1.45%) | 2 (1.06%) | ... | 3 (2.05%) |
| **R0a** | 1 (0.76%) | 5 (1.50%) | 1 (1.45%) | 2 (1.06%) | ... | 3 (2.05%) |
| **J:** | 6 (4.55%) | 27 (8.09%) | 4 (5.80%) | 17 (9.05%) | 2 (3.17%) | 10 (6.85%) |
| **J1** | 6 (4.55%) | 21 (6.29%) | 4 (5.80%) | 14 (7.45%) | 2 (3.17%) | 7 (4.79%) |
| **J2** | ... | 6 (1.80%) | ... | 3 (1.60%) | ... | 3 (2.05%) |
| **T:** | 14 (10.60%) | 57 (17.06%) | 6 (8.70%) | 31 (16.49%) | 8 (12.70%) | 26 (17.81%) |
| **T1** | 3 (2.27%) | 9 (2.69%) | 2 (2.90%) | 5 (2.66%) | 1 (1.59%) | 4 (2.74%) |
| **T2** | 11 (8.33%) | 48 (14.37%) | 4 (5.80%) | 26 (13.83%) | 7 (11.11%) | 22 (15.07%) |
| **UK:** |  |  |  |  |  |  |
| **U** | 25 (18.96%) | 55 (16.46%) | 13 (18.85%) | 35 (18.61%) | 12 (19.05%) | 20 (13.70%) |
| **U1** | 2 (1.52%) | 1 (0.30%) | 2 (2.90%) | 1 (0.53%) | ... | ... |
| **U2** | 1 (0.76%) | ... | 1 (1.45%) | ... | ... | ... |
| **U3** | 2 (1.52%) | 11 (3.29%) | 1 (1.45%) | 9 (4.79%) | 1 (1.59%) | 2 (1.37%) |
| **U4** | 1 (0.76%) | 11 (3.29%) | ... | 6 (3.19%) | 1 (1.59%) | 5 (3.42%) |
| **U5** | 12 (9.09%) | 27 (8.08%) | 6 (8.70%) | 15 (7.98%) | 6 (9.52%) | 12 (8.22%) |
| **U6** | 2 (1.52%) | ... | ... | 0.00% | 2 (3.17%) | ... |
| **U7** | 1 (0.76%) | 3 (0.90%) | ... | 2 (1.06%) | 1 (1.59%) | 1 (0.68%) |
| **U8** | 3 (2.27%) | 2 (0.60%) | 2 (2.90%) | 2 (1.06%) | 1 (1.59%) | ... |
| **U9** | 1 (0.76%) | ... | 1 (1.45%) | ... | ... | ... |
| **K** | 10 (7.58%) | 21 (6.29%) | 5 (7.25%) | 7 (3.72%) | 5 (7.94%) | 14 (9.59%) |
| **K1** | 9 (6.82%) | 21 (6.29%) | 5 (7.25%) | 7 (3.72%) | 4 (6.35%) | 14 (9.59%) |
| **K2** | 1 (0.76%) | ... | ... | 0.00% | 1 (1.59%) | ... |
| **N1:** | 5 (3.79%) | 12 (3.60%) | 1 (1.45%) | 8 (4.26%) | 4 (6.35%) | 4 (2.74%) |
| **I** | 1 (0.76%) | 8 (2.40%) | ... | 6 (3.19%) | 1 (1.59%) | 2 (1.37%) |
| **N1** | 4 (3.03%) | 4 (1.20%) | 1 (1.45%) | 2 (1.06%) | 3 (4.76%) | 2 (1.37%) |
| **N2:** | 1 (0.76%) | ... | ... | ... | 1 (1.59%) | ... |
| **W** | 1 (0.76%) | 5 (1.50%) | ... | 3 (1.60%) | 1 (1.59%) | 2 (1.37%) |
| **X:** | 4 (3.03%) | 5 (1.50%) | 1 (1.45%) | 3 (1.60%) | 3 (4.76%) | 2 (1.37%) |
| **X2** | 4 (3.03%) | 9 (2.69%) | 1 (1.45%) | 3 (1.60%) | 3 (4.76%) | 6 (4.11%) |
| **M:** | ... | 10 (2.99%) | ... | 5 (2.66%) | ... | 5 (3.42%) |
| **D4** | ... | 5 (1.50%) | ... | 4 (2.13%) | ... | 1 (0.68%) |
| **M1** | ... | 5 (1.50%) | ... | 1 (0.53%) | ... | 4 (2.74%) |
| **L:** | ... | 1 (0.30%) | ... | ... | ... | 1 (0.68%) |
| **L1b** | ... | ... | ... | ... | ... | ... |
| **L3** | ... | 1 (0.30%) | ... | ... | ... | 1 (0.68%) |
