# Supplementary material for: Mitochondrial DNA Backgrounds Might Modulate Diabetes Complications Rather than T2DM as a Whole
Source: PLoS One. 2011 Jun 9;6(6):e21029. doi: 10.1371/journal.pone.0021029 (PMC3111471; doi:10.1371/journal.pone.0021029)
Supplement: Table S3 — Frequencies of mtDNA haplogroups and sub-haplogroups in diabetic patients also affected by somatic neuropathy. (DOC) [file pone.0021029.s004.doc]

**Table S3. Frequencies of mtDNA haplogroups and sub-haplogroups in diabetic patients also affected by somatic neuropathy.**

| **Somatic Neuropathy** | **All samples** | | **Males** | | **Females** | |
| --- | --- | --- | --- | --- | --- | --- |
| **Haplogroup** | **Affected by Somatic Neuropathy (%)** | **Not Affected (%)** | **Affected by Somatic Neuropathy (%)** | **Not Affected (%)** | **Affected by Somatic Neuropathy (%)** | **Not Affected (%)** |
|  | **N=94** | **N=372** | **N=64** | **N=193** | **N=30** | **N=179** |
| **H:** | 37 (39.38%) | 124 (33.34%) | 24 (37.51%) | 66 (34.20%) | 13 (43.34%) | 58 (32.40%) |
| **H*** | 18 (19.15%) | 59 (15.86%) | 10 (15.63%) | 33 (17.10%) | 8 (26.67%) | 26 (14.53%) |
| **H1** | 8 (8.51%) | 36 (9.68%) | 6 (9.38%) | 17 (8.81%) | 2 (6.67%) | 19 (10.61%) |
| **H3** | 7 (7.45%) | 3 (0.81%) | 5 (7.81%) | 1 (0.52%) | 2 (6.67%) | 2 (1.12%) |
| **H5** | 2 (2.13%) | 14 (3.76%) | 2 (3.13%) | 9 (4.66%) | ... | 5 (2.79%) |
| **H6** | 2 (2.13%) | 8 (2.15%) | 1 (1.56%) | 4 (2.07%) | 1 (3.33%) | 4 (2.23%) |
| **H8** | ... | ... | ... | ... | ... | ... |
| **H9** | ... | 4 (1.08%) | ... | 2 (1.04%) | ... | 2 (1.12%) |
| **HV:** | 8 (8.51%) | 29 (7.80%) | 8 (12.50%) | 17 (8.81%) | ... | 12 (6.70%) |
| **HV*** | 1 (1.06%) | 14 (3.76%) | 1 (1.56%) | 7 (3.63%) | ... | 7 (3.91%) |
| **HV0** | 1 (1.06%) | 3 (0.81%) | 1 (1.56%) | 2 (1.04%) | ... | 1 (0.56%) |
| **V** | 6 (6.38%) | 12 (3.23%) | 6 (9.38%) | 8 (4.15%) | ... | 4 (2.23%) |
| **R0:** | 1 (1.06%) | 5 (1.34%) | 1 (1.56%) | 2 (1.04%) | ... | 3 (1.68%) |
| **R0a** | 1 (1.06%) | 5 (1.34%) | 1 (1.56%) | 2 (1.04%) | ... | 3 (1.68%) |
| **J:** | 6 (6.38%) | 27 (7.25%) | 4 (6.25%) | 17 (8.81%) | 2 (6.67%) | 10 (5.59%) |
| **J1** | 5 (5.32%) | 22 (5.91%) | 4 (6.25%) | 14 (7.25%) | 1 (3.33%) | 8 (4.47%) |
| **J2** | 1 (1.06%) | 5 (1.34%) | ... | 3 (1.55%) | 1 (3.33%) | 2 (1.12%) |
| **T:** | 13 (13.83%) | 58 (15.59%) | 9 (14.06%) | 28 (14.51%) | 4 (13.33%) | 30 (16.76%) |
| **T1** | 4 (4.26%) | 8 (2.15%) | 3 (4.69%) | 4 (2.07%) | 1 (3.33%) | 4 (2.23%) |
| **T2** | 9 (9.57%) | 50 (13.44%) | 6 (9.38%) | 24 (12.44%) | 3 (10.00%) | 26 (14.53%) |
| **UK:** |  |  |  |  |  |  |
| **U** | 16 (17.02%) | 64 (17.22%) | 11 (17.19%) | 37 (19.17%) | 5 (16.67%) | 27 (15.08%) |
| **U1** | ... | 3 (0.81%) | ... | 3 (1.55%) | ... | ... |
| **U2** | ... | 1 (0.27%) | ... | 1 (0.52%) | ... | ... |
| **U3** | 4 (4.26%) | 9 (2.42%) | 2 (3.13%) | 8 (4.15%) | 2 (6.67%) | 1 (0.56%) |
| **U4** | 1 (1.06%) | 11 (2.96%) | 1 (1.56%) | 5 (2.59%) | ... | 6 (3.35%) |
| **U5** | 8 (8.51%) | 31 (8.33%) | 6 (9.38%) | 15 (7.77%) | 2 (6.67%) | 16 (8.94%) |
| **U6** | ... | 2 (0.54%) | ... | ... | ... | 2 (1.12%) |
| **U7** | ... | 4 (1.08%) | ... | 2 (1.04%) | ... | 2 (1.12%) |
| **U8** | 2 (2.13%) | 3 (0.81%) | 1 (1.56%) | 3 (1.55%) | 1 (3.33%) | ... |
| **U9** | 1 (1.06%) | ... | 1 (1.56%) | ... | ... | ... |
| **K** | 7 (7.45%) | 24 (6.45%) | 4 (6.25%) | 8 (4.15%) | 3 (10.00%) | 16 (8.94%) |
| **K1** | 7 (7.45%) | 23 (6.18%) | 4 (6.25%) | 8 (4.15%) | 3 (10.00%) | 15 (8.38%) |
| **K2** | ... | 1 (0.27%) | ... | ... | ... | 1 (0.56%) |
| **N1:** | 4 (4.26%) | 13 (3.49%) | 1 (1.56%) | 8 (4.15%) | 3 (10.00%) | 5 (2.79%) |
| **I** | 3 (3.19%) | 6 (1.61%) | 1 (1.56%) | 5 (2.59%) | 2 (6.67%) | 1 (0.56%) |
| **N1** | 1 (1.06%) | 7 (1.88%) | ... | 3 (1.55%) | 1 (3.33%) | 4 (2.23%) |
| **N2:** | 1 (1.06%) | 5 (1.34%) | 1 (1.56%) | 2 (1.04%) | ... | 3 (1.68%) |
| **W** | 1 (1.06%) | 5 (1.34%) | 1 (1.56%) | 2 (1.04%) | ... | 3 (1.68%) |
| **X:** | 1 (1.06%) | 12 (3.23%) | 1 (1.56%) | 3 (1.55%) | ... | 9 (5.03%) |
| **X2** | 1 (1.06%) | 12 (3.23%) | 1 (1.56%) | 3 (1.55%) | ... | 9 (5.03%) |
| **M:** | ... | 10 (2.69%) | ... | 5 (2.59%) | ... | 5 (2.79%) |
| **D4** | ... | 5 (1.34%) | ... | 4 (2.07%) | ... | 1 (0.56%) |
| **M1** | ... | 5 (1.34%) | ... | 1 (0.52%) | ... | 4 (2.23%) |
| **L:** | ... | 1 (0.27%) | ... | ... | ... | 1 (0.56%) |
| **L1b** | ... | ... | ... | ... | ... | ... |
| **L3** | ... | 1 (0.27%) | ... | ... | ... | 1 (0.56%) |
