# Supplementary material for: Mitochondrial DNA Backgrounds Might Modulate Diabetes Complications Rather than T2DM as a Whole
Source: PLoS One. 2011 Jun 9;6(6):e21029. doi: 10.1371/journal.pone.0021029 (PMC3111471; doi:10.1371/journal.pone.0021029)
Supplement: Table S4 — Frequencies of mtDNA haplogroups and sub-haplogroups in diabetic patients also affected by cardiac ischemia. (DOC) [file pone.0021029.s005.doc]

**Table S4. Frequencies of mtDNA haplogroups and sub-haplogroups in diabetic patients also affected by cardiac ischemia.**

| **Cardiac Ischemia** | **All samples** | | **Males** | | **Females** | |
| --- | --- | --- | --- | --- | --- | --- |
| **Haplogroup** | **Affected by Cardiac Ischemia (%)** | **Not Affected (%)** | **Affected by Cardiac Ischemia (%)** | **Not Affected (%)** | **Affected by Cardiac Ischemia (%)** | **Not Affected (%)** |
|  | **N=81** | **N=385** | **N=50** | **N=207** | **N=31** | **N=178** |
| **H:** | 25 (30.86%) | 136 (34.55%) | 13 (26.00%) | 77 (35.02%) | 12 (38.71%) | 59 (33.97%) |
| **H*** | 12 (14.81%) | 65 (16.52%) | 8 (16.00%) | 35 (16.73%) | 4 (12.90%) | 30 (16.27%) |
| **H1** | 7 (8.64%) | 37 (9.44%) | 3 (6.00%) | 20 (8.95%) | 4 (12.90%) | 17 (10.05%) |
| **H3** | 3 (3.70%) | 7 (2.15%) | 1 (2.00%) | 5 (2.33%) | 2 (6.45%) | 2 (1.91%) |
| **H5** | 2 (2.47%) | 14 (3.43%) | 1 (2.00%) | 10 (4.28%) | 1 (3.23%) | 4 (2.39%) |
| **H6** | 1 (1.23%) | 9 (2.15%) | ... | 5 (1.95%) | 1 (3.23%) | 4 (2.39%) |
| **H8** | ... | ... | ... | ... | ... | ... |
| **H9** | ... | 4 (0.86%) | ... | 2 (0.78%) | ... | 2 (0.96%) |
| **HV:** | 8 (9.88%) | 29 (7.94%) | 7 (14.00%) | 18 (9.73%) | 1 (3.23%) | 11 (5.74%) |
| **HV*** | 3 (3.70%) | 12 (3.22%) | 2 (4.00%) | 6 (3.11%) | 1 (3.23%) | 6 (3.35%) |
| **HV0** | ... | 4 (0.86%) | ... | 3 (1.17%) | ... | 1 (0.48%) |
| **V** | 5 (6.17%) | 13 (3.86%) | 5 (10.00%) | 9 (5.45%) | ... | 4 (1.91%) |
| **R0:** | 3 (3.70%) | 3 (1.29%) | 2 (4.00%) | 1 (1.17%) | 1 (3.23%) | 2 (1.44%) |
| **R0a** | 3 (3.70%) | 3 (1.29%) | 2 (4.00%) | 1 (1.17%) | 1 (3.23%) | 2 (1.44%) |
| **J:** | 5 (6.17%) | 28 (7.08%) | 4 (8.00%) | 17 (8.17%) | 1 (3.23%) | 11 (5.74%) |
| **J1** | 4 (4.94%) | 23 (5.79%) | 4 (8.00%) | 14 (7.00%) | ... | 9 (4.31%) |
| **J2** | 1 (1.23%) | 5 (1.29%) | ... | 3 (1.17%) | 1 (3.23%) | 2 (1.44%) |
| **T:** | 10 (12.35%) | 61 (15.24%) | 3 (6.00%) | 34 (14.40%) | 7 (22.58%) | 27 (16.27%) |
| **T1** | 1 (1.23%) | 11 (2.58%) | ... | 7 (2.72%) | 1 (3.23%) | 4 (2.39%) |
| **T2** | 9 (11.11%) | 50 (12.66%) | 3 (6.00%) | 27 (11.67%) | 6 (19.35%) | 23 (13.88%) |
| **UK:** |  |  |  |  |  |  |
| **U** | 18 (22.22%) | 62 (17.17%) | 13 (26.00%) | 35 (18.68%) | 5 (16.13%) | 27 (15.31%) |
| **U1** | ... | 3 (0.64%) | ... | 3 (1.17%) | ... | ... |
| **U2** | ... | 1 (0.21%) | ... | 1 (0.39%) | ... | ... |
| **U3** | 3 (3.70%) | 10 (2.79%) | 2 (4.00%) | 8 (3.89%) | 1 (3.23%) | 2 (1.44%) |
| **U4** | 4 (4.94%) | 8 (2.58%) | 3 (6.00%) | 3 (2.33%) | 1 (3.23%) | 5 (2.87%) |
| **U5** | 9 (11.11%) | 30 (8.37%) | 6 (12.00%) | 15 (8.17%) | 3 (9.68%) | 15 (8.61%) |
| **U6** | ... | 2 (0.43%) | ... | ... | ... | 2 (0.96%) |
| **U7** | 1 (1.23%) | 3 (0.86%) | 1 (2.00%) | 1 (0.78%) | ... | 2 (0.96%) |
| **U8** | 1 (1.23%) | 4 (1.07%) | 1 (2.00%) | 3 (1.56%) | ... | 1 (0.48%) |
| **U9** | ... | 1 (0.21%) | ... | 1 (0.39%) | ... | ... |
| **K** | 5 (6.17%) | 26 (6.65%) | 5 (10.00%) | 7 (4.67%) | ... | 19 (9.09%) |
| **K1** | 5 (6.17%) | 25 (6.44%) | 5 (10.00%) | 7 (4.67%) | ... | 18 (8.61%) |
| **K2** | ... | 1 (0.21%) | ... | ... | ... | 1 (0.48%) |
| **N1:** | 4 (4.94%) | 13 (3.38%) | 2 (4.00%) | 7 (3.38%) | 2 (6.45%) | 6 (3.37%) |
| **I** | 1 (1.23%) | 8 (1.93%) | ... | 6 (2.33%) | 1 (3.23%) | 2 (1.44%) |
| **N1** | 3 (3.70%) | 5 (1.72%) | 2 (4.00%) | 1 (1.17%) | 1 (3.23%) | 4 (2.39%) |
| **N2:** | 1 (1.23%) | 5 (1.29%) | 1 (2.00%) | 2 (1.17%) | ... | 3 (1.44%) |
| **W** | 1 (1.23%) | 5 (1.29%) | 1 (2.00%) | 2 (1.17%) | ... | 3 (1.44%) |
| **X:** | ... | 13 (2.79%) | ... | 4 (1.56%) | ... | 9 (4.31%) |
| **X2** | ... | 13 (2.79%) | ... | 4 (1.56%) | ... | 9 (4.31%) |
| **M:** | 2 (2.47%) | 8 (2.15%) | ... | 5 (1.95%) | 2 (6.45%) | 3 (2.39%) |
| **D4** | ... | 5 (1.07%) | ... | 4 (1.56%) | ... | 1 (0.48%) |
| **M1** | 2 (2.47%) | 3 (1.07%) | ... | 1 (0.39%) | 2 (6.45%) | 2 (1.91%) |
| **L:** | ... | 1 (0.21%) | ... | ... | ... | 1 (0.48%) |
| **L1b** | ... | ... | ... | ... | ... | ... |
| **L3** | ... | 1 (0.21%) | ... | ... | ... | 1 (0.48%) |
