# Supplementary material for: Mitochondrial DNA Backgrounds Might Modulate Diabetes Complications Rather than T2DM as a Whole
Source: PLoS One. 2011 Jun 9;6(6):e21029. doi: 10.1371/journal.pone.0021029 (PMC3111471; doi:10.1371/journal.pone.0021029)
Supplement: Table S5 — Frequencies of mtDNA haplogroups and sub-haplogroups in diabetic patients also affected by nephropathy. (DOC) [file pone.0021029.s006.doc]

**Table S5. Frequencies of mtDNA haplogroups and sub-haplogroups in diabetic patients also affected by nephropathy.**

| **Nefropathy** | **All samples** | | **Males** | | **Females** | |
| --- | --- | --- | --- | --- | --- | --- |
| **Haplogroup** | **Affected by Nefropathy (%)** | **Not Affected (%)** | **Affected by Nefropathy (%)** | **Not Affected (%)** | **Affected by Nefropathy (%)** | **Not Affected (%)** |
|  | **N=64** | **N=402** | **N=45** | **N=212** | **N=19** | **N=190** |
| **H:** | 27 (42.19%) | 134 (33.33%) | 17 (37.78%) | 73 (34.43%) | 10 (52.63%) | 61 (32.11%) |
| **H*** | 14 (21.88%) | 63 (15.67%) | 8 (17.78%) | 35 (16.51%) | 6 (31.58%) | 28 (14.74%) |
| **H1** | 8 (12.50%) | 36 (8.96%) | 4 (8.89%) | 19 (8.96%) | 4 (21.05%) | 17 (8.95%) |
| **H3** | 1 (1.56%) | 9 (2.24%) | 1 (2.22%) | 5 (2.36%) | ... | 4 (2.11%) |
| **H5** | 2 (3.13%) | 14 (3.48%) | 2 (4.44%) | 9 (4.25%) | ... | 5 (2.63%) |
| **H6** | 2 (3.13%) | 8 (1.99%) | 2 (4.44%) | 3 (1.42%) | ... | 5 (2.63%) |
| **H8** | ... | ... | ... | ... | ... | ... |
| **H9** | ... | 4 (1.00%) | ... | 2 (0.94%) | ... | 2 (1.05%) |
| **HV:** | 4 (6.25%) | 33 (8.21%) | 4 (8.89%) | 21 (9.91%) | ... | 12 (6.32%) |
| **HV*** | ... | 15 (3.73%) | ... | 8 (3.77%) | ... | 7 (3.68%) |
| **HV0** | 1 (1.56%) | 3 (0.75%) | 1 (2.22%) | 2 (0.94%) | ... | 1 (0.53%) |
| **V** | 3 (4.69%) | 15 (3.73%) | 3 (6.67%) | 11 (5.19%) | ... | 4 (2.11%) |
| **R0:** | 1 (1.56%) | 5 (1.24%) | 1 (2.22%) | 2 (0.94%) | ... | 3 (1.58%) |
| **R0a** | 1 (1.56%) | 5 (1.24%) | 1 (2.22%) | 2 (0.94%) | ... | 3 (1.58%) |
| **J:** | 4 (6.25%) | 29 (7.21%) | 4 (8.89%) | 17 (8.02%) | ... | 12 (6.32%) |
| **J1** | 4 (6.25%) | 23 (5.72%) | 4 (8.89%) | 14 (6.60%) | ... | 9 (4.74%) |
| **J2** | ... | 6 (1.49%) | ... | 3 (1.42%) | ... | 3 (1.58%) |
| **T:** | 10 (15.63%) | 61 (15.17%) | 5 (11.11%) | 32 (15.09%) | 5 (26.32%) | 29 (15.26%) |
| **T1** | 2 (3.13%) | 10 (2.49%) | 2 (4.44%) | 5 (2.36%) | ... | 5 (2.63%) |
| **T2** | 8 (12.50%) | 51 (12.69%) | 3 (6.67%) | 27 (12.74%) | 5 (26.32%) | 24 (12.63%) |
| **UK:** |  |  |  |  |  |  |
| **U** | 11 (17.19%) | 69 (17.16%) | 8 (17.78%) | 40 (18.87%) | 3 (15.79%) | 29 (15.26%) |
| **U1** | 1 (1.56%) | 2 (0.50%) | 1 (2.22%) | 2 (0.94%) | ... |  |
| **U2** | ... | 1 (0.25%) | ... | 1 (0.47%) | ... |  |
| **U3** | 5 (7.81%) | 8 (1.99%) | 3 (6.67%) | 7 (3.30%) | 2 (10.53%) | 1 (0.53%) |
| **U4** | ... | 12 (2.99%) | ... | 6 (2.83%) | ... | 6 (3.16%) |
| **U5** | 3 (4.69%) | 36 (8.96%) | 3 (6.67%) | 18 (8.49%) | ... | 18 (9.47%) |
| **U6** | ... | 2 (0.50%) | ... | ... | ... | 2 (1.05%) |
| **U7** | ... | 4 (1.00%) | ... | 2 (0.94%) | ... | 2 (1.05%) |
| **U8** | 2 (3.13%) | 3 (0.75%) | 1 (2.22%) | 3 (1.42%) | 1 (5.26%) | ... |
| **U9** | ... | 1 (0.25%) | ... | 1 (0.47%) | ... | ... |
| **K** | 3 (4.69%) | 28 (6.97%) | 3 (6.67%) | 9 (4.25%) | ... | 19 (10.00%) |
| **K1** | 3 (4.69%) | 27 (6.72%) | 3 (6.67%) | 9 (4.25%) | ... | 18 (9.47%) |
| **K2** | ... | 1 (0.25%) | ... | ... | ... | 1 (0.53%) |
| **N1:** | 2 (3.13%) | 15 (3.73%) | 1 (2.22%) | 8 (3.77%) | 1 (5.26%) | 7 (3.68%) |
| **I** | 1 (1.56%) | 8 (1.99%) | ... | 6 (2.83%) | 1 (5.26%) | 2 (1.05%) |
| **N1** | 1 (1.56%) | 7 (1.74%) | 1 (2.22%) | 2 (0.94%) | ... | 5 (2.63%) |
| **N2:** | 1 (1.56%) | 5 (1.24%) | 1 (2.22%) | 2 (0.94%) | ... | 3 (1.58%) |
| **W** | 1 (1.56%) | 5 (1.24%) | 1 (2.22%) | 2 (0.94%) | ... | 3 (1.58%) |
| **X:** | ... | 13 (3.23%) | ... | 4 (1.89%) | ... | 9 (4.74%) |
| **X2** | ... | 13 (3.23%) | ... | 4 (1.89%) | ... | 9 (4.74%) |
| **M:** | 1 (1.56%) | 9 (2.24%) | 1 (2.22%) | 4 (1.89%) | ... | 5 (2.63%) |
| **D4** | 1 (1.56%) | 4 (1.00%) | 1 (2.22%) | 3 (1.42%) | ... | 1 (0.53%) |
| **M1** | ... | 5 (1.24%) | ... | 1 (0.47%) | ... | 4 (2.11%) |
| **L:** | ... | 1 (0.25%) | ... | ... | ... | 1 (0.53%) |
| **L1b** | ... | ... | ... | ... | ... | ... |
| **L3** | ... | 1 (0.25%) | ... | ... | ... | 1 (0.53%) |
