# Supplementary material for: Mitochondrial DNA Backgrounds Might Modulate Diabetes Complications Rather than T2DM as a Whole
Source: PLoS One. 2011 Jun 9;6(6):e21029. doi: 10.1371/journal.pone.0021029 (PMC3111471; doi:10.1371/journal.pone.0021029)
Supplement: Table S6 — Frequencies of mtDNA haplogroups and sub-haplogroups in diabetic patients also affected by Peripheral Artery Occlusive Disease (PAOD). (DOC) [file pone.0021029.s007.doc]

**Table S6. Frequencies of mtDNA haplogroups and sub-haplogroups in diabetic patients also affected by Peripheral Artery Occlusive Disease (PAOD).**

| **Peripheral Artery Occlusive Disease (PAOD)** | **All samples** | | **Males** | | **Females** | |
| --- | --- | --- | --- | --- | --- | --- |
| **Haplogroup** | **Affected by AOCAI (%)** | **Not Affected (%)** | **Affected by AOCAI (%)** | **Not Affected (%)** | **Affected by AOCAI (%)** | **Not Affected (%)** |
|  | **N=30** | **N=436** | **N=17** | **N=240** | **N=13** | **N=196** |
| **H:** | 13 (43.33%) | 148 (33.94%) | 8 (47.06%) | 82 (34.17%) | 5 (38.46%) | 66 (33.67%) |
| **H*** | 6 (20.00%) | 71 (16.28%) | 4 (23.53%) | 39 (16.25%) | 2 (15.38%) | 32 (16.33%) |
| **H1** | 5 (16.67%) | 39 (8.94%) | 2 (11.76%) | 21 (8.75%) | 3 (23.08%) | 18 (9.18%) |
| **H3** | ... | 10 (2.29%) | ... | 6 (2.50%) | ... | 4 (2.04%) |
| **H5** | 1 (3.33%) | 15 (3.44%) | 1 (5.88%) | 10 (4.17%) | ... | 5 (2.55%) |
| **H6** | ... | 10 (2.29%) | ... | 5 (2.08%) | ... | 5 (2.55%) |
| **H8** | ... | ... | ... | ... | ... | ... |
| **H9** | 1 (3.33%) | 3 (0.69%) | 1 (5.88%) | 1 (0.42%) | ... | 2 (1.02%) |
| **HV:** | 2 (6.67%) | 35 (8.03%) | 2 (11.76%) | 23 (9.58%) | ... | 12 (6.12%) |
| **HV*** | ... | 15 (3.44%) | ... | 8 (3.33%) | ... | 7 (3.57%) |
| **HV0** | ... | 4 (0.92%) | ... | 3 (1.25%) | ... | 1 (0.51%) |
| **V** | 2 (6.67%) | 16 (3.67%) | 2 (11.76%) | 12 (5.00%) | ... | 4 (2.04%) |
| **R0:** | ... | 6 (1.38%) | ... | 3 (1.25%) | ... | 3 (1.53%) |
| **R0a** | ... | 6 (1.38%) | ... | 3 (1.25%) | ... | 3 (1.53%) |
| **J:** | 2 (6.67%) | 31 (7.11%) | 1 (5.88%) | 20 (8.33%) | 1 (7.69%) | 11 (5.61%) |
| **J1** | 2 (6.67%) | 25 (5.73%) | 1 (5.88%) | 17 (7.08%) | 1 (7.69%) | 8 (4.08%) |
| **J2** | ... | 6 (1.38%) | ... | 3 (1.25%) | ... | 3 (1.53%) |
| **T:** | 4 (13.33%) | 67 (15.37%) | 2 (11.76%) | 35 (14.58%) | 2 (15.38%) | 32 (16.33%) |
| **T1** | 3 (10.00%) | 9 (2.06%) | 2 (11.76%) | 5 (2.08%) | 1 (7.69%) | 4 (2.04%) |
| **T2** | 1 (3.33%) | 58 (13.30%) | ... | 30 (12.50%) | 1 (7.69%) | 28 (14.29%) |
| **UK:** |  |  |  |  |  |  |
| **U** | 3 (10.00%) | 77 (17.66%) | 2 (11.76%) | 46 (19.17%) | 1 (7.69%) | 31 (15.82%) |
| **U1** | ... | 3 (0.69%) | ... | 3 (1.25%) | ... | 0.00% |
| **U2** | ... | 1 (0.23%) | ... | 1 (0.42%) | ... | 0.00% |
| **U3** | ... | 13 (2.98%) | ... | 10 (4.17%) | ... | 3 (1.53%) |
| **U4** | ... | 12 (2.75%) | ... | 6 (2.50%) | ... | 6 (3.06%) |
| **U5** | 2 (6.67%) | 37 (8.49%) | 1 (5.88%) | 20 (8.33%) | 1 (7.69%) | 17 (8.67%) |
| **U6** | ... | 2 (0.46%) | ... | 0.00% | ... | 2 (1.02%) |
| **U7** | ... | 4 (0.92%) | ... | 2 (0.83%) | ... | 2 (1.02%) |
| **U8** | 1 (3.33%) | 4 (0.92%) | 1 (5.88%) | 3 (1.25%) | ... | 1 (0.51%) |
| **U9** | ... | 1 (0.23%) | ... | 1 (0.42%) | ... | 0.00% |
| **K** | 1 (3.33%) | 30 (6.88%) | ... | 12 (5.00%) | 1 (7.69%) | 18 (9.18%) |
| **K1** | 1 (3.33%) | 29 (6.65%) | ... | 12 (5.00%) | 1 (7.69%) | 17 (8.67%) |
| **K2** | ... | 1 (0.23%) | ... | ... | ... | 1 (0.51%) |
| **N1:** | 2 (6.67%) | 15 (3.44%) | 2 (11.76%) | 7 (2.92%) | ... | 8 (4.08%) |
| **I** | 1 (3.33%) | 8 (1.83%) | 1 (5.88%) | 5 (2.08%) | ... | 3 (1.53%) |
| **N1** | 1 (3.33%) | 7 (1.61%) | 1 (5.88%) | 2 (0.83%) | ... | 5 (2.55%) |
| **N2:** | ... | 6 (1.38%) | ... | 3 (1.25%) | ... | 3 (1.53%) |
| **W** | ... | 6 (1.38%) | ... | 3 (1.25%) | ... | 3 (1.53%) |
| **X:** | 2 (6.67%) | 11 (2.52%) | ... | 4 (1.67%) | 2 (15.38%) | 7 (3.57%) |
| **X2** | 2 (6.67%) | 11 (2.52%) | ... | 4 (1.67%) | 2 (15.38%) | 7 (3.57%) |
| **M:** | 1 (3.33%) | 9 (2.06%) | ... | 5 (2.08%) | 1 (7.69%) | 4 (2.04%) |
| **D4** | ... | 5 (1.15%) | ... | 4 (1.67%) | ... | 1 (0.51%) |
| **M1** | 1 (3.33%) | 4 (0.92%) | ... | 1 (0.42%) | 1 (7.69%) | 3 (1.53%) |
| **L:** | ... | 1 (0.23%) | ... | ... | ... | 1 (0.51%) |
| **L1b** | ... | ... | ... | ... | ... | ... |
| **L3** | ... | 1 (0.23%) | ... | ... | ... | 1 (0.51%) |
