# Supplementary material for: Mitochondrial DNA Backgrounds Might Modulate Diabetes Complications Rather than T2DM as a Whole
Source: PLoS One. 2011 Jun 9;6(6):e21029. doi: 10.1371/journal.pone.0021029 (PMC3111471; doi:10.1371/journal.pone.0021029)
Supplement: Table S7 — Frequencies of mtDNA haplogroups and sub-haplogroups in diabetic patients also affected by renal failure. (DOC) [file pone.0021029.s008.doc]

**Table S7. Frequencies of mtDNA haplogroups and sub-haplogroups in diabetic patients also affected by renal failure.**

| **Renal Failure** | **All samples** | | **Males** | | **Females** | |
| --- | --- | --- | --- | --- | --- | --- |
| **Haplogroup** | **Affected by Renal Failure (%)** | **Not Affected (%)** | **Affected by Renal Failure (%)** | **Not Affected (%)** | **Affected by Renal Failure (%)** | **Not Affected (%)** |
|  | **N=20** | **N=446** | **N=15** | **N=242** | **N=5** | **N=204** |
| **H:** | 7 (35.00%) | 154 (34.53%) | 5 (33.33%) | 85 (35.12%) | 2 (40.00%) | 69 (33.82%) |
| **H*** | 4 (20.00%) | 73 (16.37%) | 3 (20.00%) | 40 (16.53%) | 1 (20.00%) | 33 (16.18%) |
| **H1** | 2 (10.00%) | 42 (9.42%) | 1 (6.67%) | 22 (9.09%) | 1 (20.00%) | 20 (9.80%) |
| **H3** | ... | 10 (2.24%) | ... | 6 (2.48%) | ... | 4 (1.96%) |
| **H5** | 1 (5.00%) | 15 (3.36%) | 1 (6.67%) | 10 (4.13%) | ... | 5 (2.45%) |
| **H6** | ... | 10 (2.24%) | ... | 5 (2.07%) | ... | 5 (2.45%) |
| **H8** |  |  |  |  |  |  |
| **H9** | ... | 4 (0.90%) | ... | 2 (0.83%) | ... | 2 (0.98%) |
| **HV:** | 3 (15.00%) | 34 (7.62%) | 3 (20.00%) | 22 (9.09%) | ... | 12 (5.88%) |
| **HV*** | ... | 15 (3.36%) | ... | 8 (3.31%) | ... | 7 (3.43%) |
| **HV0** | ... | 4 (0.90%) | ... | 3 (1.24%) | ... | 1 (0.49%) |
| **V** | 3 (15.00%) | 15 (3.36%) | 3 (20.00%) | 11 (4.55%) | ... | 4 (1.96%) |
| **R0:** | ... | 6 (1.35%) | ... | 3 (1.24%) | ... | 3 (1.47%) |
| **R0a** | ... | 6 (1.35%) | ... | 3 (1.24%) | ... | 3 (1.47%) |
| **J:** | 2 (10.00%) | 31 (6.95%) | 2 (13.33%) | 19 (7.85%) | ... | 12 (5.88%) |
| **J1** | 2 (10.00%) | 25 (5.61%) | 2 (13.33%) | 16 (6.61%) | ... | 9 (4.41%) |
| **J2** | ... | 6 (1.35%) | ... | 3 (1.24%) | ... | 3 (1.47%) |
| **T:** | 2 (10.00%) | 69 (15.47%) | 1 (6.67%) | 36 (14.88%) | 1 (20.00%) | 33 (16.18%) |
| **T1** | ... | 12 (2.69%) | ... | 7 (2.89%) | ... | 5 (2.45%) |
| **T2** | 2 (10.00%) | 57 (12.78%) | 1 (6.67%) | 29 (11.98%) | 1 (20.00%) | 28 (13.73%) |
| **UK:** |  |  |  |  |  |  |
| **U** | 4 (20.00%) | 76 (17.04%) | 2 (13.33%) | 46 (19.01%) | 2 (40.00%) | 30 (14.71%) |
| **U1** | ... | 3 (0.67%) | ... | 3 (1.24%) | ... | ... |
| **U2** | ... | 1 (0.22%) | ... | 1 (0.41%) | ... | ... |
| **U3** | 2 (10.00%) | 11 (2.47%) | 1 (6.67%) | 9 (3.72%) | 1 (20.00%) | 2 (0.98%) |
| **U4** | ... | 12 (2.69%) | ... | 6 (2.48%) | ... | 6 (2.94%) |
| **U5** | 1 (5.00%) | 38 (8.52%) | 1 (6.67%) | 20 (8.26%) | ... | 18 (8.82%) |
| **U6** | ... | 2 (0.45%) | ... | 0.00% | ... | 2 (0.98%) |
| **U7** | ... | 4 (0.90%) | ... | 2 (0.83%) | ... | 2 (0.98%) |
| **U8** | 1 (5.00%) | 4 (0.90%) | ... | 4 (1.65%) | 1 (20.00%) | ... |
| **U9** | ... | 1 (0.22%) | ... | 1 (0.41%) | ... | ... |
| **K** | ... | 31 (6.95%) | ... | 12 (4.96%) | ... | 19 (9.31%) |
| **K1** | ... | 30 (6.73%) | ... | 12 (4.96%) | ... | 18 (8.82%) |
| **K2** | ... | 1 (0.22%) | ... | 0.00% | ... | 1 (0.49%) |
| **N1:** | 1 (5.00%) | 16 (3.59%) | 1 (6.67%) | 8 (3.31%) | ... | 8 (3.92%) |
| **I** | ... | 9 (2.02%) | ... | 6 (2.48%) | ... | 3 (1.47%) |
| **N1** | 1 (5.00%) | 7 (1.57%) | 1 (6.67%) | 2 (0.83%) | ... | 5 (2.45%) |
| **N2:** | ... | 6 (1.35%) | ... | 3 (1.24%) | ... | 3 (1.47%) |
| **W** | ... | 6 (1.35%) | ... | 3 (1.24%) | ... | 3 (1.47%) |
| **X:** | ... | 13 (2.91%) | ... | 4 (1.65%) | ... | 9 (4.41%) |
| **X2** | ... | 13 (2.91%) | ... | 4 (1.65%) | ... | 9 (4.41%) |
| **M:** | 1 (5.00%) | 9 (2.02%) | 1 (6.67%) | 4 (1.65%) | ... | 5 (2.45%) |
| **D4** | 1 (5.00%) | 4 (0.90%) | 1 (6.67%) | 3 (1.24%) | ... | 1 (0.49%) |
| **M1** | ... | 5 (1.12%) | ... | 1 (0.41%) | ... | 4 (1.96%) |
| **L:** | ... | 1 (0.22%) | ... | ... | ... | 1 (0.49%) |
| **L1b** |  |  |  |  |  |  |
| **L3** | ... | 1 (0.22%) | ... | ... | ... | 1 (0.49%) |
