# Supplementary material for: Mitochondrial DNA Backgrounds Might Modulate Diabetes Complications Rather than T2DM as a Whole
Source: PLoS One. 2011 Jun 9;6(6):e21029. doi: 10.1371/journal.pone.0021029 (PMC3111471; doi:10.1371/journal.pone.0021029)
Supplement: Supplementary file 8 [file pone.0021029.s002.doc]

## Sheet: Table S1

Table S1. Control-region haplotypes and haplogroup/sub-haplogroup classification of the 904 mtDNAs from the Marche region.
Province	Sample Name	GenBank       Acc. Numb.	Sub-Hg	Hg	Super-Hg	till 16569 (+16000)	till 220	T2DM	T2DM Complications
Ancona	AAA010D	JF716451	T1a	T1	T	126 186 189 248 266 271 294 311 519	73 152 	1
Ancona	AAA020D	JF716452	U5a	U5	U	192 256 270 526	73	1	Retinopathy, Cardiac Ischemia
Ancona	AAA030D	JF716453	H5	H5	H	086 214 304	146	0
Ancona	AAA040D	JF716454	T2c	T2	T	126 292 294 296 380 519	73 146 	1	Retinopathy, Somatic Neuropathy
Ancona	AAA050D	JF716455	K1	K1	K	224 311 519	73 199 	0
Ancona	AAA060D	JF716456	T1a	T1	T	126 163 176 186 189 294 311 519	73 146 150 152 	1	Retinopathy, Somatic Neuropathy, Peripheral Artery Occlusive Disease
Ancona	AAA070D	JF716457	J1b	J1	J	069 126 145 172 192 222 261 	73 146	1
Ancona	AAA080D	JF716458	T2c	T2	T	126 292 294 296 380 519	73 146 	1
Ancona	AAA090D	JF716459	T1a	T1	T	126 163 186 189 294 519	73	1
Ancona	AAA100D	JF716460	T2h	T2	T	126 153 240 289 294 296 519	73 150 	1
Ancona	AAB010D	JF716461	H	H	H	rCRS	rCRS	0
Ancona	AAB020D	JF716462	U5a	U5	U	243 256 270 526	73	1
Ancona	AAB030D	JF716463	R0a	R0a	R0	126 184 362	60+T 64 72G 	1
Ancona	AAB040D	JF716464	H	H	H	355 519	rCRS	1	Retinopathy, Somatic Neuropathy, Cardiac Ischemia
Ancona	AAB050D	JF716465	T2b	T2	T	126 241 294 296 304 519	73	1
Ancona	AAB060D	JF716466	H	H	H	093	rCRS	0
Ancona	AAB080D	JF716467	X2	X2	X	183C 189 223 278 325 519	73 152 195 	1	Retinopathy, Somatic Neuropathy
Ancona	AAB090D	JF716468	H5	H5	H	124 304 311	152	0
Ancona	AAB100D	JF716469	T2b	T2	T	126 294 296 304 519	73	1	Somatic Neuropathy
Ancona	AAC010D	JF716470	R0a	R0a	R0	126 184 362	60+T 64 72G 	1	Cardiac Ischemia
Ancona	AAC020D	JF716471	H	H	H	355 519	rCRS	1	Retinopathy, Cardiac Ischemia
Ancona	AAC050D	JF716472	H	H	H	093 274	56+C 93 183 	0
Ancona	AAC060D§	JF716473	U5a1	U5	U	256 270 293 399	73	0
Macerata	AAC070D	JF716474	N1b1	N1	N1-2	145 176G 223 258 291 294 390 519	73 152 	1	Cardiac Ischemia
Ancona	AAC080D	JF716475	V	V	HV	153 298	72 93 	1	Cardiac Ischemia
Ancona	AAC090D	JF716476	H1	H1	H	086 188G 301h 519	195	1	Retinopathy
Ancona	AAC100D§	JF716477	U5a1	U5	U	256 270 293 399	73	1	Retinopathy, Somatic Neuropathy, Cardiac Ischemia, Nephropathy, Renal Failure
Ancona	AAD010D	JF716478	H	H	H	519	rCRS	1
Ancona	AAD020D	JF716479	H	H	H	rCRS	rCRS	0
Ancona	AAD030D	JF716480	H1	H1	H	188G 519	rCRS	1	Retinopathy, Somatic Neuropathy, Nephropathy, Renal Failure
Ancona	AAD040D§	JF716481	U5a1	U5	U	256 270 399	44+C 73 	0
Ancona	AAD050D§	JF716482	W	W	N1-2	223 234 292 519	73 189 194 195 204 207 	0
Macerata	AAD070D	JF716483	K1	K1	K	224 278 311 519	73 195 	1
Ancona	AAD080D§	JF716484	U5a1	U5	U	256 270 293 399	73	0
Ancona	AAD090D§	JF716485	U8b	U8	U	183C 189 290 311 324 519	73 195 	1
Ancona	AAD100D	JF716486	H1	H1	H	188A 519	rCRS	1
Ancona	AAE020D	JF716487	H	H	H	145 519	64	1	Retinopathy, Somatic Neuropathy, Nephropathy
Ancona	AAE030D	JF716488	J1c	J1	J	069 126 319 343	73 185h 	0
Ancona	AAE040D	JF716489	H1	H1	H	172 183C 189 213 519	150	0
Ancona	AAE060D	JF716490	X2	X2	X	150 183C 188 189 223 278 519	73 153 195	1
Ascoli Piceno	AAE070D	JF716491	U2e	U2	U	051 129C 183C 189 362 519	42+G 73 152 217 	1	Retinopathy
Ancona	AAE080D	JF716492	H	H	H	rCRS	rCRS	0
Ancona	AAE090D	JF716493	T2b	T2	T	126 241 294 296 304 519	73	0
Ancona	AAE100D§	JF716494	U6a	U6	U	134 172 219 278 519	73 150 189 195 	1	Retinopathy
Ancona	AAF010D§	JF716495	U4	U4	U	356 519	73 195 	1	Somatic Neuropathy
Ancona	AAF020D§	JF716496	U5a1	U5	U	189 192 256 270 291 399	73	1	Retinopathy
Ancona	AAF030D§	JF716497	U8b	U8	U	183C 189 234 519	73 195 	1	Retinopathy, Somatic Neuropathy, Nephropathy, Renal Failure
Ancona	AAF040D§	JF716498	U7	U7	U	309 318T 362 519	60 61 73 152 	1	Retinopathy
Macerata	AAF050D	JF716499	H1	H1	H	093 172 189 519	rCRS	1
Ancona	AAF060D	JF716500	K1	K1	K	224 311 519	73	1	Somatic Neuropathy
Ancona	AAF070D	JF716501	J1c	J1	J	069 126 218 294 	73 152 	1	Retinopathy
Ancona	AAF080D	JF716502	H5	H5	H	304	204	1	Retinopathy, Cardiac Ischemia
Ancona	AAF090D	JF716503	H5	H5	H	304	rCRS	1	Nephropathy, Renal Failure
Ancona	AAF100D	JF716504	H5	H5	H	304	204	1	Retinopathy
Ancona	AAG010D	JF716505	I	I	N1-2	129 148 192 223 294 391 519 527	73 199 204 	1	Retinopathy, Somatic Neuropathy
Ancona	AAG030D	JF716506	J1c	J1	J	069 126 261	73 185 	0
Ancona	AAG040D	JF716507	H8	H8	H	068 288 362	146 195 	0
Macerata	AAG050D	JF716508	H1	H1	H	189 519	rCRS	1	Retinopathy, Somatic Neuropathy
Ancona	AAG060D	JF716509	H	H	H	274 287 519	rCRS	0
Ancona	AAG070D	JF716510	T2b	T2	T	126 294 296 304 519	73 152 	0
Ancona	AAG080D	JF716511	K1	K1	K	224 246T 311 519	73 152 	0
unspecified	AAG090D	JF716512	M1b2	M1	M	093 129 182C 183C 189 223 249 311 399 519	73 195 	1
Ancona	AAG100D	JF716513	T2b	T2	T	126 294 296 304 519	73	1	Retinopathy, Somatic Neuropathy, Cardiac Ischemia
Ancona	AAH010D	JF716514	R0a	R0a	R0	126 266 362 	58 60+T 64 	1	Cardiac Ischemia
Ancona	AAH020D	JF716515	H3	H3	H	104 176 311 519	rCRS	1	Somatic Neuropathy
Ancona	AAH030D§	JF716516	U5b3	U5	U	093 192 235 270 291 304	73 150 	1	Retinopathy, Nephropathy
Ancona	AAH040D	JF716517	K1	K1	K	037 093 224 311 519	73 195 207 	1
Ancona	AAH050D	JF716518	HV	HV	HV	274 311 	rCRS	1
Ancona	AAH070D	JF716519	H5	H5	H	304 311 319	rCRS	1
Ancona	AAH080D	JF716520	H	H	H	172 519	rCRS	1	Retinopathy
Ascoli Piceno	AAH090D	JF716521	X2	X2	X	179 189 223 278 519	73 153 195 	1
Ancona	AAH100D	JF716522	H3h	H3	H	311 519	rCRS	1	Retinopathy, Somatic Neuropathy, Nephropathy
Ancona	AAI010D	JF716523	K1	K1	K	224 300 311 519	73	1	Retinopathy, Somatic Neuropathy, Nephropathy
Ancona	AAI020D§	JF716524	U5a	U5	U	192 256 270 526	73 188 	0
Ancona	AAI050D§	JF716525	W	W	N1-2	223 292 294 519	73 189 195 204 207 	0
Ancona	AAI060D	JF716526	X2	X2	X	183C 189 223 278 519	73 153 195 	0
Ancona	AAI080D	JF716527	T2b	T2	T	126 241 294 296 304 519	73	1	Retinopathy, Somatic Neuropathy, Cardiac Ischemia, Peripheral Artery Occlusive Disease
Ancona	AAI090D	JF716528	H	H	H	rCRS	152	1	Somatic Neuropathy
Ancona	AAI100D	JF716529	H5	H5	H	304	131	1
Ancona	AAJ010D	JF716530	H5	H5	H	093 294 304 320	152	0
Ancona	AAJ020D§	JF716531	U7	U7	U	309 318T 362 519	60 61 73 152 	0
Ancona	AAJ050D§	JF716532	W	W	N1-2	223 292 294 519	73 189 195 204 207 	1
Ancona	AAJ060D	JF716533	V	V	HV	298 311 	72 195 200 	0
Ancona	AAJ070D	JF716534	HV0*	HV0	HV	265 298	151 152 	1
Ancona	AAJ090D§	JF716535	V	V	HV	298 390 519	72	1
Ancona	AAJ100D	JF716536	H5	H5	H	260 294 304	rCRS	0
Ancona	AAK010D	JF716537	H1	H1	H	209 519	rCRS	1	Retinopathy
Ancona	AAK020D	JF716538	H3	H3	H	093 368 519	rCRS	0
Ancona	AAK030D	JF716539	I	I	N1-2	075 129 169 223 260 391 519	73 199 204	0
Ancona	AAK040D	JF716540	H	H	H	086 311 	152	0
Ancona	AAK050D	JF716541	J1c	J1	J	069 126 218 294	73 152 	1	Peripheral Artery Occlusive Disease
Ancona	AAK060D	JF716542	K1	K1	K	037 093 224 311 519	73 195 207 	1	Somatic Neuropathy, Cardiac Ischemia
Ancona	AAK070D	JF716543	T2b	T2	T	095 126 294 304 519	73	1
Ancona	AAK080D	JF716544	HV	HV	HV	311	rCRS	1
Ancona	AAK090D	JF716545	H	H	H	261 291 519	200	0
Ancona	AAK100D	JF716546	T2	T2	T	126 294 296 519	73 200 	1	Retinopathy, Somatic Neuropathy, Cardiac Ischemia
Ancona	AAL020D	JF716547	K1	K1	K	224 311 362 519	73 195 	0
Ancona	AAL030D§	JF716548	U5a	U5	U	192 256 270 526	73	1
Ancona	AAL040D	JF716549	T1a	T1	T	126 163 186 189 294 519	73	1	Retinopathy, Somatic Neuropathy, Nephropathy
Ancona	AAL050D	JF716550	U8b	U8	U	183C 189 234 324 519	73 195 	1	Cardiac Ischemia
Ancona	AAL060D	JF716551	H	H	H	311 519 527	rCRS	0
Ancona	AAL070D	JF716552	M1b2	M1	M	093 129 182C 183C 189 223 249 311 399 519	73 195 	1
Ancona	AAL080D	JF716553	T2b	T2	T	126 294 296 304 519 527	73	0
Ancona	AAL090D	JF716554	H1	H1	H	519	73	1	Nephropathy
Ancona	AAL100D	JF716555	T1a	T1	T	126 163 186 189 222 292 294 519	73 150 214 	1
Ancona	AAM010D	JF716556	H	H	H	519	rCRS	0
Ancona	AAM020D	JF716557	T1a	T1	T	126 163 186 189 222 292 294 519	73 150 214 	1
Ancona	AAM030D§	JF716558	V	V	HV	153 298 519	72 93 	1
Ancona	AAM040D	JF716559	J1c	J1	J	069 126 218 294 	73 152 	1	Retinopathy, Somatic Neuropathy, Nephropathy, Renal Failure
Ancona	AAM050D	JF716560	H1	H1	H	519	rCRS	1	Cardiac Ischemia
Ancona	AAM060D	JF716561	H1	H1	H	519	152	0
Ancona	AAM070D§	JF716562	U6a	U6	U	134 172 219 278 519	73 150 189 195 	1	Retinopathy
Ancona	AAM080D	JF716563	K1	K1	K	075 224 311 519	73 146 152 	0
Ancona	AAM090D	JF716564	K1	K1	K	093 189 224 311 519	73 143 195	1	Somatic Neuropathy
Ancona	AAM100D	JF716565	H1	H1	H	188A 519	rCRS	1
Ancona	AAN020D§	JF716566	U5a	U5	U	114A 173 192 256 270 294 526	73	1
Ancona	AAN030D	JF716567	D4	D4	M	092 188 223 362 	73 197 	1
Ancona	AAN040D	JF716568	HV1	HV	HV	067 129 274 368	rCRS	0
Ancona	AAN050D	JF716569	T1a	T1	T	126 163 186 189 294 519	73 152 195 	0
Macerata	AAN070D	JF716570	K1	K1	K	182C 183C 189 222 224 311 519	73 146 	1
Ancona	AAN080D	JF716571	H1	H1	H	209 519	rCRS	0
Ancona	AAN090D	JF716572	T1a	T1	T	126 163 186 189 294 519	73	0
Ancona	AAN100D	JF716573	R0a	R0a	R0	126 230 362 497	60+T 64 	1	Retinopathy, Cardiac Ischemia, Nephropathy
Ancona	AAO010D	JF716574	K1	K1	K	224 311 519	73	1	Cardiac Ischemia
Ascoli Piceno	AAO020D	JF716575	N1b1	N1	N1-2	145 176G 223 258 291 294 390 519	73 152 	1	Retinopathy, Cardiac Ischemia
Ascoli Piceno	AAO030D	JF716576	T2b	T2	T	126 274 291 294 296 304 519	73	0
Ancona	AAO050D	JF716577	H1	H1	H	188A 519	rCRS	0
Ancona	AAO060D	JF716578	H	H	H	114 319 519	rCRS	1
Ancona	AAO070D	JF716579	K1	K1	K	093 183C 189 224 311 519	73 143 195 	1	Nephropathy
Ancona	AAO080D	JF716580	H1	H1	H	188A 519	rCRS	0
Ancona	AAO090D	JF716581	J1c	J1	J	069 126 183C 189	73 185 	1
Ancona	AAP010D	JF716582	HV1	HV	HV	067 153 256 278 292	93	0
Ancona	AAP030D	JF716583	T2b	T2	T	126 294 296 304 519 527	73	1	Retinopathy
Ancona	AAP050D	JF716584	H1	H1	H	188A 519	rCRS	0
Ancona	AAP060D	JF716585	HV1	HV	HV	067 183C 189 311	152	0
Ancona	AAP070D	JF716586	H	H	H	519	rCRS	1	Retinopathy, Somatic Neuropathy
Ancona	AAP080D	JF716587	T2a	T2	T	092A 126 214 294 296 324 519	73	1
Ancona	AAP090D	JF716588	H	H	H	183C 189 284 286 519	rCRS	1	Nephropathy
Ancona	AAP100D	JF716589	H	H	H	183C 189 284 286 519	rCRS	1	Retinopathy, Somatic Neuropathy
Ascoli Piceno	AAQ010D	JF716590	N1b1	N1	N1-2	145 176G 223 258 291 294 390 519	73 152 	0
Ascoli Piceno	AAQ020D§	JF716591	V	V	HV	189 298 	72	1	Retinopathy, Somatic Neuropathy, Cardiac Ischemia, Peripheral Artery Occlusive Disease
Ancona	AAQ030D§	JF716592	U4	U4	U	356 362 519	73 195 	0
Ancona	AAQ040D	JF716593	H6	H6	H	362 482	rCRS	1
Ancona	AAQ050D	JF716594	T2b	T2	T	126 294 296 304 519 527	73	1
Ancona	AAQ060D	JF716595	X2	X2	X	189 223 278 519	73 143 195 198 	1	Retinopathy
Ancona	AAQ070D	JF716596	H1	H1	H	254 311 368 519	150 195 	1	Cardiac Ischemia
Ancona	AAQ080D	JF716597	K1	K1	K	224 246T 311 519	73 152 	0
Ancona	AAQ090D§	JF716598	U4	U4	U	356 362 519	73 195 	1
Ancona	AAQ100D	JF716599	H	H	H	235 291 357 519	rCRS	1	Retinopathy
Ancona	AAR020D	JF716600	T2c	T2	T	126 186 292 294 519	73 146 	1
Ancona	AAR030D§	JF716601	V	V	HV	261 290 298	72	0
Ancona	AAR050D	JF716602	H1c	H1	H	193 519	rCRS	0
Ancona	AAR060D	JF716603	HV0*	HV0	HV	298 311 519	rCRS	1	Retinopathy
Ancona	AAR080D	JF716604	HV	HV	HV	311	rCRS	1
Ancona	AAR090D	JF716605	H	H	H	093 291 311	195	1	Retinopathy, Somatic Neuropathy
Ancona	AAR100D	JF716606	M1b2	M1	M	093 129 182C 183C 189 193+C 223 249 311 399 519	73 195 	0
Ancona	AAS010D	JF716607	T2c	T2	T	126 292 294 519	73 150 	1	Cardiac Ischemia, Nephropathy
Ancona	AAS030D	JF716608	H1b	H1	H	189 356 362 519	rCRS	1	Retinopathy
Ancona	AAS040D	JF716609	K1	K1	K	224 300 311 519	73	0
Ancona	AAS050D	JF716610	T2b	T2	T	126 292 294 296 304 368 519	55+T 57 59 73 151 152 	1
Ancona	AAS060D	JF716611	T2b	T2	T	126 292 294 296 304 368 519	55+T 57 59 73 151 152 	1	Retinopathy
Ancona	AAS070D	JF716612	J1c	J1	J	069 126	73 185 	1	Retinopathy, Somatic Neuropathy
Ancona	AAS090D	JF716613	H	H	H	311	228	1	Retinopathy, Cardiac Ischemia, Nephropathy, Renal Failure
Ancona	AAS100D	JF716614	H3h	H3	H	311 519	rCRS	1	Somatic Neuropathy
Ancona	AAT010D	JF716615	H	H	H	261 291 519	200	1	Retinopathy
Ancona	AAT020D	JF716616	H	H	H	188 311 519	rCRS	1
Macerata	AAT030D	JF716617	H9	H9	H	168 355 519	152	1	Peripheral Artery Occlusive Disease
Ancona	AAT040D	JF716618	D4	D4	M	092 188 223 362 	73 197 	0
Ancona	AAT050D§	JF716619	U5a	U5	U	114A 173 192 256 270 294 526	73	1
Ancona	AAT060D§	JF716620	U5a	U5	U	114A 173 192 256 270 294 526	73	0
Ancona	AAT070D	JF716621	H	H	H	218 519	rCRS	1	Retinopathy, Somatic Neuropathy
Ancona	AAT080D	JF716622	T2b	T2	T	126 294 296 304 519	73 152 204 	0
Ancona	AAT090D	JF716623	K1	K1	K	224 311 519	73	1
Ancona	AAU010D	JF716624	N1b1	N1	N1-2	145 176G 223 244 390 519	73 146 	1
Ancona	AAU020D§	JF716625	U5a	U5	U	243 256 270 526	73	0
Ancona	AAU030D	JF716626	HV	HV	HV	311	rCRS	1
Ancona	AAU040D	JF716627	H1	H1	H	519	rCRS	0
Ancona	AAU050D	JF716628	L3d	L3	L	124 223 519	73 152 	1
Ancona	AAU060D	JF716629	T2h	T2	T	126 153 240C 289 294 296 519	73 150 	1
Ancona	AAU080D	JF716630	H1	H1	H	519	rCRS	1
Ancona	AAU090D	JF716631	H1	H1	H	519	rCRS	0
Ancona	AAU100D	JF716632	HV	HV	HV	rCRS	rCRS	0
Ancona	AAV010D	JF716633	J2b	J2	J	069 126 193 278 311	73 150 152 	0
Ancona	AAV020D	JF716634	H5	H5	H	304 311 319	rCRS	1	Retinopathy
Ancona	AAV030D§	JF716635	U7	U7	U	309 318T 362 519	60 61 73 152 	0
Ancona	AAV040D	JF716636	T1a	T1	T	126 163 186 189 294 519	73	1	Peripheral Artery Occlusive Disease
unspecified	AAV050D§	JF716637	U5b	U5	U	192 270	73 150 199 	0
Ancona	AAV060D	JF716638	K1	K1	K	224 278 311 519	73 195 	1	Retinopathy
Ancona	AAV070D	JF716639	K1	K1	K	224 278 311 519	73 195 	1	Retinopathy
Ancona	AAV090D§	JF716640	U5b	U5	U	093 189 270 	73 150 	1
Ancona	AAV100D	JF716641	J1c	J1	J	069 126 189	73 185 	1	Cardiac Ischemia
Ancona	AAW010D	JF716642	T2h	T2	T	126 153 240C 274 289 294 296 519	73 150	0
Ancona	AAW020D	JF716643	T2b	T2	T	126 145 294 304 519	73 152 	1
Ancona	AAW030D	JF716644	H1	H1	H	311 519	rCRS	0
Ancona	AAW040D	JF716645	J1b	J1	J	069 126 145 222 261 519	64 73 152 	1	Nephropathy
Ancona	AAW050D	JF716646	K1	K1	K	037 093 224 311 519	73 195 207 	1	Retinopathy
Ancona	AAW060D	JF716647	H3h	H3	H	311 519	rCRS	1
Macerata	AAW070D	JF716648	H9	H9	H	168 355 519	152	1	Retinopathy
unspecified	AAW080D	JF716649	H	H	H	519	93	1
Ancona	AAW090D	JF716650	I	I	N1-2	129 223 311 391 519	73 152 199 204 	1	Nephropathy
Ancona	AAW100D	JF716651	H	H	H	519	rCRS	0
Ancona	AAY010D§	JF716652	U5a1	U5	U	256 270 399	42+C 73 	0
Ancona	AAY020D	JF716653	H	H	H	354	rCRS	1	Retinopathy
Ancona	AAY030D§	JF716654	U5b3	U5	U	270 304 311	73 150 	0
Ancona	AAY040D	JF716655	T2c	T2	T	086 292 294 296 519	73 89 146 	1	Cardiac Ischemia
Ancona	AAY050D§	JF716656	U5a1	U5	U	256 270 399	73 146 207 	0
Ancona	AAY060D	JF716657	H1	H1	H	209 519	rCRS	1	Retinopathy, Somatic Neuropathy, Cardiac Ischemia, Nephropathy, Peripheral Artery Occlusive Disease, Renal Failure
Ancona	AAY070D	JF716658	X2	X2	X	189 223 278 519	73 153 195	0
Ancona	AAY080D	JF716659	H	H	H	354	rCRS	1	Peripheral Artery Occlusive Disease
Ancona	AAY100D	JF716660	T2c	T2	T	086 126 292 294 296 519	73 89 146 	0
Ancona	AAZ020D§	JF716661	U5b	U5	U	192 270 	73 150 199 	0
Ancona	AAZ030D§	JF716662	U5a1	U5	U	256 270 294 399	73 152 	1	Cardiac Ischemia
Ancona	AAZ040D	JF716663	D4	D4	M	092 188 223 362 	73 197 	1
Ancona	AAZ050D§	JF716664	U5a1	U5	U	256 270 294 399	73 152 	1	Retinopathy, Somatic Neuropathy, Cardiac Ischemia, Peripheral Artery Occlusive Disease
Ancona	AAZ070D§	JF716665	U7	U7	U	309 318T 362 519	60 61 73 152 	1
Ancona	AAZ080D	JF716666	J1c	J1	J	069 126 189 319 343 	73 185 	0
unspecified	AAZ090D	JF716667	I	I	N1-2	129 148 192 223 294 391 519 527	73 199 204	1	Peripheral Artery Occlusive Disease
Ancona	AAZ100D	JF716668	N1b1	N1	N1-2	145 176G 188 223 390 519	73 151 152 200 	1	Retinopathy, Cardiac Ischemia, Nephropathy, Peripheral Artery Occlusive Disease, Renal Failure
Ancona	ABA010D	JF716669	J1c	J1	J	069 126	73	1
Ancona	ABA020D	JF716670	J1c	J1	J	069 126	73	1	Retinopathy, Somatic Neuropathy
Ancona	ABA040D	JF716671	H1	H1	H	129 519	rCRS	1
Ancona	ABA050D§	JF716672	U8b	U8	U	079 129 182C 183C 189 311 399	73 195 	1	Retinopathy, Somatic Neuropathy, Nephropathy, Peripheral Artery Occlusive Disease
Ancona	ABA060D	JF716673	H1	H1	H	183C 189 519	rCRS	0
Ancona	ABA080D	JF716674	U1a	U1	U	183C 189 242 249 294 362	73	0
Ancona	ABA090D§	JF716675	U5a1	U5	U	256 270 399	73 146 207 	1	Retinopathy, Somatic Neuropathy
Ancona	ABA100D	JF716676	H1	H1	H	320 519	146	0
Ancona	ABB020D	JF716677	H	H	H	519	rCRS	0
Ancona	ABB030D	JF716678	H1	H1	H	254 311 368 519	66T 150 195 	1
Ancona	ABB050D	JF716679	H3h	H3	H	311 519	rCRS	0
Ancona	ABB060D	JF716680	H1c	H1	H	193 519	rCRS	0
Ancona	ABB070D	JF716681	H	H	H	rCRS	64 93 	1	Retinopathy, Somatic Neuropathy
Ancona	ABB080D	JF716682	T2c	T2	T	086 126 292 294 296 519	73 89 146 	0
Ancona	ABB090D§	JF716683	V	V	HV	162 298 519 	72	1
Ancona	ABB100D	JF716684	K1	K1	K	187 224 311 519	73 152 	0
Ancona	ABC010D§	JF716685	U5b	U5	U	183C 189 270 357 519	73 150 	1	Retinopathy
Ancona	ABC020D	JF716686	H	H	H	080 519	rCRS	1
Ancona	ABC030D	JF716687	H1c	H1	H	193 519	rCRS	0
Ancona	ABC040D§	JF716688	U5b	U5	U	183C 189 270 357 519	73 150 	1
Ancona	ABC050D	JF716689	T2b	T2	T	126 256 294 296 304 519	73 150 151 	1
Ancona	ABC060D	JF716690	H1	H1	H	129 519	rCRS	1
unspecified	ABC070D	JF716691	H3	H3	H	519	152	0
Ascoli Piceno	ABC080D	JF716692	T2a	T2	T	126 294 324 519	73	0
Macerata	ABC090D	JF716693	K1	K1	K	037 093 224 311 519	73 195 207 	1	Peripheral Artery Occlusive Disease
Ancona	ABC100D	JF716694	J1c	J1	J	069 126 319 343 	73 185 	1	Retinopathy
Ancona	ABD010D	JF716695	HV	HV	HV	189 519	153 204 207 	1	Cardiac Ischemia
Ancona	ABD030D	JF716696	D4	D4	M	092 188 223 362 	73 197 	1
Ancona	ABD040D	JF716697	K1	K1	K	037 093 224 311 519	73 195 207	1	Retinopathy
Ancona	ABD050D	JF716698	U3b	U3	U	343 519	73 150 	1
Ancona	ABD060D	JF716699	H1	H1	H	519	rCRS	1	Somatic Neuropathy
Ancona	ABD070D	JF716700	H	H	H	093 129 189 234 311 316 519	rCRS	0
unspecified	ABD080D	JF716701	U1a	U1	U	182C 183C 189 249	73	1	Retinopathy, Nephropathy
Ancona	ABD090D	JF716702	HV0*	HV0	HV	153 298 	72 93 	1	Somatic Neuropathy, Nephropathy
Ancona	ABD100D	JF716703	N1b1	N1	N1-2	145 176G 223 390 519	73 152 	1
Ancona	ABE050D	JF716704	HV	HV	HV	217	72 73 152 195 	1
Ancona	ABE060D	JF716705	H	H	H	311 355 519	195	0
unspecified	ABE080D	JF716706	J1c	J1	J	069 126 147A 519	73 185 	0
Ancona	ABE090D	JF716707	H	H	H	183C 189 284 286 519	rCRS	1
Ancona	ABE100D	JF716708	H	H	H	311 355 519	195	1
Ancona	ABF010D	JF716709	H6	H6	H	362 482	rCRS	1	Retinopathy
Ancona	ABF020D	JF716710	J1c	J1	J	069 126 147A 519	73 185 	0
Ancona	ABF030D	JF716711	D4	D4	M	092 188 223 362 	73	0
Ancona	ABF040D	JF716712	M1a2a	M1	M	129 183C 189 193+C 223 249 311 519	73 152 195 214 	0
Ancona	ABF050D	JF716713	J1c	J1	J	069 126 147A 519	73 185 	1	Peripheral Artery Occlusive Disease
Ancona	ABF060D	JF716714	H6	H6	H	111 362 482	rCRS	1	Cardiac Ischemia
Ancona	ABF070D	JF716715	U5a1	U5	U	256 270 294 399	73 152 	1
Ancona	ABF090D	JF716716	X2	X2	X	093 183+A 189 223 265 278 519	73 153 195 	1
Ancona	ABF100D	JF716717	H3	H3	H	189 519	rCRS	0
Ancona	ABG010D§	JF716718	W	W	N1-2	223 292 519	73 143 153 189 194 195 196 204 207 	0
Ancona	ABG020D§	JF716719	U4	U4	U	265 356 362 519	73 195 	1	Cardiac Ischemia
Ancona	ABG030D	JF716720	J1c	J1	J	069 126 183C 189 224	73 185 	1
Ancona	ABG040D	JF716721	H6	H6	H	362 482	rCRS	1	Retinopathy
Ancona	ABG060D	JF716722	J1b	J1	J	069 126 145 150 172 222 260 261	73	0
Ancona	ABG080D	JF716723	D4	D4	M	092 188 223 362 	73	1
Ancona	ABG090D	JF716724	H3	H3	H	093 368 519	rCRS	1	Retinopathy, Somatic Neuropathy
Ancona	ABG100D	JF716725	H1	H1	H	519	60A 152 	0
Ancona	ABH010D	JF716726	J1c	J1	J	069 126 234	73	1
Ancona	ABH020D	JF716727	H	H	H	319 325 519	rCRS	1	Cardiac Ischemia, Nephropathy, Renal Failure
Ancona	ABH030D	JF716728	HV	HV	HV	234 311	rCRS	0
Ancona	ABH040D	JF716729	H1a1	H1	H	162 209 519	73	0
Ancona	ABH060D	JF716730	HV	HV	HV	234 311	rCRS	0
Ancona	ABH080D§	JF716731	U5a1	U5	U	192 256 270 399 519	73	0
Ancona	ABH090D§	JF716732	U7	U7	U	309 318T 362 519	60 61 73 152 	1
Ancona	ABH100D	JF716733	T1a	T1	T	126 163 186 189 294 519	73 152 195 	0
Ancona	ABI010D	JF716734	H6	H6	H	362 482 519	rCRS	1	Retinopathy, Somatic Neuropathy
Ancona	ABI030D§	JF716735	V	V	HV	298 311	72 195 200 	1
Ancona	ABI040D	JF716736	H1	H1	H	519 519	rCRS	0
Ancona	ABI050D	JF716737	K1	K1	K	224 311 519	73	1	Retinopathy
Ancona	ABI060D§	JF716738	U5a1	U5	U	192 256 270 399 519	73	0
Ancona	ABI070D	JF716739	T1a	T1	T	126 163 176 186 189 294 311 519	73 146 150 152 	1
unspecified	ABI100D	JF716740	T2c	T2	T	126 186 292 294 296 519	73 146 	1
Ancona	ABJ040D	JF716741	H6	H6	H	362 482	rCRS	0
Ancona	ABJ050D	JF716742	J1c	J1	J	069 126 183C 189 	73 185 189 	0
Ancona	ABJ060D	JF716743	L3d	L3	L	124 223 519	73 152 	0
Ancona	ABJ070D	JF716744	H	H	H	080 519	rCRS	1	Retinopathy, Somatic Neuropathy, Cardiac Ischemia
Ancona	ABJ080D	JF716745	K1	K1	K	129 145 224 311 519	73	1
Ancona	ABJ090D	JF716746	J1b	J1	J	069 126 145 261 311 	73	1	Retinopathy
Ancona	ABJ100D	JF716747	T2b	T2	T	126 294 296 304 519	73	1
Ancona	ABK010D	JF716748	H5	H5	H	304 311	207h 	1	Somatic Neuropathy
unspecified	ABK030D§	JF716749	U5a1	U5	U	256 270 294 399	73 152 	1
Ancona	ABK040D	JF716750	H	H	H	261 286 519	204	1
Ascoli Piceno	ABK050D§	JF716751	H3	H3	H	519	152	0
Ancona	ABK060D	JF716752	HV	HV	HV	172 311 	143	1	Somatic Neuropathy
Macerata	ABK070D	JF716753	H3h	H3	H	311 519	rCRS	1	Somatic Neuropathy
Ancona	ABK080D	JF716754	M1a1	M1	M	129 189 223 249 311 359 519	73 195 	1
Ancona	ABK090D	JF716755	N1b1	N1	N1-2	145 176G 188 223 390 519	73 151 152 200 	1	Retinopathy
unspecified	ABL010D§	JF716756	U4	U4	U	356 519	73 152 195 	1
Ancona	ABL020D	JF716757	U8b	U8	U	183C 189 234 324 519	73 195 	0
Macerata	ABL030D§	JF716758	U4	U4	U	356 519	73 195 	1
Ancona	ABL040D	JF716759	J1c	J1	J	069 126 	73 185 	0
Ancona	ABL050D	JF716760	H	H	H	rCRS	rCRS	0
Ancona	ABL070D§	JF716761	U4	U4	U	134 356 519	73 152 195 	1	Cardiac Ischemia
Ancona	ABL090D	JF716762	X2	X2	X	150 183C 188 189 223 278 519	73 153 195 	1
unspecified	ABL100D	JF716763	H	H	H	rCRS	rCRS	1	Retinopathy, Somatic Neuropathy, Cardiac Ischemia
Ancona	ABM010D	JF716764	K1	K1	K	224 311 519	73	0
Ancona	ABM020D	JF716765	H3h	H3	H	311 519	152	0
Ancona	ABM030D	JF716766	H	H	H	189 519	rCRS	1
Ancona	ABM040D	JF716767	H1c	H1	H	166 519	rCRS	1
Ancona	ABM050D§	JF716768	U5a1	U5	U	256 270 294 399	73 152 	1
Ancona	ABM060D	JF716769	H	H	H	rCRS	rCRS	1
Ancona	ABM080D	JF716770	J2a	J2	J	069 126 145 172 231 261 519	73 150 152 195 	0
Ancona	ABM090D	JF716771	H1	H1	H	519	rCRS	1	Retinopathy, Peripheral Artery Occlusive Disease
Ancona	ABN010D	JF716772	U8b	U8	U	183C 189 290 311 324 519	73 195 	1	Retinopathy
Ancona	ABN020D	JF716773	D4	D4	M	092 188 223 362 	73	0
Ancona	ABN040D	JF716774	HV1	HV	HV	067 265	rCRS	1
Ancona	ABN050D	JF716775	T2b	T2	T	093 126 294 304 519	73 146 199 	0
Ancona	ABN060D	JF716776	H	H	H	274 287 519	rCRS	1
Ancona	ABN070D	JF716777	H6	H6	H	154 362 482	rCRS	0
Ancona	ABN080D	JF716778	H	H	H	519	rCRS	1	Retinopathy, Cardiac Ischemia
Ancona	ABN100D	JF716779	H	H	H	rCRS	146	1
Ancona	ABO010D	JF716780	HV	HV	HV	172 311	143	1
Ancona	ABO020D	JF716781	H	H	H	134 362	152 199 	1
Ancona	ABO040D	JF716782	H3	H3	H	129	73	0
Ancona	ABO050D	JF716783	H	H	H	rCRS	56+C 	0
Ancona	ABO100D	JF716784	K1	K1	K	224 311 519	73	0
Ascoli Piceno	ABP010D	JF716785	H5	H5	H	304 519	152	0
unspecified	ABP020D	JF716786	H6	H6	H	154 362 482	rCRS	0
Ancona	ABP030D	JF716787	T2b	T2	T	126 294 304 519	73	1
Ancona	ABP050D	JF716788	HV	HV	HV	311	rCRS	1	Cardiac Ischemia
Ancona	ABP060D	JF716789	HV0*	HV0	HV	189 298	72 195 	0
unspecified	ABP070D	JF716790	H3h	H3	H	311 519	rCRS	1	Retinopathy, Somatic Neuropathy, Cardiac Ischemia
Ancona	ABP080D	JF716791	T2	T2	T	093 126 266 294 296 519	73	1
Ancona	ABQ020D§	JF716792	V	V	HV	189 298	72	1	Somatic Neuropathy, Cardiac Ischemia, Nephropathy, Renal Failure
Ancona	ABQ030D	JF716793	I	I	N1-2	129 223 311 391 519	73 152 199 204 	1
unspecified	ABQ060D	JF716794	U8b	U8	U	129 183C 189 234 519	73 195 	0
Ancona	ABQ070D§	JF716795	U5a1	U5	U	256 270 294 399	73 152 	1	Retinopathy, Somatic Neuropathy, Nephropathy, Peripheral Artery Occlusive Disease
Ancona	ABQ080D§	JF716796	U5b3	U5	U	093 192 235 270 291 304	73 150 	1	Cardiac Ischemia
Ancona	ABQ100D	JF716797	T2c	T2	T	126 186 291 292 294 296 519	73 146 	1
Ancona	ABR010D	JF716798	T2c	T2	T	126 186 291 292 294 296 519	73 146 	1	Retinopathy
Ancona	ABR020D	JF716799	T2a	T2	T	092A 126 294 296 324 519	73	1	Somatic Neuropathy
Ancona	ABR030D	JF716800	H1	H1	H	519	rCRS	0
Ancona	ABR040D	JF716801	T2a	T2	T	092A 126 294 296 324 519	73	1
unspecified	ABR050D§	JF716802	U5b	U5	U	093 189 270	73 150 	1	Somatic Neuropathy, Cardiac Ischemia
Ancona	ABR060D	JF716803	H5	H5	H	093 304 399	146 152 	1	Somatic Neuropathy
Ancona	ABR070D	JF716804	H5	H5	H	086 214 304	146	0
Ancona	ABR080D	JF716805	T2b	T2	T	093 126 294 304 519	73 199 	0
Ancona	ABR090D	JF716806	J1c	J1	J	069 126	73 185 188 	1
Ancona	ABR100D	JF716807	J1c	J1	J	069 126	73 185 188 	1	Cardiac Ischemia
Ancona	ABS040D	JF716808	J2a	J2	J	069 126 169	73 150 195 	0
Ancona	ABS050D	JF716809	T2b	T2	T	093 126 294 304 519	73 199 	1
Ancona	ABS060D	JF716810	H1	H1	H	172 519	93	0
Ancona	ABT030D	JF716811	T2b	T2	T	126 294 296 304 519	73	0
Ancona	ABT040D	JF716812	H9	H9	H	168 355 519	152	1
unspecified	ABT050D	JF716813	T2b	T2	T	126 294 296 304 519	73	1	Retinopathy, Nephropathy
Ancona	ABT070D	JF716814	X2	X2	X	183C 189 223 278 325 519	73 152 195 	1	Peripheral Artery Occlusive Disease
Macerata	ABT080D§	JF716815	U4	U4	U	134 356 519	73 152 195 	1
Ancona	ABT090D	JF716816	H9	H9	H	168 355 519	152	1
Ancona	ABT100D§	JF716817	U5a1	U5	U	192 256 270 399	73 195 	1	Retinopathy
Macerata	ABU010D	JF716818	H	H	H	086 311	152	1	Retinopathy, Somatic Neuropathy, Peripheral Artery Occlusive Disease
Macerata	ABU040D	JF716819	X2	X2	X	182C 183C 189 223 255 278 519	73 153 195 	0
Ancona	ABU050D§	JF716820	V	V	HV	298 301	72 200 	1	Cardiac Ischemia, Nephropathy, Renal Failure
Macerata	ABU060D	JF716821	T2c	T2	T	086 126 292 294 296 519	73 89 146 	1	Nephropathy
Ancona	ABU070D§	JF716822	U3b	U3	U	193 249 343 526	73 150 	1	Somatic Neuropathy, Nephropathy
Ancona	ABU090D	JF716823	J2a	J2	J	069 126 438	73 150 152 	0
Ancona	ABU100D	JF716824	H1	H1	H	254 311 368 519	150 195 	1	Retinopathy, Somatic Neuropathy, Cardiac Ischemia
Ancona	ABV010D	JF716825	H5	H5	H	093 294 304 320 	152	1
Ancona	ABV020D	JF716826	H	H	H	rCRS	rCRS	1
Ancona	ABV030D	JF716827	T2c	T2	T	126 146 292 294 519	73 150 	0
Ancona	ABV040D	JF716828	H1	H1	H	290 519	152	1	Retinopathy
Ancona	ABV050D	JF716829	T2	T2	T	126 294 296 320 344 519	73	0
Ancona	ABV060D	JF716830	H5	H5	H	086 214 304	146	1	Cardiac Ischemia
Ancona	ABV070D†	JF716831	T2b	T2	T	126 294 296 304 519	73	1	Cardiac Ischemia, Nephropathy
Ancona	ABV080D§	JF716832	U5a1	U5	U	256 270 399	73	0
Ancona	ABV100D	JF716833	H	H	H	183C 189 320 519	204	1	Nephropathy, Renal Failure
Ancona	ABW020D	JF716834	L3e2b	L3	L	172 183C 189 223 288 320 519	73 150 195 	0
Ancona	ABW030D	JF716835	J1c	J1	J	069 126	73	0
Ancona	ABW050D	JF716836	H	H	H	092 188+C 245 519	rCRS	1	Retinopathy, Peripheral Artery Occlusive Disease
Ancona	ABW060D	JF716837	T2	T2	T	126 148 201 294 296 311 519	73 200 	0
Ancona	ABW070D	JF716838	H	H	H	rCRS	rCRS	1
Ancona	ABW090D	JF716839	H	H	H	519	rCRS	1
Ancona	ABW100D§	JF716840	U5b	U5	U	174 189 260 270 311	73 150 	1
Ancona	ABY010D	JF716841	J1c	J1	J	069 126 218 294 	73 152 	1	Somatic Neuropathy, Cardiac Ischemia, Nephropathy, Renal Failure
Ancona	ABY020D†	JF716842	H1	H1	H	129 519	rCRS	1	Retinopathy, Nephropathy, Peripheral Artery Occlusive Disease
Ancona	ABY030D†	JF716843	V	V	HV	298 390 519	72	1
Ancona	ABY040D	JF716844	T2c	T2	T	126 146 292 294 519	73 150 	0
Ancona	ABY050D§	JF716845	U3b	U3	U	343	73 150 	1	Retinopathy, Somatic Neuropathy, Cardiac Ischemia, Nephropathy, Renal Failure
Ancona	ABY060D	JF716846	H	H	H	183C 189 320 519	204	1
Ancona	ABY070D	JF716847	H1	H1	H	519	rCRS	1
Ancona	ABY080D†	JF716848	V	V	HV	153 298 519	72 93 	1	Somatic Neuropathy, Peripheral Artery Occlusive Disease
Ancona	ABY090D	JF716849	U3a	U3	U	343 390 519	73 150 185 	1
Ancona	ABZ010D†	JF716850	V	V	HV	298 311	72 195 200 	1	Retinopathy
Ancona	ABZ020D	JF716851	U3a	U3	U	343 390 519	73 150 185 	1
Macerata	ABZ030D	JF716852	T2b	T2	T	126 187 294 295 296 304 519	73	0
Ancona	ABZ040D	JF716853	M1b2	M1	M	093 129 182C 183C 189 223 249 311 399 519	73 195 	1	Cardiac Ischemia
Ancona	ABZ050D	JF716854	H	H	H	093 274	56+C 93 183 	1
Ancona	ABZ060D†	JF716855	V	V	HV	290 298 	72	1
Ancona	ABZ070D	JF716856	M1b2	M1	M	093 129 182C 183C 189 223 249 311 399 519	73 195 	1	Cardiac Ischemia, Peripheral Artery Occlusive Disease
Ancona	ABZ080D	JF716857	U4	U4	U	356 519	73 195 	1	Cardiac Ischemia
Ancona	ABZ090D	JF716858	H5	H5	H	260 294 304 	rCRS	0
Ancona	ABZ100D	JF716859	T2b	T2	T	126 294 304 519	73 146 	1
Ancona	ACA010D†	JF716860	V	V	HV	290 298 	72	1	Retinopathy, Somatic Neuropathy
Ancona	ACA020D	JF716861	H	H	H	rCRS	152	1	Retinopathy, Nephropathy
Ancona	ACA030D	JF716862	H1	H1	H	183C 189 519	rCRS	1
Ancona	ACA040D†	JF716863	V	V	HV	189 298	72	1	Retinopathy, Somatic Neuropathy, Nephropathy, Renal Failure
Ancona	ACA050D	JF716864	H6	H6	H	362 482 519	rCRS	1	Somatic Neuropathy, Nephropathy
Ancona	ACA070D	JF716865	U3a	U3	U	343 390 519	73 150 185 	0
Ancona	ACA080D	JF716866	K1	K1	K	224 300 311 519	73	1
Ancona	ACA090D	JF716867	K1	K1	K	224 311 519	73 146 152 194 	1	Somatic Neuropathy
Ascoli Piceno	ACB010D§	JF716868	U5b	U5	U	189 192 270 311 336 519	55A 73 150 	1
Ancona	ACB020D	JF716869	U1a	U1	U	182C 183C 189 249	73	1	Retinopathy
Ancona	ACB030D§	JF716870	W	W	N1-2	223 260h 292 318 519	73 189 194 195 204 207 	0
Ancona	ACB040D	JF716871	K1	K1	K	224 300 311 519	73	0
Ancona	ACB050D	JF716872	K1	K1	K	093 224 311 519	73 152 	1
Ancona	ACB060D	JF716873	I	I	N1-2	129 223 391 519	73 152 199 204 	1	Somatic Neuropathy, Cardiac Ischemia
Ancona	ACB070D†	JF716874	V	V	HV	298 301	72	1
Ancona	ACB080D	JF716875	H	H	H	129 519	rCRS	1	Nephropathy
Ancona	ACB090D	JF716876	H1	H1	H	rCRS	152	0
Ancona	ACC010D	JF716877	HV1	HV	HV	067 183C 189	152	0
Ancona	ACC020D	JF716878	T2	T2	T	126 189 294 296 519	73 146 	1
Ancona	ACC040D	JF716879	H	H	H	183C 189 519	146 153 204 	1
Macerata	ACC050D	JF716880	H	H	H	192 519	73 152 	1
Ancona	ACC090D	JF716881	H5	H5	H	172 304 311	rCRS	1
Ancona	ACD020D	JF716882	H	H	H	189 284 286 519	rCRS	0
Ancona	ACD030D	JF716883	T2b	T2	T	126 294 296 304 519	73 199 	1	Retinopathy
Ancona	ACD040D	JF716884	U3a	U3	U	343 390 519	73 150 185 	1
Ancona	ACD060D§	JF716885	U3b	U3	U	092 104 343 519	73 146 150 	0
Ancona	ACD080D	JF716886	X2	X2	X	183C 189 223 278 325 519	71+G 73 152 195 	1	Retinopathy, Peripheral Artery Occlusive Disease
Ancona	ACD090D†	JF716887	V	V	HV	298	72	1	Retinopathy, Cardiac Ischemia
Ancona	ACD100D	JF716888	H	H	H	519	rCRS	0
Ancona	ACE010D	JF716889	H1	H1	H	519	rCRS	1
Ancona	ACE020D§	JF716890	W	W	N1-2	176 223 292 519	73 143 153 189 194 195 196 204 207 	0
Ancona	ACE040D	JF716891	T2b	T2	T	126 292 294 296 304 368 519	55+T 57 59 73 151 152 	1	Somatic Neuropathy
Ancona	ACE050D§	JF716892	X2	X2	X	183C 189 223 278 325 519	73 152 195 	0
Ancona	ACE060D	JF716893	H1	H1	H	519	rCRS	0
Ancona	ACE070D§	JF716894	X2	X2	X	183C 189 223 278 325 519	73 152 195 	0
Ancona	ACE100D§	JF716895	U5b	U5	U	174 189 260 270 311 	73 150 	0
unspecified	ACF010D	JF716896	U4	U4	U	261 356 519	73 195 	0
Ancona	ACF020D§	JF716897	V	V	HV	153 298	72 93 	1	Somatic Neuropathy
Ancona	ACF030D	JF716898	H	H	H	129 311 519	152	1	Retinopathy
Macerata	ACF040D	JF716899	U4	U4	U	261 356 519	73 195 	1
Ancona	ACF050D§	JF716900	U5b3	U5	U	093 192 235 270 291 304	73 150 	0
Ancona	ACF060D§	JF716901	U5b	U5	U	183C 189 270 519	73 150 	1	Somatic Neuropathy
Ancona	ACF080D	JF716902	H1	H1	H	086 519	rCRS	0
Ancona	ACF090D	JF716903	T2b	T2	T	126 294 296 304 519	73 152 200 	1
Ancona	ACF100D	JF716904	U4	U4	U	134 356 519	73 152 195 	1
Ancona	ACG010D	JF716905	K1	K1	K	224 311 519	73	1	Somatic Neuropathy
Ancona	ACG040D	JF716906	I	I	N1-2	129 223 260 391 519	73 152 199 204 207 	1	Somatic Neuropathy
Ancona	ACG050D§	JF716907	W	W	N1-2	223 292 318 519	73 189 194 195 204 207 	1
Ancona	ACG060D	JF716908	HV	HV	HV	217	72 73 152 195 	0
Ancona	ACG070D	JF716909	J2a	J2	J	069 126 145 231 261	73 150 152 195 215 	1
Ancona	ACG100D	JF716910	T2b	T2	T	126 294 296 304 519	73 152 200 	1
Ancona	ACH020D§	JF716911	U5a1	U5	U	192 256 270 399 519	73	1
Macerata	ACH030D	JF716912	J1d	J1	J	069 126 186 193 519	73 152 	1
Ancona	ACH040D§	JF716913	U5b3	U5	U	093 192 270 304	73 150 	1
Ancona	ACH050D	JF716914	K1	K1	K	223 224 311 519	73	1	Cardiac Ischemia
Ancona	ACH070D	JF716915	H1	H1	H	519	rCRS	0
Ancona	ACH080D	JF716916	K2	K2	K	224 311 519	73 146 152 	1	Retinopathy
Ancona	ACH100D	JF716917	HV	HV	HV	311 362	rCRS	1
Ancona	ACI010D	JF716918	H	H	H	093 291 311 	195	1
Ancona	ACI020D	JF716919	U3a	U3	U	343 390 519 527	73 150 185 	1	Retinopathy, Nephropathy
Ancona	ACI030D	JF716920	J1b	J1	J	069 126 145 222 261 519	64 73 152 	1
Ancona	ACI040D§	JF716921	X2	X2	X	183C 189 223 278 325 519	73 146h 152 195 	0
Ancona	ACI050D	JF716922	H5	H5	H	304	rCRS	1
Macerata	ACI060D§	JF716923	U3b	U3	U	166d 343	73 150 185 	1	Somatic Neuropathy
Macerata	ACI070D	JF716924	H	H	H	184 519	rCRS	0
Ancona	ACI090D	JF716925	U8b	U8	U	183C 189 234 324 519	73 195 	0
Ancona	ACI100D	JF716926	I	I	N1-2	111G 129 223 391 519	73 199 204 	0
Ancona	ACJ010D	JF716927	H5	H5	H	304	rCRS	0
Ancona	ACJ030D	JF716928	H	H	H	142 325	146	1
Ancona	ACJ040D	JF716929	H6	H6	H	362 482 519	rCRS	1
Ancona	ACJ050D	JF716930	H	H	H	154 519	rCRS	1
Ancona	ACJ060D	JF716931	H	H	H	519	rCRS	0
Ancona	ACJ070D	JF716932	H	H	H	519	rCRS	0
Ancona	ACJ090D§	JF716933	V	V	HV	290 298 	72	0
unspecified	ACJ100D	JF716934	H	H	H	150 311 519	rCRS	0
unspecified	ACK010D	JF716935	T2b	T2	T	126 294 296 304 519 527	73	1	Retinopathy
Ancona	ACK020D	JF716936	H1	H1	H	519	rCRS	0
Ancona	ACK030D	JF716937	T2b	T2	T	126 294 296 304 519 527	73	1
Ancona	ACK040D§	JF716938	U5b	U5	U	129 189 192 270 274 311 	73 150	0
Macerata	ACK050D	JF716939	H1	H1	H	189 356 519	rCRS	1	Somatic Neuropathy, Nephropathy
Macerata	ACK060D§	JF716940	X2	X2	X	183C 189 223 278 325 519	73 152 195 	0
Ancona	ACK080D	JF716941	T1a	T1	T	126 163 186 189 286 294 519	73 152 195 	0
Ancona	ACK090D	JF716942	L1b	L1b	L	126 187 189 223 264 270 278 284 293 311 360 519	73 152 182 185 195 	0
unspecified	ACK100D	JF716943	T2b	T2	T	126 256 294 296 304 519	73 150 151 	1	Nephropathy, Renal Failure
Ancona	ACL010D	JF716944	H	H	H	519	rCRS	0
Ancona	ACL020D§	JF716945	U5a1	U5	U	256 270 294 399	73 152 	0
unspecified	ACL050D	JF716946	H	H	H	184 263 265 	207	1
Ancona	ACL070D	JF716947	I	I	N1-2	129 223 391 519	73 152 199 204 207 	1
Ancona	ACL090D	JF716948	H	H	H	rCRS	rCRS	1	Retinopathy
Ancona	ACL100D§	JF716949	H	H	H	rCRS	56+C 	1
Ancona	ACM010D	JF716950	H1	H1	H	519	60A 152 	1	Retinopathy, Somatic Neuropathy
Ancona	ACM020D	JF716951	H	H	H	rCRS	rCRS	1	Nephropathy
Ancona	ACM030D	JF716952	T2	T2	T	126 294 296 312 519	73 146 	1	Nephropathy
Ancona	ACM040D	JF716953	H	H	H	086 311	152	1
Ascoli Piceno	ACM050D	JF716954	T2	T2	T	126 294 519	73 195 	1	Somatic Neuropathy
Ancona	ACM060D	JF716955	D4	D4	M	092 188 223 362 	73 197 	1	Nephropathy, Renal Failure
unspecified	ACM080D	JF716956	T1a	T1	T	126 163 186 189 294 519 555	73 152 195 	0
Ancona	ACM090D	JF716957	T2b	T2	T	126 294 296 304 519	73	1	Retinopathy, Cardiac Ischemia, Nephropathy, Renal Failure
Ancona	ACM100D	JF716958	T1a	T1	T	126 163 186 189 294 519	73	1	Somatic Neuropathy
Ancona	ACN020D	JF716959	H	H	H	519	rCRS	0
Ancona	ACN030D§	JF716960	W	W	N1-2	223 292 318 519	73 189 194 195 204 207 	1	Somatic Neuropathy, Cardiac Ischemia, Nephropathy
Ancona	ACN040D	JF716961	U1a	U1	U	182C 183C 189 242 249 266h 362 527	73	0
Ancona	ACN050D§	JF716962	X2	X2	X	189 223 278 519	73 195 204 207 	1
Ancona	ACN060D	JF716963	I	I	N1-2	111G 129 223 391 519	73 199 204 	0
Ancona	ACN070D	JF716964	H1	H1	H	188G 519 527	rCRS	1	Somatic Neuropathy
unspecified	ACN080D§	JF716965	U5a1	U5	U	192 256 270 291 399 519	73	0
Ancona	ACN090D	JF716966	K1	K1	K	224 311 519	73	0
Ancona	ACO010D§	JF716967	W	W	N1-2	223 234 292 519	73 189 194 195 204 207 	1
Ancona	ACO020D§	JF716968	U5a1	U5	U	192 256 270 291 399 519	73	0
Ancona	ACO030D	JF716969	M1a1	M1	M	129 189 223 249 311 359 519	73 195 	0
Ancona	ACO060D§	JF716970	U5a1	U5	U	256 270 293 399	73 189 	0
Ancona	ACO070D	JF716971	H	H	H	519	rCRS	1
Ancona	ACO080D§	JF716972	V	V	HV	298 519	72	0
Macerata	ACO100D	JF716973	H	H	H	278 293 311	195	1
Ancona	ACP010D§	JF716974	U5a1	U5	U	172 192 256 270 399	73	1
Ancona	ACP020D	JF716975	T2c	T2	T	126 292 294 519	73 146 152 	0
Ancona	ACP030D	JF716976	HV0*	HV0	HV	129 298 	72 195 	1
Ancona	ACP050D	JF716977	T2c	T2	T	292 294 296 380 519	73 146 	0
unspecified	ACP060D	JF716978	H5	H5	H	192 304 311	rCRS	1
Ancona	ACP090D	JF716979	H1c	H1	H	158 519	rCRS	1
Ancona	ACP100D	JF716980	H	H	H	519	rCRS	0
Ancona	ACQ010D	JF716981	U3a	U3	U	343 390	73 150 	1	Nephropathy, Renal Failure
Macerata	ACQ030D	JF716982	H	H	H	519	rCRS	1	Retinopathy, Somatic Neuropathy, Cardiac Ischemia, Peripheral Artery Occlusive Disease
Macerata	ACQ060D	JF716983	H3	H3	H	519	rCRS	0
Ancona	ACQ070D	JF716984	T2a	T2	T	092A 126 294 296 324 519	73	0
Ancona	ACQ090D	JF716985	H1	H1	H	270 519	rCRS	0
Ancona	ACQ100D	JF716986	H1	H1	H	270 519	rCRS	1
Ancona	ACR010D	JF716987	HV	HV	HV	217	72 73 152 195 	0
Ancona	ACR020D	JF716988	H1	H1	H	189 311 519	rCRS	1	Retinopathy, Peripheral Artery Occlusive Disease
Ancona	ACR030D	JF716989	H	H	H	213 362	195	1	Somatic Neuropathy
Ancona	ACR040D	JF716990	T2b	T2	T	294 296 304 365 519	73	1
Ancona	ACR060D	JF716991	T2b	T2	T	093 126 294 296 304 519 527	73	1
Ancona	ACR070D	JF716992	J1c	J1	J	069 126 390	73 185 	0
Ancona	ACR080D§	JF716993	U5a1	U5	U	192 256 270 301 399	73	0
Ancona	ACR090D	JF716994	H6b	H6	H	300 362 482	44+C 152 189 214	1	Nephropathy
Ancona	ACS010D	JF716995	H1	H1	H	183C 189 519	rCRS	1	Cardiac Ischemia
Macerata	ACS020D	JF716996	H	H	H	145 519	rCRS	1	Retinopathy, Cardiac Ischemia
Ancona	ACS030D	JF716997	H	H	H	519	152	0
Ancona	ACS040D	JF716998	H5	H5	H	304	rCRS	0
Ancona	ACS050D	JF716999	J1c	J1	J	048 069 114 126 215 265	73 185 	0
Ancona	ACS070D§	JF717000	U5b	U5	U	270	73 150 199 	1	Somatic Neuropathy
Ancona	ACS090D§	JF717001	V	V	HV	298 527	72	0
unspecified	ACS100D	JF717002	J2a	J2	J	069 126 145 193 278	73 150 152 	1
Ancona	ACT010D	JF717003	T2c	T2	T	126 146 292 294 519	73 150 	0
Ancona	ACT020D	JF717004	H1	H1	H	093 519	195	0
Ancona	ACT030D§	JF717005	U5a	U5	U	192 256 270 390 526	73	0
Ancona	ACT040D	JF717006	H	H	H	189 284 286 519	rCRS	1	Retinopathy, Somatic Neuropathy
Ancona	ACT060D	JF717007	T2h	T2	T	126 153 294 362 519	41 73 150 	1
Ancona	ACT070D	JF717008	H5	H5	H	111G 304	rCRS	0
Ancona	ACT080D	JF717009	U9	U9	U	114	73 146 195 	1	Retinopathy, Somatic Neuropathy
Ancona	ACT090D	JF717010	H	H	H	274 287 519	rCRS	0
Ancona	ACT100D	JF717011	T2b	T2	T	126 292 294 296 304 311 346 519	73	1
Macerata	ACU020D	JF717012	J2a	J2	J	069 126 193 278 	73 150 152 195 215 	1
Ancona	ACU030D§	JF717013	X2	X2	X	183C 189 223 278 325 519	73 152 195 	1
Ancona	ACU040D	JF717014	J1c	J1	J	069 126 390	73 185 	0
Ancona	ACU050D	JF717015	H5	H5	H	304 519	152	0
Ancona	ACU060D	JF717016	T2b	T2	T	093 126 294 296 304 519 527	73	1
Ancona	ACU070D§	JF717017	U5a1	U5	U	173 192 256 270 311 399	73	1	Retinopathy
Ancona	ACU080D	JF717018	H	H	H	092 293 311 	195	1	Somatic Neuropathy
Ancona	ACU090D	JF717019	J2b	J2	J	069 126 145 193 278	73 150 152 	1
Ancona	ACV010D	JF717020	H1	H1	H	188G 519 527	rCRS	1
Ancona	ACV050D	JF717021	H	H	H	291 311	195	0
Ancona	ACV060D	JF717022	T2c	T2	T	126 146 292 294 519	73 150 	0
Ancona	ACV090D§	JF717023	U3b	U3	U	311	73 150 152 	0
Ancona	ACV100D	JF717024	H1	H1	H	320 519	146	0
unspecified	ACW020D	JF717025	K2	K2	K	224 311 519	73 146 152 	0
Ancona	ACW030D	JF717026	U1a	U1	U	182C 183C 189 249 	73	1
Ancona	ACW040D	JF717027	R0a	R0a	R0	126 266 362	58 60+T 64 	1	Somatic Neuropathy
Ancona	ACW050D	JF717028	I	I	N1-2	111G 129 223 391 519	73 199 204 	1
Ancona	ACW060D§	JF717029	U5a1	U5	U	189 192 256 270 291 399	73	1	Cardiac Ischemia
Macerata	ACW070D	JF717030	H	H	H	192 186	rCRS	0
Ancona	ACW080D	JF717031	HV1	HV	HV	067 265	rCRS	1	Cardiac Ischemia
Macerata	ACW090D	JF717032	H	H	H	rCRS	rCRS	1	Retinopathy, Nephropathy
unspecified	ACW100D	JF717033	H1c	H1	H	519	152	1	Retinopathy, Nephropathy, Peripheral Artery Occlusive Disease
Ancona	ACY010D	JF717034	I	I	N1-2	129 223 260 391 519	73 152 199 204 207 	1
Ancona	ACY020D	JF717035	H5	H5	H	086 214 304	146	1	Nephropathy, Peripheral Artery Occlusive Disease
Ancona	ACY030D	JF717036	L3e2b	L3	L	172 183C 189 223 288 320 519	73 150 195	0
Ancona	ACY050D	JF717037	H1	H1	H	188G 519	rCRS	1	Retinopathy, Nephropathy
Ancona	ACY070D	JF717038	H	H	H	189 342	rCRS	1	Retinopathy, Cardiac Ischemia, Nephropathy
Ancona	ACY100D	JF717039	H	H	H	362	150	1	Retinopathy
Ancona	ACZ010D	JF717040	H1	H1	H	189 356 519	rCRS	0
Ancona	ACZ020D	JF717041	H	H	H	189 220C 261 312	188	0
Ancona	ACZ030D	JF717042	J1c	J1	J	069 126 235 519	73 185 	1	Somatic Neuropathy, Cardiac Ischemia
Ancona	ACZ050D§	JF717043	U5a	U5	U	114A 173 192 256 270 294 526	73	1
Ancona	ACZ060D	JF717044	H	H	H	192	186	1
Ancona	ACZ070D	JF717045	H1	H1	H	093 519	195	0
Ancona	ACZ080D	JF717046	H	H	H	114 319 519	rCRS	0
Ascoli Piceno	ACZ090D	JF717047	T2c	T2	T	126 146 292 294 519	73 150 	1
Ancona	ADA010D	JF717048	H	H	H	362	150	1	Retinopathy, Somatic Neuropathy, Nephropathy
Ancona	ADA020D§	JF717049	U5a1	U5	U	256 270 294 399	73 152 	1
Ancona	ADA040D§	JF717050	U5a1	U5	U	256 270 294 399	73 152 	1	Cardiac Ischemia
Ancona	ADA060D	JF717051	H1	H1	H	172 183C 189 213 519	150	1	Retinopathy, Cardiac Ischemia
Macerata	ADA080D	JF717052	H	H	H	rCRS	rCRS	0
Ancona	ADA090D§	JF717053	V	V	HV	298	72	0
Ancona	ADA100D	JF717054	T2b	T2	T	126 294 296 304 519	73	1
Ancona	ADB010D	JF717055	H	H	H	218 519	rCRS	1
Ancona	ADB020D	JF717056	H	H	H	rCRS	rCRS	0
Ancona	ADB030D	JF717057	T1a	T1	T	126 163 186 189 294 519	73 152 195 	0
Ancona	ADB050D§	JF717058	U3b	U3	U	343	73 150 	0
Ancona	ADB070D	JF717059	HV    	HV	HV	294 519	146	1
Ancona	ADB080D	JF717060	H1	H1	H	519	rCRS	0
Ancona	ADB090D§	JF717061	U3b	U3	U	092 104 343 519	73 146 150 	1
unspecified	ADB100D§	JF717062	W	W	N1-2	223 292 519	73 189 194 195 204 207 	1	Retinopathy
Ancona	ADC010D	JF717063	U3a	U3	U	343 390 519	73 150 	1	Somatic Neuropathy, Cardiac Ischemia
Ancona	ADC020D§	JF717064	X2	X2	X	150 166h 183C 188 189 223 278 519	73 153 195 	1
Ancona	ADC040D§	JF717065	X2	X2	X	183C 189 223 278 519	73 153 195 	1
Ancona	ADC050D	JF717066	U4	U4	U	356 362 519	73 195 	1	Retinopathy, Cardiac Ischemia
Ancona	ADC060D	JF717067	R0a	R0a	R0	126 266 362	58 60+T 64 	1
Ancona	ADC070D	JF717068	T1a	T1	T	126 163 176 186 189 294 311 519	73 146 150 152 	1	Retinopathy, Somatic Neuropathy, Cardiac Ischemia, Peripheral Artery Occlusive Disease
Ancona	ADC080D	JF717069	T2	T2	T	093 126 266 294 296 519	73	1
Ascoli Piceno	ADC090D§	JF717070	V	V	HV	298 335 	72	0
Ancona	ADC100D§	JF717071	W	W	N1-2	223 234 292 519	73 189 194 195 204 207 	1
Ancona	ADD020D	JF717072	T2c	T2	T	126 146 292 294 519	73 150 	0
Ancona	ADD030D	JF717073	K1	K1	K	224 246T 311 519	73 152 	0
Ancona	ADD040D	JF717074	T2b	T2	T	126 294 296 304 519 527	73	0
Ancona	ADD050D	JF717075	T1a	T1	T	126 163 186 189 294 519	73 152 	1	Nephropathy
Ancona	ADD060D	JF717076	U3a	U3	U	343 390 519	73 150 185 	1	Cardiac Ischemia, Nephropathy
Ancona	ADD070D	JF717077	H1	H1	H	519	rCRS	0
Ancona	ADD080D	JF717078	J1c	J1	J	069 126	73 185 189	0
Macerata	ADD090D	JF717079	H3	H3	H	519	152	1	Retinopathy, Cardiac Ischemia
Ancona	ADD100D	JF717080	U3a	U3	U	343 390 519	73 150 185 	1
unspecified	ADE050D	JF717081	H1	H1	H	189 519	rCRS	1	Retinopathy
unspecified	ADE080D	JF717082	J2a	J2	J	069 126 169	73 150 195	1	Cardiac Ischemia
Ancona	ADE090D	JF717083	HV1	HV	HV	067 129 274 368	rCRS	1	Retinopathy
Ancona	ADF010D	JF717084	J1c	J1	J	069 126	73 185 188 	1
Ancona	ADF040D	JF717085	H	H	H	261 291 519	200	1
Ancona	ADF060D	JF717086	H	H	H	093	rCRS	1	Retinopathy, Somatic Neuropathy, Cardiac Ischemia
Ancona	ADF070D	JF717087	H	H	H	362	150 200 	1	Retinopathy, Somatic Neuropathy, Nephropathy, Peripheral Artery Occlusive Disease
Ancona	ADF100D	JF717088	K1	K1	K	224 311 519	73 94 	0
Ancona	ADG010D	JF717089	K1	K1	K	093 172 224 311 354 519	73 199 204 	0
unspecified	ADG020D	JF717090	H6	H6	H	362 482	73	1
Ancona	ADG030D	JF717091	H1	H1	H	519	152	0
unspecified	ADG040D	JF717092	H3	H3	H	519	152	1	Retinopathy, Somatic Neuropathy
Ancona	ADG050D	JF717093	T2b	T2	T	126 258C 294 296 304 519	73	1	Cardiac Ischemia
Ancona	ADG060D§	JF717094	U3b	U3	U	051 093 343	73 150 	0
Ancona	ADG080D	JF717095	H	H	H	218 519	93	1	Cardiac Ischemia
Ancona	ADG090D	JF717096	H1	H1	H	312 519	rCRS	0
unspecified	ADG100D§	JF717097	V	V	HV	189 298	72 194 	1
Ancona	ADH010D	JF717098	K1	K1	K	093 172 224 311 354 519	73 204 	1
Ancona	ADH020D	JF717099	L3e2b	L3	L	172 183C 189 223 288 320 519	73 150 195	0
Ancona	ADH030D	JF717100	J1c	J1	J	069 126 519	73 146 185 188 	0
Ancona	ADH050D	JF717101	H1	H1	H	086 188G 519	195	0
Ancona	ADH060D	JF717102	H1	H1	H	037 175 213 519	rCRS	0
Ancona	ADH070D§	JF717103	U5b	U5	U	270	73 150 204 207 	0
Ancona	ADH080D	JF717104	K1	K1	K	093 224 311 519	73 152 	1
Ancona	ADH090D	JF717105	H	H	H	519	rCRS	1
unspecified	ADH100D	JF717106	H5	H5	H	304 362	152	0
Macerata	ADI010D§	JF717107	U5b	U5	U	172 174 189 260 270 311 360	73 150 	1	Somatic Neuropathy
Ancona	ADI020D	JF717108	T2	T2	T	126 189 294 296 519	73 146 	1
Ancona	ADI030D	JF717109	H1	H1	H	519	152	1
Ancona	ADI040D	JF717110	H	H	H	274 287 519	rCRS	0
Ancona	ADI050D	JF717111	T2	T2	T	126 189 294 296 519	73 146 	1	Nephropathy
Ancona	ADI060D	JF717112	T2	T2	T	126 140 189 294 296 311 519	73	1	Somatic Neuropathy, Cardiac Ischemia
unspecified	ADI080D§	JF717113	U5a1	U5	U	256 270 399	73	1	Retinopathy
Ancona	ADJ010D§	JF717114	U7	U7	U	309 318T 519	73 152 185C 	1	Cardiac Ischemia
Ancona	ADJ030D	JF717115	J1c	J1	J	069 126 311 319 184h	73 185 	1	Nephropathy
Ancona	ADJ040D§	JF717116	X2	X2	X	189 223 248 278 519	73 153 195 	1	Retinopathy
Ancona	ADJ060D	JF717117	K1	K1	K	093 224 311 519	73 146 195 	1	Retinopathy, Somatic Neuropathy, Cardiac Ischemia, Nephropathy
Ancona	ADJ070D	JF717118	I	I	N1-2	111 129 223 391 519	73 150 199A 204	0
Ancona	ADJ080D	JF717119	T1a	T1	T	126 163 176 186 189 294 311 519	73 146 152 	0
Ancona	ADJ090D	JF717120	J2a	J2	J	069 126 169	73 150 195 	1	Somatic Neuropathy
Ancona	ADK020D§	JF717121	H1	H1	H	188G 519	rCRS	1
Ancona	ADK030D	JF717122	H	H	H	154 219 325 519	73	1	Nephropathy
Ancona	ADK040D§	JF717123	U5a1	U5	U	256 270 399	73 206G 	1	Retinopathy, Cardiac Ischemia
Ancona	ADK060D	JF717124	H6	H6	H	362 482	152	0
Ancona	ADK070D	JF717125	T1a	T1	T	126 163 186 189 294 519	73 152 	1
Ancona	ADK080D	JF717126	T2b	T2	T	126 294 296 304 519 527	73	1
Ancona	ADK090D	JF717127	H1c	H1	H	519	rCRS	1	Cardiac Ischemia
unspecified	ADK100D	JF717128	H	H	H	311 519	rCRS	1	Peripheral Artery Occlusive Disease
Ancona	ADL010D	JF717129	H1	H1	H	209 519	rCRS	0
Ancona	ADL020D	JF717130	J1c	J1	J	069 126	73	1
Ancona	ADL030D	JF717131	K1	K1	K	224 300 311 519	73	1	Retinopathy
Ancona	ADL050D	JF717132	H1	H1	H	519	146	0
Ancona	ADL080D	JF717133	H3	H3	H	519	rCRS	0
Ancona	ADL100D	JF717134	J1c	J1	J	069 126 319 343	73 185 	0
Ancona	ADM010D	JF717135	T2c	T2	T	086 126 292 294 296 519	73 89 146 	0
Ancona	ADM020D	JF717136	M1b2	M1	M	093 129 182C 183C 189 223 249 311 399 519	73 195	0
Ancona	ADM030D	JF717137	U3b	U3	U	343	73 150 	0
Ancona	ADM070D	JF717138	H	H	H	311	152	1	Nephropathy, Renal Failure
Ancona	ADM080D	JF717139	K1	K1	K	224 311 519	73	1
Ancona	ADM090D	JF717140	H	H	H	292 519	rCRS	0
Ancona	ADM100D§	JF717141	U5a	U5	U	243 256 270 524h 526	73 152 	1
Ancona	ADN010D	JF717142	H3h	H3	H	311 519	rCRS	1	Retinopathy, Cardiac Ischemia
Ancona	ADN020D§	JF717143	U5a1	U5	U	256 270 399	73	0
Macerata	ADN040D	JF717144	H1	H1	H	256 319 519	93	0
unspecified	ADN050D	JF717145	U4	U4	U	134 274 356 519	73 152 195 	0
Ancona	ADN060D	JF717146	HV	HV	HV	308 311	146	0
Ancona	ADN080D	JF717147	K1	K1	K	224 311 519	73 146 152 	0
Ancona	ADN090D	JF717148	HV1	HV	HV	067 183C 189	152	0
Ancona	ADO010D	JF717149	K1	K1	K	224 311 519	73	0
Ancona	ADO030D	JF717150	J1c	J1	J	069 126 182C 183C 189	73 185	1
unspecified	ADO040D§	JF717151	X2	X2	X	189 217 223 278 519	73 143 195	0
Ancona	ADO060D	JF717152	K1	K1	K	093 224 311 519	73	1	Retinopathy, Cardiac Ischemia
Ancona	ADO070D	JF717153	N1c	N1	N1-2	111 201 223 265 519	73 189 195 204 207 210 	0
Ancona	ADO090D§	JF717154	U3b	U3	U	343 519	73 150 	0
Ancona	ADO100D	JF717155	H	H	H	093	rCRS	0
Ancona	ADP040D	JF717156	H5	H5	H	186 214 304	146	0
Ancona	ADP050D	JF717157	H1	H1	H	519	95C 	0
Ancona	ADP070D	JF717158	H5	H5	H	304 390	rCRS	1
Ancona	CAD1000GM	JF717159	J1c	J1	J	069 111 126	73 185 	0
Ancona	CAD1001FA	JF717160	T1a	T1	T	126 163 186 189 294 519	73 152 195 	0
Ancona	CAD1002ML§	JF717161	X2	X2	X	183C 189 223 274 278 519	73 143 195	0
Ancona	CAD1003BF	JF717162	T2b	T2	T	294 296 304 365 519	73	0
Ancona	CAD1004MD§	JF717163	U5b	U5	U	192 270	73 150 199 	0
Ancona	CAD1005ZS	JF717164	H	H	H	rCRS	146	0
Ancona	CAD1006SI	JF717165	H1	H1	H	519	rCRS	0
unspecified	CAD1007IP	JF717166	I	I	N1-2	129 148 223 391 519	73 199 	0
Ancona	CAD1016PS	JF717167	H	H	H	142 325	146	0
Ancona	CAD1017NN	JF717168	H5	H5	H	304 311	93	0
Ancona	CAD1019PP	JF717169	T2b	T2	T	126 189 294 296 304 519	73 151	0
Ancona	CAD1020DM	JF717170	H3h	H3	H	311 519	rCRS	0
Ancona	CAD1022MS	JF717171	HV	HV	HV	311	rCRS	0
Ancona	CAD1023CM	JF717172	H	H	H	519	rCRS	0
Ancona	CAD1025NZ§	JF717173	U5b	U5	U	192 270	73 150 199 	1
Ancona	CAD1026BA	JF717174	HV	HV	HV	311	195	0
Ancona	CAD1031CA	JF717175	U4	U4	U	134 274 356 519	73 152 195 	0
Ancona	CAD1033TA	JF717176	J1c	J1	J	069 126 390	73 185 188 	0
Ancona	CAD1034PM	JF717177	H1	H1	H	051 189 356 519	rCRS	0
Ancona	CAD1037PD	JF717178	T1a1	T1	T	126 163 176 186 189 294 311 519	73 146 150 152 	0
Ancona	CAD1038CG§	JF717179	X2	X2	X	183C 189 223 278 519	73 150 189 195	0
Ancona	CAD1039ML	JF717180	J1c	J1	J	069 126 189 	73 185	0
Ancona	CAD1040NN	JF717181	H	H	H	183C 189 519	146 153 204	0
unspecified	CAD1042TM	JF717182	H	H	H	519	93	0
Ancona	CAD1043BV	JF717183	H1	H1	H	293 304 326	rCRS	0
Ancona	CAD1044AR	JF717184	T2b	T2	T	126 292 294 296 304 311 346 519	73	0
Ancona	CAD1045BL	JF717185	K1	K1	K	224 311 519	73 199 	0
Ancona	CAD1046LB	JF717186	M1b2	M1	M	093 129 182C 183C 189 223 249 311 399 519	73 195	0
Ancona	CAD1047MN	JF717187	T2a	T2	T	126 294 296 324 519	73	0
Ancona	CAD1048PC	JF717188	H	H	H	274 287 519	rCRS	1
Ancona	CAD1049TB	JF717189	H1	H1	H	093 519	rCRS	0
Ancona	CAD1050MM	JF717190	H	H	H	183C 189 248h 519	146 153 204	0
Macerata	CAD1052FT	JF717191	T2	T2	T	126 294 296 519	73	0
Ancona	CAD1053PB	JF717192	H	H	H	192	186A 	0
Ancona	CAD1054TM	JF717193	J1c	J1	J	069 126 390	73 185 188 	1
unspecified	CAD1055SP	JF717194	H1	H1	H	093 519	rCRS	0
Ancona	CAD1057DGO	JF717195	H	H	H	311 519	rCRS	0
Ancona	CAD1058PM§	JF717196	U5a1	U5	U	256 270 399	73 146 207 	0
Ancona	CAD1059PA§	JF717197	U5b3	U5	U	270 304 311	73 150 	0
Ancona	CAD1060MM	JF717198	H	H	H	311	195	0
Ancona	CAD1061TM	JF717199	H1	H1	H	519	rCRS	0
Ancona	CAD1062TG	JF717200	K2	K2	K	224 311 519	73 146 152 	0
Macerata	CAD1063FR	JF717201	H3	H3	H	278 519	260	0
Pesaro Urbino	CAD1064MA	JF717202	H	H	H	249 519	152	0
Ancona	CAD1065GZ	JF717203	U3b	U3	U	311	73 150 152 	0
Ancona	CAD1066FM	JF717204	HV	HV	HV	311 357	146	0
Ancona	CAD1068RG	JF717205	N1b1	N1	N1-2	145 176G 223 244 390 519	73 146 	1	Retinopathy, Somatic Neuropathy
Ancona	CAD1069TE	JF717206	K1	K1	K	224 311 519	73 195 	0
Fermo	CAD1070BG	JF717207	H	H	H	311 354	152	0
Pesaro Urbino	CAD1073BM	JF717208	H5	H5	H	304 362 	152	0
Ancona	CAD1076DA	JF717209	H6	H6	H	222 362 400 482	rCRS	1
Ancona	CAD1077DO	JF717210	K1	K1	K	224 300 311 519	73	0
Ancona	CAD1079MC§	JF717211	U5a1	U5	U	256 270 399	44+C 73 	0
Ancona	CAD1080PM	JF717212	T2b	T2	T	093 126 294 296 304 519	73	0
Ancona	CAD1083BP	JF717213	H5	H5	H	093 294 304 320	152	0
Ancona	CAD1084PP	JF717214	H	H	H	519	rCRS	0
Ancona	CAD1088BP	JF717215	T2c	T2	T	126 292 294 519	73 146 	0
Ancona	CAD1093DCG	JF717216	U1a	U1	U	183C 185 189 193d 249 365	73 185 	0
Ancona	CAD1094PS	JF717217	J1c	J1	J	069 126 390	73 185 	0
Ascoli Piceno	CAD1095LG	JF717218	J1c	J1	J	069 126 249	73 185 	0
Ancona	CAD1096MG	JF717219	J1c	J1	J	069 126	73 185 	0
Ancona	CAD1097RM§	JF717220	U4	U4	U	356 519	73 195 	1
Ancona	CAD1098AM	JF717221	H1	H1	H	519	rCRS	0
Ancona	CAD1099FK	JF717222	H1a	H1	H	051 162 519	73	0
Ancona	CAD1100BR	JF717223	K1	K1	K	223 224 311 519	73	1
Ancona	CAD1101CM	JF717224	H	H	H	131h 183C 189 193+C 284 286 519	rCRS	0
Ancona	CAD1103BA	JF717225	H5	H5	H	304	rCRS	1
Ancona	CAD1104SM	JF717226	T2c	T2	T	126 292 294 519	73 146 	0
Ancona	CAD1106CC	JF717227	H	H	H	519	rCRS	0
Ancona	CAD1107TA	JF717228	U3b	U3	U	343 	73 150 	0
Ancona	CAD1108VML§	JF717229	U5a1	U5	U	192 256 270 399	73 195 	0
Ancona	CAD1110AL	JF717230	H1	H1	H	519	rCRS	0
Ancona	CAD1111PP	JF717231	HV	HV	HV	311	rCRS	0
Pesaro Urbino	CAD1112MS	JF717232	H1	H1	H	260	rCRS	1	Nephropathy
Macerata	CAD1113FT	JF717233	T2	T2	T	126 294 296 519	73	0
Ancona	CAD1115IMP	JF717234	N1b1	N1	N1-2	145 176G 223 390 519	73 152 	1
Ancona	CAD1116FD§	JF717235	U5a1	U5	U	192 256 270 399	73 195 	0
Macerata	CAD1117AM	JF717236	T2c	T2	T	094 126 292 294 319 519	73 146 152 	0
Ancona	CAD1118ND	JF717237	U2e	U2	U	051 126 129C 189 256 318 362 519	73 152 	0
Ancona	CAD1120AM	JF717238	H1	H1	H	158 519	rCRS	0
Ancona	CAD1121UC	JF717239	U1a	U1	U	183C 185 189 193d 249 365	73 185 	0
Ancona	CAD1122MI	JF717240	H1	H1	H	519	rCRS	0
Ancona	CAD1123TA	JF717241	T2	T2	T	126 220T 294 296 304 519	73	0
Ancona	CAD1125MMC§	JF717242	V	V	HV	239 298 399	72	0
Ancona	CAD1128TAM	JF717243	H1	H1	H	293 519	93	0
Ancona	CAD1130BV	JF717244	H1	H1	H	183C 189 193+3C 519	rCRS	0
unspecified	CAD1131RA	JF717245	H1	H1	H	519	rCRS	0
Ancona	CAD1132AA	JF717246	H1	H1	H	158 519	rCRS	0
Ancona	CAD1133BR	JF717247	H	H	H	093 274 	56+C 93 183 	1
Ancona	CAD1134PAM	JF717248	H9	H9	H	168 325 519	152	0
Ancona	CAD1135BL	JF717249	H1	H1	H	183C 189 193+2C 519	rCRS	1
Ancona	CAD1136ZC	JF717250	J1c	J1	J	069 126 311 319	73 185 	0
Ancona	CAD1139MO	JF717251	W	W	N1-2	093 192 223 292 325 519	73 189 194 195 204 207 	0
Ancona	CAD1140PP§	JF717252	X2	X2	X	150 183d 188 189 223 278 519	73 153 195 	0
Ancona	CAD1141MAM	JF717253	H	H	H	519	152	0
Ancona	CAD1142TA	JF717254	H1	H1	H	293 519	93	0
Macerata	CAD1144TO	JF717255	H6	H6	H	172 362 482	rCRS	0
Ancona	CAD1145PM§	JF717256	X2	X2	X	189 223 278 519	73 153 195	0
Ancona	CAD1146TP	JF717257	H	H	H	261 519	rCRS	0
Ancona	CAD1147MM	JF717258	H1	H1	H	519	rCRS	0
Ancona	CAD1150GF	JF717259	H	H	H	189	rCRS	0
Ancona	CAD1152AA	JF717260	T2c	T2	T	126 146 292 294 519	73 150 	0
Ancona	CAD1159RL	JF717261	H5	H5	H	304	rCRS	0
Ancona	CAD1160PM	JF717262	J1c	J1	J	069 126 390	73 185 	0
Ancona	CAD1161AE	JF717263	H1	H1	H	256 519	204 207 	0
Ancona	CAD1162PL	JF717264	T2b	T2	T	294 296 304 365 519	73	1
Ancona	CAD1163BP	JF717265	T2h	T2	T	126 153 294 296 519	73 150 	1
Ancona	CAD1164BD	JF717266	J2b	J2	J	069 093 126 193 442 443 	73 150 152 207 	0
Ancona	CAD1165BG	JF717267	HV	HV	HV	311	152	1
Ancona	CAD1166BG	JF717268	J1c	J1	J	069 126 357	73 182 185 	0
Ancona	CAD1167GG	JF717269	H	H	H	519 553	rCRS	0
Ancona	CAD1168PL	JF717270	N1b1	N1	N1-2	145 176G 188 223 390 465A 519	73 151 152 200 	0
Ancona	CAD1171SG§	JF717271	X2	X2	X	183C 189 193+C 223 278 325 519	73 152 195	0
Ancona	CAD1174SD	JF717272	H1	H1	H	519	rCRS	1
Ancona	CAD1176CD	JF717273	H	H	H	218 519	rCRS	0
Ancona	CAD1178SR§	JF717274	U5a1	U5	U	192 256 270 399	73 195 	1
Ancona	CAD1179MD	JF717275	T1a	T1	T	126 163 186 189 294 519	73 152 195 	0
Ancona	CAD1181TW	JF717276	H1	H1	H	293 519	93	0
Ancona	CAD135MR	JF717277	H6	H6	H	362 482	rCRS	0
Ancona	CAD908PL§	JF717278	V	V	HV	290 298	72	0
Ancona	CAD909BG§	JF717279	U5a1	U5	U	192 256 270 390 526	73	0
Ancona	CAD910BR	JF717280	T2b	T2	T	126 294 296 304 519 527	73	0
Ancona	CAD911AM	JF717281	H	H	H	519	93	0
Ancona	CAD912PG	JF717282	U4	U4	U	356 519	73 146 152 195 207	0
Ancona	CAD913BR	JF717283	H1	H1	H	519	95C 	0
Ancona	CAD914MC§	JF717284	W	W	N1-2	223 292 295 324 519	73 189 195 204 207	0
Ancona	CAD915ZF	JF717285	H	H	H	519	rCRS	0
Ancona	CAD916BG	JF717286	W	W	N1-2	223 292 318 519	73 189 194 195 204 207 	0
Macerata	CAD917CG	JF717287	H6	H6	H	172 362 482	rCRS	0
Ancona	CAD918MP	JF717288	K1	K1	K	224 311 519	73	0
unspecified	CAD919PL	JF717289	J1c	J1	J	069 126 182C 183C 189	73 185	0
Macerata	CAD920TL	JF717290	J2b	J2	J	069 126 193 519	73 152 171C 	0
unspecified	CAD921CS§	JF717291	U3b	U3	U	343 	73 150 152 	0
Ancona	CAD922CS	JF717292	J1c	J1	J	069 126 182C 183C 189	73 185	0
Ancona	CAD923PG	JF717293	H1	H1	H	519	rCRS	0
unspecified	CAD924SG	JF717294	J1c	J1	J	069 126 260 	73 185 	0
Ancona	CAD925PF	JF717295	H1	H1	H	519	rCRS	0
Ancona	CAD926SF	JF717296	U4	U4	U	356 519	73 195 	0
Ancona	CAD927MF	JF717297	H	H	H	093	rCRS	0
Macerata	CAD928CF	JF717298	T2a	T2	T	092A 126 294 296 324 519	73	0
Ancona	CAD929PG	JF717299	H	H	H	519	rCRS	0
Ancona	CAD930MM	JF717300	H	H	H	092 293 301 311 	195	0
Ancona	CAD931CF	JF717301	H	H	H	261 519	rCRS	0
Macerata	CAD932SR	JF717302	J1b	J1	J	069 126 145 222 261 519	64 73 152 	0
unspecified	CAD933CM	JF717303	T2	T2	T	126 294 296 519	73	0
Ancona	CAD934TL	JF717304	H1	H1	H	239 390 519	rCRS	0
Ancona	CAD935BA	JF717305	U4	U4	U	356 519	73 146 151 152 195 207 	0
Ancona	CAD936FE	JF717306	T2	T2	T	126 294 296 312 519	73 146 	0
Ancona	CAD937LR	JF717307	H	H	H	265 298 	151 152 	0
Ancona	CAD938PM	JF717308	HV	HV	HV	217 	72 73 152 195 	0
Ancona	CAD939LL	JF717309	X2	X2	X	189 192 223 278 519	73 153 195 	0
Ancona	CAD940PG	JF717310	H3h	H3	H	311 519	rCRS	0
Ancona	CAD941RP	JF717311	J1c	J1	J	069 126 519	73 146 185 188 	0
Ancona	CAD943CA	JF717312	H	H	H	129 	207	0
Ancona	CAD945VD	JF717313	H1	H1	H	172 519	152	0
Ancona	CAD946PG	JF717314	H	H	H	274 287 519	rCRS	0
Ancona	CAD947BA	JF717315	T2b	T2	T	126 274 291 294 296 304 519	73	0
Ancona	CAD949FL	JF717316	T2	T2	T	126 294 296 312 519	73 146 	0
Ancona	CAD950MI	JF717317	HV	HV	HV	311 519	rCRS	0
Ancona	CAD951ML	JF717318	T1a	T1	T	126 163 186 189 294 354 519	73 152 195 	0
Ancona	CAD952CG	JF717319	H1	H1	H	209 355 519	150	0
Ancona	CAD954FF	JF717320	X2	X2	X	183C 189 193+C 223 278 519	73 131 153 195	0
Ancona	CAD955ZA§	JF717321	V	V	HV	298 519	72	0
unspecified	CAD956LG	JF717322	H	H	H	rCRS	rCRS	0
Ancona	CAD957CD	JF717323	H	H	H	129 	207	0
Ancona	CAD958CA§	JF717324	U5b3	U5	U	093 192 235 270 304	73 150 	0
Ancona	CAD959AS	JF717325	H1	H1	H	104 189 365 519	rCRS	0
Ancona	CAD961ZS	JF717326	U3a	U3	U	343 390 519	73 150 185 	0
Ancona	CAD962AF	JF717327	H1	H1	H	093 519	rCRS	1
Ancona	CAD963MA	JF717328	U8b	U8	U	183C 189 234 324 519	73 195	0
Ancona	CAD964LA	JF717329	T2	T2	T	126 176 183C 189 294 296 298 519	73 195	0
Ancona	CAD965FS	JF717330	H	H	H	519	195	0
Ancona	CAD966SS	JF717331	H	H	H	219 261 	rCRS	0
Ancona	CAD967EL	JF717332	H3	H3	H	311 519	rCRS	0
Ancona	CAD968CL	JF717333	K1	K1	K	129 145 224 311 519	73	0
Ancona	CAD969PM	JF717334	J1c	J1	J	069 126 182C 183C 189	73 185	1
Ancona	CAD971PD	JF717335	H8	H8	H	068 288 362	146 195	0
Ancona	CAD972PD	JF717336	U4	U4	U	242A 288 356 362 519	73 195 	0
Ancona	CAD974BA	JF717337	K1	K1	K	224 311 519	73 195 	1
Ancona	CAD976BT	JF717338	H	H	H	rCRS	146	0
Ancona	CAD977PU	JF717339	H1	H1	H	519	rCRS	0
Ancona	CAD978NL	JF717340	H	H	H	rCRS	rCRS	0
Ancona	CAD980BF	JF717341	T2b	T2	T	294 296 304 365 519	73	0
Ascoli Piceno	CAD981SR	JF717342	K1	K1	K	093 150 224 311 519	73 150 195 	0
Ancona	CAD983CP	JF717343	J1c	J1	J	069 126 319 343	73 185 	0
unspecified	CAD987MM	JF717344	H	H	H	086 311	152	0
Ancona	CAD988BP	JF717345	T2c	T2	T	126 292 294 519	73 146 	0
Ancona	CAD990RM§	JF717346	V	V	HV	298	72	0
Ancona	CAD991BD	JF717347	I	I	N1-2	075 129 169 223 260 391 519	73 199 204 	0
Ancona	CAD992GA§	JF717348	U5a1	U5	U	256 270 293 399	73	0
Ancona	CAD993AM	JF717349	H1	H1	H	158 519	rCRS	0
Ancona	CAD994MI	JF717350	H1	H1	H	519	rCRS	0
Ancona	CAD995PA	JF717351	H1	H1	H	188G 519 527	rCRS	0
unspecified	CAD996BG	JF717352	H	H	H	rCRS	146	0
Ancona	CAD997BS§	JF717353	U6a	U6	U	172 183C 189 219 278	44+C 73 103 143	0
Ancona	CAD999MG	JF717354	J1c	J1	J	069 126 249 	73 185 	0
 All control-region sequences were read from np 16024 to np 220 (for a total of 766 bp) with the exception of those sequences marked by the § symbol which were read from np 16040, and those marked by the † symbol which were read from np 16060.

## Sheet: Foglio2
